# Intra- and Intermolecular Charge-Transfer Dynamics of Carbene–Metal–Amide Photosensitizers

**DOI:** 10.1021/acs.jpcc.4c01994

**Published:** 2024-04-12

**Authors:** Michael S. Kellogg, Austin R. Mencke, Collin N. Muniz, Thabassum A. Nattikallungal, Fabiola Cardoso-Delgado, Nina Baluyot-Reyes, Marielle Sewell, Matthew J. Bird, Stephen E. Bradforth, Mark E. Thompson

**Affiliations:** † Department of Chemistry, University of Southern California, Los Angeles, California 90089, United States; ‡ Chemistry Division, Brookhaven National Laboratory, Upton, New York 11973, United States; § Department of Chemistry, University of California, Riverside, California 92521, United States

## Abstract

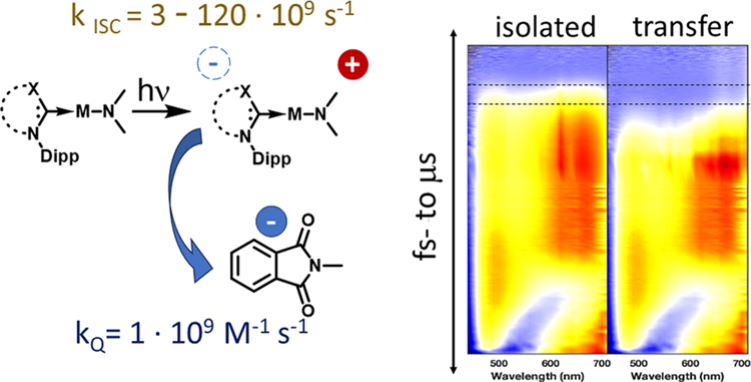

A series of steady-state and time-resolved spectroscopies were performed on a set of eight carbene–metal–amide (cMa) complexes, where M = Cu and Au, that have been used as photosensitizers for photosensitized electrocatalytic reactions. Using ps-to-ns and ns-to-ms transient absorption spectroscopies (psTA and nsTA, respectively), the excited-state kinetics from light absorption, intersystem crossing (ISC), and eventually intermolecular charge transfer were thoroughly characterized. Using time-correlated single photon counting (TCSPC) and psTA with a thermally activated delayed fluorescence (TADF) model, the variation in intersystem crossing (ISC), (*S*_1_ → *T*_1_) rates (∼3–120 × 10^9^ s^–1^), and Δ*E*_ST_ values (73–115 meV) for these compounds were fully characterized, reflecting systematic changes to the carbene, carbazole, and metal. The psTA additionally revealed an early time relaxation (rate ∼0.2–0.8 × 10^12^ s^–1^) attributed to solvent relaxation and vibrational cooling. The nsTA experiments for a gold-based cMa complex demonstrated efficient intermolecular charge transfer from the excited cMa to an electron acceptor. Pulse radiolysis and bulk electrolysis experiments allowed us to identify the character of the transient excited states as ligand–ligand charge transfer as well as the spectroscopic signature of oxidized and reduced forms of the cMa photosensitizer.

## Introduction

The need for the replacement of fossil fuels with renewable sources becomes more severe each day. While many renewable energy sources have been identified, solar energy is the most readily available source across the Earth.^1^ While the combination of photovoltaic (PV) panels and batteries is an efficient means to collect and store solar energy for use at a later time,^2^ storing the solar energy in the form of liquid or gaseous fuels would be advantageous from an energy density standpoint, especially for use in the transportation sector.^3,4^ Utilizing solar energy to drive the electrocatalytic transformation of abundant feedstocks such as water or CO_2_ to H_2_, CO, or methanol is an actively investigated approach to generating solar fuels.

**Figure 1:**
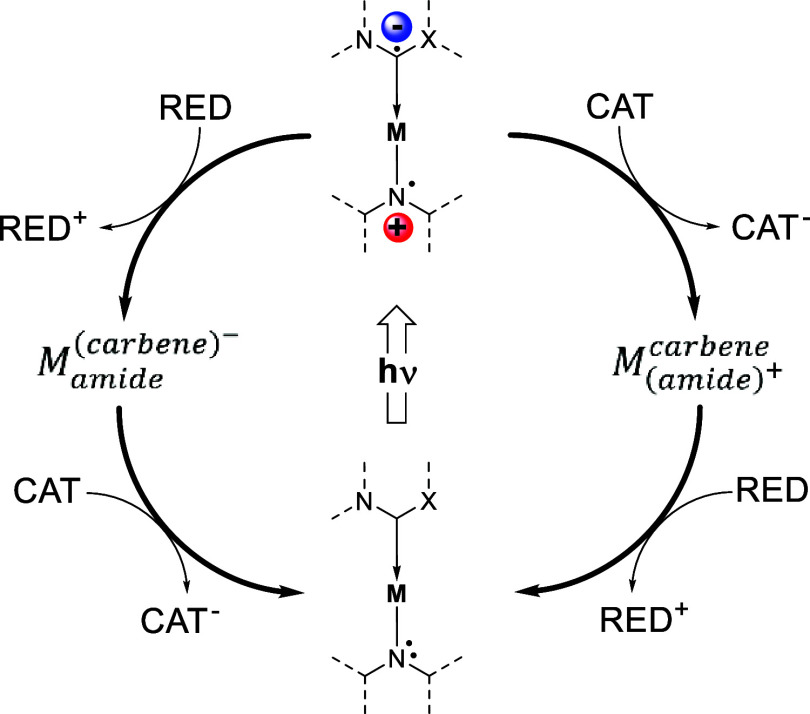
Schematic representation of a carbene–metal–amide (cMa, *M*_amide_^carbene^) being used as a sensitizer for a photoelectrocatalytic reduction reaction. The cycle begins with light excitation of the *M*_amide_^carbene^ to form the *M*_(amide)^+^_^(carbene)^−^^ excited state. The excited state then either donates an electron to a catalyst (CAT) (right path), and a reductant (RED) returns *M*_(amide)^+^_^carbene^ to the ground state. In the alternate path, (left path) the reductant captures *M*_(amide)^+^_^(carbene)^−^^, forming *M*_amide_^(carbene)^−^^, which has sufficient reducing potential to reduce the catalyst. The reductant can be either an electrode or a chemical reductant.

A common approach to producing these solar fuels is to couple a solar photosensitizer (PS) with an electrocatalyst (EC). Upon absorbing light, the photosensitizer (PS) is promoted to its excited state. The excited PS (PS*) is simultaneously a more potent oxidizing agent and a more potent reducing agent than the PS.^5^ Thus, the PS* can have sufficient chemical potential to oxidize or reduce an electrocatalyst (EC), driving the production of the fuel. The oxidized PS can then recover an electron from an electrode or sacrificial reductant (SAC) and repeat the photocatalytic cycle. The PS/EC/SAC cycle is summarized in Figure 1. A similar scheme can be constructed to describe a photo-oxidative process. This approach shows great promise as a homogeneous system for generating solar fuels from sunlight without the use of a PV to power the electrocatalysts.

For many years, the most prominent photosensitizers have been based on metal complexes involving noble metals, such as Ru and Ir.^6−9^ However, the low abundance and high expense of these elements make these heavy metal-based sensitizers an unsustainable solution. While monovalent copper complexes have also been shown to be good photosensitizers, the reported complexes are four-coordinate, which suffer from deactivation via large excited-state reorganizations, limiting their excited-state lifetime.^9−15^ In considering new and effective PS chromophores to study, a number of features are important. The potential PS must have (1) a strong, broad, and tunable absorption, (2) a long-excited-state lifetime to allow for the diffusion of PS* to the EC in solution, (3) stability in its photoexcited state and stability in its oxidized and/or reduced forms, and (4) high excited-state electrochemical potential for oxidation and/or reduction. Additionally, a PS is more sustainable if it is composed of only earth-abundant elements.

Recently, our group as well as others have reported photophysical and electroluminescent properties of linear, two-coordinate carbene–metal–amide (cMa) compounds where M = Cu, Ag, and Au with long lifetimes (0.2–3 μs) and strong, tunable absorption in the UV–visible region (ε > 10^3^ M^–1^ cm^–1^, λ_max_ = 350–600 nm).^16−20^ Herein, we report a study of the photophysics and photochemistry of a set of cMa complexes as representative of a larger cMa family previously described. The nature of the lowest-energy excited state in these compounds is an interligand charge-transfer (ICT) state, where the carbene acts as an acceptor and the amide acts as a donor to form the cMa ICT state, *M*_(amide)^+^_^(carbene)^−^^. The metal center remains monovalent in the excited state, so geometric distortions associated with moving from d^10^ to d^9^ are avoided. The energy of the ICT state can be tuned by the careful selection of each ligand’s electrochemical potential. These compounds emit from a thermally assisted delayed fluorescence (TADF) process leading to the long-excited-state lifetimes.^21,22^ The ICT nature of the excited state separates hole and electron instantaneously upon absorption. Utilizing an ICT state helps predispose the excited state to intermolecular charge transfer by adopting a molecular geometry akin to the cationic or anionic PS geometry, thus lowering the internal reorganization energy associated with charge transfer.^23^

In this paper, we fully investigate the establishment of the TADF equilibrium and intermolecular charge-transfer dynamics. Two of the materials studied here (i.e., Cu_BCz_^MAC^ and Au_BCz_^MAC^; Figure 1) have been used in a photoelectrocatalytic cycle as a photosensitizer, as illustrated in Figure 1.^24^ Here, we generate a full picture of the charge-transfer dynamics of a series of cMa complexes necessary to understand the photocatalysis, utilizing a range of spectroscopic techniques. Using time-correlated single photon counting (TCSPC) and pico-to-nanosecond transient absorption (psTA), we investigate intersystem crossing (ISC) rates and through the use of spectroelectrochemistry (SEC) we characterize both PS^+^ and PS^–^.^17,23,25,26^ Furthermore, we carried out a nano-to-microsecond (nsTA) study of intermolecular electron and hole transfer from a representative excited cMa to oxidants and reductants in solution, respectively.

## Methods

### Bulk Electrolysis (BE)

A silver wire pseudoreference electrode and gold honeycomb cell card (Pine Research Instrumentation, NC), which contains the working and counter electrodes, were used for bulk electrolysis SEC. The gold working electrode component of the cell card features honeycomb-shaped perforations, which allow the passage of light. An Ocean FX miniature spectrometer and HL-2000-HP-FHSA light source (Ocean Insight, FL) were used to obtain absorption spectra during bulk electrolysis. The absorption spectra were referenced to the solvent. Electrode potential was controlled with an SP-300 potentiostat (Bio-Logic, TN). DPV was performed to determine the potential at the working electrode, and the desired voltage was required to reduce/oxidize cMa at the perforation surface. Chronoamperometry was then performed to determine when equilibration of the sample had occurred, ∼ 2 min after initialization. Light from the halogen lamp passes through the working electrode and is collected and detected by the spectrometer. All electrochemistry was performed in an argon glovebox and 0.1 M TBA(PF_6_) in tetrahydrofuran (THF).

### Pulse Radiolysis (PR)

Pulse radiolysis (PR) was used to measure the molar absorptivities of the oxidized and reduced forms of the cMa photosensitizers studied here, as well as the absorption spectra of the same complexes in their triplet excited states. PR experiments were conducted at the 9 MeV Linear Electron Accelerator Facility (LEAF) at Brookhaven Nation Laboratory (BNL),^27^ using pulses less than 50 ps in duration. The optical detection path consisted of a pulsed xenon arc lamp, a 0.5 cm path length quartz optical cuvette fitted with an airtight Teflon valve, a selectable band-pass interference filter (∼10 nm), and either a silicon (400–1000 nm) or a germanium (1000–1500 nm) photodiode (2–3 ns response time). Optical measurements were collected orthogonal to the direction of the electron pulse through the sample cuvette.

Cations of the cMa complexes were generated by irradiating aerobic *o*-xylene or benzonitrile with high-energy electron radiation to generate solvent cations, solvated electrons, and solvent excited states. The dissolved oxygen readily quenches solvent/solute excited states and solvated electrons, while the solvent cations can be utilized to sensitize the cMa solute. Molar absorptivities were determined via an internal standard method with triphenylamine, whose cation molar absorptivity spectrum is known.

Anions of each complex were generated by irradiating THF with high-energy electron radiation to generate solvent cations and solvated electrons. Solvent excited states are not appreciably generated in THF via pulse radiolysis. The THF cation readily decomposes before it has time to pass charge to a solute, leaving only solvated electrons available for sensitization. The lifetime of the solvated electron is stabilized by the presence of the electrolyte tetrabutylammonium hexafluorophosphate, TBA(PF_6_), in order to ensure diffusion and reduction of the analyte. Molar absorptivities were measured via the internal standard method using biphenyl, whose anion molar absorptivity spectrum is known.

Triplet sensitization experiments were conducted in *o*-xylene degassed with argon, which was irradiated with high-energy electron radiation to generate solvent cations, solvated electrons, and solvent excited states. The excited-state singlets decay many times faster than the rate of diffusion in the solvent, such that only triplet-state solvent molecules are available for sensitization experiments. Since the population of the solvent triplet states is many times larger than the solvent cation or solvated anion, it is assumed that after sensitization of the solute, the resulting spectrum is that of the triplet state of the analyte of interest.

### Sample Preparation for Optical Measurements

The samples were prepared in-house-dry toluene or THF. The concentration was set to have an optical density of 0.1–0.3OD at the pump wavelengths. For the TCSPC and nsTA measurements, an in-house-designed cuvette, ∼1 cm path length, made from borosilicate glass was used, utilizing a Schlenk tap to achieve air exclusion. The samples were bubbled with dry nitrogen for 15 min prior to optical work.

For the psTA experiments, a quartz, 1 mm path length screw cap cuvette was used. First, the cMa compound was prepared in dry solvent (toluene or THF) at the appropriate optical density at 405 nm. This solution was then bubbled with a dry house nitrogen. The deareated solution was quickly transferred to the cuvette and capped with a septum. These solutions were all used within a few hours of preparation as the atmosphere can leak even after a day.

For flow cell experiments, a custom-made 1 cm glass path length cuvette with two 3 mm outer diameter side arm inlet/outlet was used. The inlet side arm was attached via an FEP-lined Tygon tube (ID 1/8″ OD 1/4″) threaded through a 14/20 joint compression clamp thermometer adapter into a 50 mL three-neck round-bottom flask (RBF). The outlet side arm was attached via the FEP line Tygon tubing to a gear pump (Cole-Parmer No. 7144-05), which was also fed back to the three-neck RBF. The third neck of the RBF was fitted with an appropriately sized rubber septa that was used to bubble degas the reservoir *in operando*. The solvent was allowed to circulate through the system for 5 min while being bubble-degassed before spectra were collected. The final reservoir volume accounting for the volume of the lines, cuvette, and pump amounts to ∼100 mL, and the system has a flow rate of 40 mL/min.

### TCSPC

To determine the ISC rates of the cMa complexes, the prompt and delayed emission lifetimes and amplitudes were determined by employing a time-correlated single photon counting technique. The samples were excited by 400 nm laser pulses produced by frequency-doubling the output from a Ti:sapphire regenerative amplifier (Coherent RegA 9050, 800 nm) operating at a 100 kHz repetition rate. The 400 nm excitation pulse was focused on the sample with a focusing lens of 10 cm. Emission was collected with a collection lens kept perpendicular to the excitation beam. For collecting the emission, a Digikröm CM112 double monochromator was used, equipped with a slit width of 0.6 mm and a grating of 1200 lines/mm, achieving a 4.5 nm spectral resolution. The detected emission wavelength varied per compound (492–620 nm). The Hamamatsu R3809U-50 PMT attached at the exit slit of the monochromator operating at 3 kV provided an instrument response of 22 ps. The signals are then amplified and directed toward the Becker and Hickl SPC-630 photon counting board. To avoid pulse pile-up, all of the experiments were performed by keeping the photon counting rate <2% of the repetition rate of the laser.

### psTA

The psTA setup has been described previously^23^ but will be briefly described here. The probe line is fundamentally the same, whereas the pump line is different, so emphasis here will be placed on the pump line. Pump–probe experiments were performed using the output of a Ti:sapphire regenerative amplifier (Coherent Legend Elite, λ = 810 nm, Δλ = 30 nm, 1 kHz, 2.9 mJ, 35 fs), employing a single-wavelength excitation pulse and broad-band probing with a white light supercontinuum pulse. The excitation pulses (λ = 405 nm, Δλ = 6.5 nm) were generated by directing a portion of the amplifier output into a Type I BBO (Red Optronics, 500 μm). The 405 nm is then steered through a synchronous optical chopper (ThorLabs, MC1F10, 10 blades) set at half the laser repetition rate. The beam is then directed through a 25 cm, CaF_2_ focusing lens, which focuses the beam ∼5 cm before the sample. The pump spotsize was 450 μm for most of the samples with ∼2 μJ at target, achieving a fluence of ∼1200 μJ/cm^2^. Differently from the rest of the cMa complexes, Cu_CNCz_^DAC^ was pumped with 700 nJ and a 250 μm spotsize. Sample ground-state absorbance values (0.15–0.25 OD at 405 nm) were maintained experiment to experiment.

The probe pulses were generated as indicated previously. The probe polarization was set at a magic angle (54.7°) with respect to the pump to avoid any contribution to the observed signal from orientational dynamics. After passing through the sample, the generated white light supercontinuum was then collimated and focused on the slit of a Czerny–Turner monochromator. The probe was then dispersed by a diffraction grating (Newport, 500 nm blaze, 150 lines/mm, 2.2° nominal angle) onto a 256-pixel silicon diode array (Hamamatsu) for multiplexed detection of the probe. The 150 lines/mm grating affords an ∼2 nm resolution and was used in all of the psTA experiments except for the Cu_CNCz_^DAC^ experiment, where a 300 lines/mm grating was used that affords twice the spectral resolution (1 nm) but with half the spectral range for the data set. The very large bandwidth covered by the lower-resolution grating is helpful in characterizing a large section of the absorption spectrum but suffers from some distortion of the accuracy of the absorbance magnitude on the near-infrared side of the recovered spectrum. This can be seen when comparing the species-associated spectra with nsTA and psTA data sets (Section S12).

### nsTA

The nsTA experiments were performed on a series of nanosecond–millisecond TA instruments from Magnitude Instruments.^28^ Most experiments were performed on an enVISTA instrument at USC unless otherwise noted. The pump beam was generated from the third harmonic output of a pulsed Nd:YAG laser at 355 nm housed within the overall instrument. The pump laser was set to a 5 kHz repetition rate with routine pulse energies of 40–75 μJ. The pump beam was ∼8 mm Ø, achieving pump fluences of 80–150 μJ/cm^2^. Here, a xenon lamp output acting as the probe was collected and focused into the sample chamber at the sample position. The diverging probe light was recollimated and focused into the monochromator and onto a fast photodiode. The monochromator was equipped with slits of 1.2 mm, affording a 6.3 nm resolution. The monochromator was stepped in increments of 10 nm with a range of 400–900 nm. The oscilloscope voltage resolution was set to 8 bit and a 2 ns step size.

Several of the neat cMa samples and the quenching experiments of Au_BCz_^MAC^ with *N*-methylphthalimide (MePI) in toluene were performed at Magnitude Instruments facility (Pennsylvania State University, State College, PA) with an enVISion instrument where the pump beam was generated with an external 355 nm laser. In addition, it was helpful to record some data with longer pump wavelengths (410, 420, and 450 nm). All such data sets were recorded at the University of California, Riverside in the Bardeen lab with a second enVISion instrument. Here, the pump beam was generated with a 50 Hz repetition rate optical parametric oscillator, tuned to 410 or 420 or 450 nm. The pump beam spotsize was set to ∼8 mm Ø with a pulse energy of ∼1.3 mJ, achieving a pump fluence of ∼2.6 mJ/cm^2^. The higher pump fluence leads to a higher signal at the cost of signal-to-noise due to reduced averaging from the low laser repetition rate for similar experimental acquisition times.

## Results and Discussion

### Ground- and Excited-State Spectra of cMa Complexes

**Figure 2:**
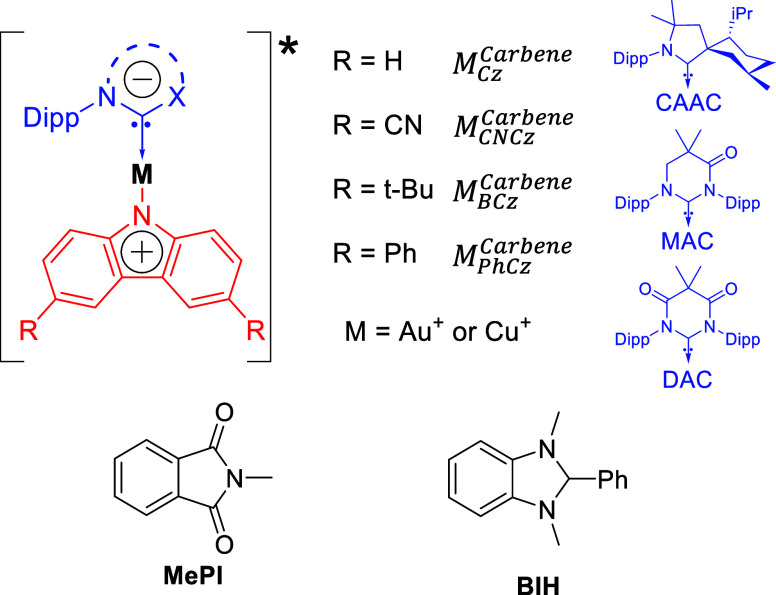
(Left) General structure of the cMa compounds in this article. The cMa on the left is displayed in its excited ICT state. The carbene ligand is displayed in blue, and the amide is displayed in red. (Center) R-group substitutions on carbazole. (Right) Carbene ligands used in this study. (Bottom) *N*-Methylphthalimide (MePI) and dihydrobenzimidazole (BIH) used in electron and hole transfer studies, respectively.

The general structure for the cMa complexes discussed in this study is displayed in Figure 2. The cMa compounds presented here are a class of luminescent, linear, two-coordinate metal complexes composed of a carbene (blue) and a carbazole (red) bonded to a central Cu^+^ or Au^+^ atom.

**Figure 3:**
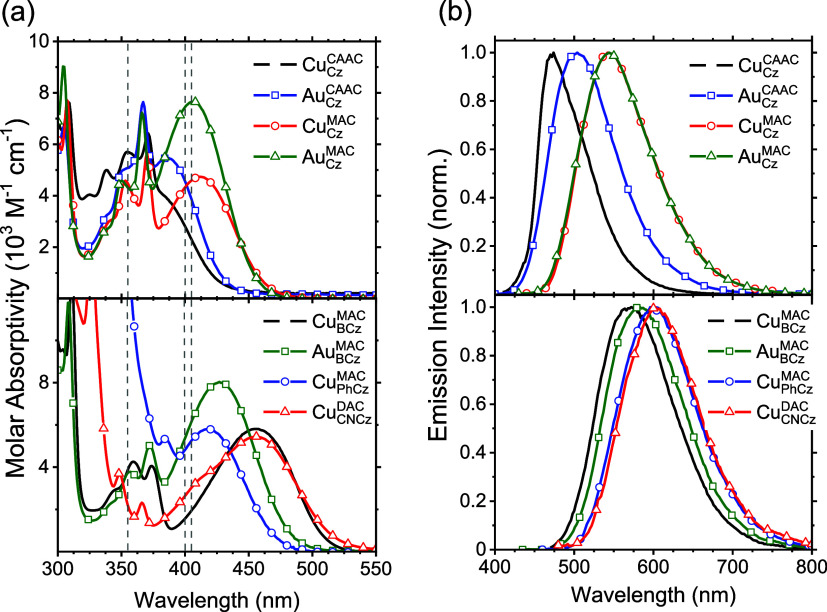
(a) Steady-state molar absorptivity spectra in THF for M_Cz_^CAAC^ and M_Cz_^MAC^ (top) and M_BCz_^MAC^, Cu_PhCz_^MAC^, and Cu_CNCz_^DAC^ (bottom). Cu_CNCz_^DAC^ is in 2-MeTHF. The pump wavelengths used in the laser experiments are indicated as dashed lines: nsTA at 355 nm, TCSPC at 400 nm, and psTA at 405 nm. (b) Steady-state emission spectra of M_Cz_^CAAC^ and M_Cz_^MAC^ in 2-MeTHF (top) and C_BCz_^MAC^, Cu_PhCz_^MAC^, and Cu_BCz_^MAC^ in 2-MeTHF. Au_BCz_^MAC^ is in THF.

The photophysical properties of the cMa complexes studied here have been previously reported.^16−18,24^ The steady-state absorption and emission spectra in THF or 2-MeTHF are displayed in Figure 3. Each complex features structured absorptions from 300 to 380 nm attributed to carbazole,^18^ as well as a relatively strong (ε = 5000–9000 M^–1^ cm^–1^), broad CT transition between 375 and 550 nm. The energy of the ICT band can be easily controlled via careful selection of the carbene and carbazole, with the ICT transition having lower energy with increasing carbene electrophilicity (DAC > MAC > CAAC) as well as increasing carbazole nucleophilicity (Cz > PhCz > BCz ≫ CNCz). The emission spectra all present as structureless bands indicative of ICT states, with λ_max_ values spanning a 220 nm range. The Stokes shifts in THF or 2-MeTHF range from 550 to 900 meV, indicating large excited-state relaxation. The complexes have long-excited-state lifetimes in solution (τ = 0.08–2 μs), with the longest lifetimes in nonpolar solvents and Φ_PL_ values between 0.8 and 0.95.^16−18,24^

### Spectroelectrochemistry

Transient absorption (TA) spectroscopy is a powerful tool for determining the mechanism of excited-state dynamics; however, analysis can prove complicated due to overlapping features. The features of transient absorption can be divided into three categories: ground-state bleach (GSB), stimulated emission (SE), and excited-state absorption (ESA). The GSB and SE can be identified and assigned using the ground-state absorption spectrum and fluorescence/spontaneous emission spectrum, respectively. However, the ESA can prove to be a more difficult feature to assign unless prior spectroscopic knowledge of the excited states formed transiently is available or assumptions are made about these spectra. Recently, McCusker et al. have reported that the TA spectra of the lowest MLCT state of a family of group 8 bis-terpyridine compounds can be approximated as the sum of the cation and anion spectra with the ground-state spectra subtracted.^29^ In this light, we first present a series of pulse radiolysis (PR) experiments to measure triplet excited-state absorption spectra, then spectroelectrochemical measurements, using both bulk electrolysis and PR, of the cation and anion absorption spectra of the cMa compounds. Both the triplet and cation/anion spectra will be used to assign the TA spectra.

**Figure 4:**
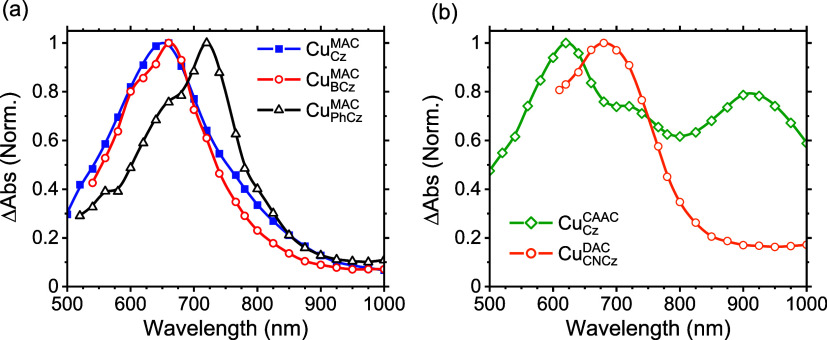
Excited-state absorptivity spectra of (a) Cu_Cz_^MAC^, Cu_BCz_^MAC^, and Cu_PhCz_^MAC^, (b) Cu_Cz_^CAAC^ and Cu_CNCz_^DAC^ in anerobic *o*-xylene 22 ns after electron pulse. All solutions were measured at a concentration of 10 mM, with the exception of Cu_CNCz_^DAC^, which was measured at 2.5 mM due to solubility constraints.

Pulse radiolysis is used to measure the triplet-state absorption spectra of compounds by utilizing high-energy electron radiation to generate solvent triplet states, which are then used to sensitize the analyte.^30^ All five Cu-based compounds’ triplet ESA (Figure 4) were measured in anaerobic *o*-xylene and recorded 22 ns after the electron pulse. For each of the cMa complexes studied here, the excited-state spectra have a peak between 600 and 800 nm. In addition, the excited-state spectrum of Cu_Cz_^CAAC^ displays an NIR absorption located near 950 nm.

**Figure 5:**
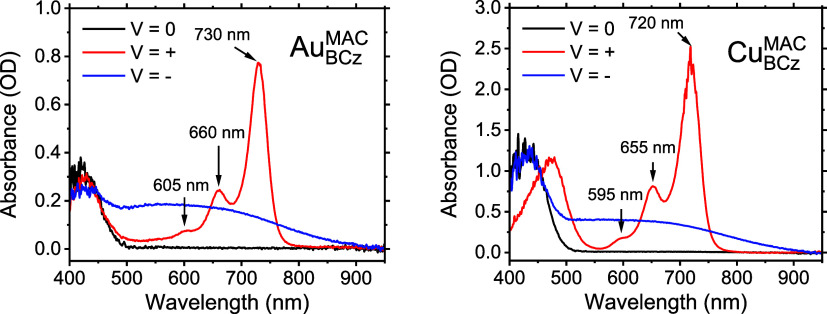
Bulk electrolysis spectra of Cu_BCz_^MAC^ and Au_BCz_^MAC^ in THF. Neutral (0 V)—black, cation (+0.9 V)—red, anion (−2.1 V)—blue. The voltages were referenced to a silver pseudoreference electrode. The peak centers of the cations are also indicated by arrows. A 7-point smooth was applied to the data to remove high noise in the <500 nm region.

For compounds with fully reversible electrochemical oxidation and/or reduction, the absorption spectra of their oxidized (cation) or reduced (anion) forms can be readily obtained by measuring the spectra of the compound in solution as it is oxidized or reduced during bulk electrolysis (BE).^29^Figure 5 displays the BE absorption spectra for Cu_BCz_^MAC^ and Au_BCz_^MAC^ at positive, negative, and neutral applied voltages. We assign the spectra at positive applied voltages to the cation and negative applied voltages to the anion. For Cu_(BCz)^+^_^MAC^, the spectrum shows a vibronically structured absorption with the principal peak maximum occurring at 720 nm. The absorption spectra bear striking similarities to free carbazolium,^16,18,31,32^ which is consistent with oxidation being a carbazole-centered event.^16−18^ For Au_(BCz)^+^_^MAC^, the spectrum displays a 10 nm red shift compared to Cu_(BCz)^+^_^MAC^ but has the same shape. The spectra recorded under reducing conditions for both compounds present as a broad featureless transition from 400 to 900 nm. Due to the time frame of collecting a spectrum by bulk electrolysis (minutes), it is unclear whether these spectra represent the reduced molecular species or the formation of aggregates or colloids at the electrode surface.

**Figure 6:**
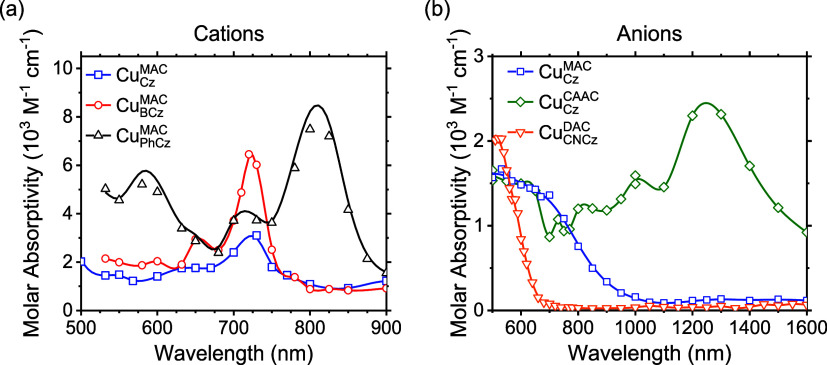
(a) Cation molar absorptivity spectra of 10 mM Cu_Cz_^MAC^ (blue), Cu_BCz_^MAC^ (red), and Cu_PhCz_^MAC^ (black) in a 20 mM solution of triphenylamine in *o*-xylene. (b) Anion molar absorptivity spectra of 10 mM Cu_Cz_^MAC^ (blue), Cu_Cz_^CAAC^ (green), and Cu_CNCz_^DAC^ (orange) in 10 mM TBAPF_6_ in THF. Data collected are displayed as symbols, and the lines are interpolations between data.

For compounds with nonreversible redox events, monitoring the absorption spectra during a BE experiment is problematic due to competing rates of ion degradation. In this case, PR is useful for measuring the spectra of ions before they decay (<10 μs). An additional benefit of PR over BE is that the former gives the molar absorptivity spectra of the charged species. The molar absorptivity spectra of various Cu-based cMa ions are represented in Figures 6 and S1. Irradiating nonpolar solvents generates a large amount of excited solvent molecules and a relatively small number of solvent cations and solvated electrons. However, by keeping the solvent aerated, the excited states and solvated electrons can be quenched out, leaving only the solvent cations to sensitize the analyte. For each of the cMa cations in anaerobic *o*-xylene, the molar absorptivity spectra feature a vibronically structured band, located between 600 and 800 nm in Cu_BCz_^MAC^, Cu_Cz_^MAC^, and Cu_BCz_^MAC^ and between 800 and 1000 nm for Cu_PhCz_^MAC^. Incomplete oxidation in *o*-xylene is observed for Cu_CNCz_^DAC^, likely due to charge delocalization across multiple solvent molecules stabilizing the solvent cation, requiring a switch to benzonitrile. In benzonitrile, Cu_CNCz_^DAC^ presents as a structureless band between 800 and 1000 nm (Figure S2). The shift to a more polar solvent could be the cause for the loss of structured absorption.

In order to measure anion spectra, THF is irradiated with high-energy electrons, yielding solvated electrons and solvent cations. The solvent cations of THF rapidly decompose, leaving only the solvated electrons to sensitize the analyte. Due to reduction of the various Cu cMa compounds being carbene-centered,^16−18^ Cu_Cz_^MAC^, Cu_CNCz_^DAC^, and Cu_Cz_^CAAC^ each possess unique anion molar absorptivity spectra. Cu_Cz_^MAC^ and Cu_CNCz_^DAC^ possess broad featureless absorptions extending from <500 to 1000 nm and from <500 to 700 nm, respectively. Cu_Cz_^CAAC^ displays a large featureless absorption from 600 to 1000 nm, as well as a large, well-defined peak centered at ∼1300 nm. Wavelengths below 500 nm cannot be measured for these compounds due to the presence of the substantial ground-state absorption limiting the detection of the charged species. Since the reduced species are present in a relatively low concentration, being generated uniformly through the cuvette, and spectra are collected in less than 10 μs, the formation of colloids can be excluded. The similarity of the anionic Cu_BCz_^MAC^ spectra generated via BE to the anionic spectra generated via PR suggests that the formation of aggregates or colloids in the BE experiment does not take place. Both the cationic and anionic absorption spectra are useful for providing basis spectra for analyzing intra- and intermolecular charge-transfer events via TA.

### ISC Rates Determined by TCSPC

Previously, we have estimated the rates of ISC for M_Cz_^CAAC^ and M_Cz_^MAC^ compounds, where M = Cu, Ag, and Au, using TCSPC.^17^ Here, we considerably extend these measurements to include a more accurate picture of the ISC process, with different carbazole ligands and solvents, as well as additional carbene ligands. We also use a more complete model than the one used in our previous study, including both the rate of exergonic ISC from *S*_1_ → *T*_1_, *k*_ISC_^exe^, and from endergonic *T*_1_ → *S*_1_, *k*_ISC_^end^. This allows for an estimate of Δ*E*_ST_ for all compounds so far considered.

**Figure 7:**
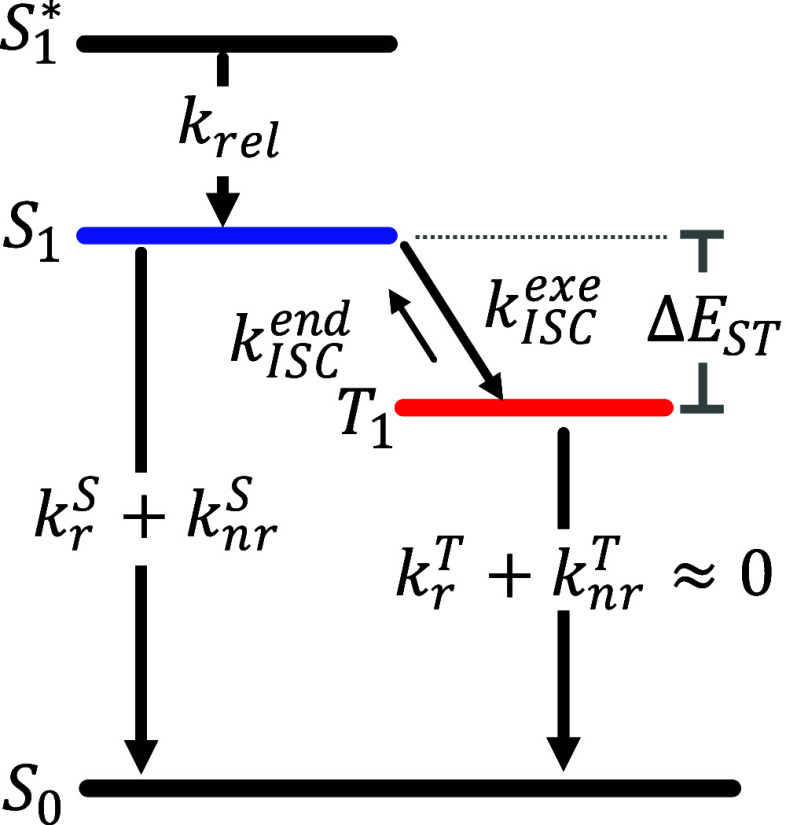
Kinetic model of the cMa compounds. Rates: *k*_rel_—solvent and intramolecular vibrational relaxation to *S*_1_ minimum, *k*_ISC_^exe^—exergonic intersystem crossing from *S*_1_ to *T*_1_, *k*_ISC_^end^—endergonic intersystem crossing from *T*_1_ to *S*_1_, *k*_r_^S^ + *k*_nr_^S^ (or *k*_r_^T^ + *k*_nr_^T^)—the sum of radiative and nonradiative relaxation rates from *S*_1_ (or *T*_1_).

The fluorescence decay curves obtained for the cMa compounds show bimodal fluorescence decay with two distinct decay phases: prompt (picosecond, time constant τ_p_) and delayed fluorescence (decaying up to a microsecond, time constant τ_TADF_). The prompt fluorescence is emission directly from the singlet state before *S*_1_ to *T*_1_ ISC, while the delayed fluorescence is a result of TADF, which results from a rapid equilibration of the *S*_1_ and *T*_1_ excited states before ultimate emission from the singlet state. When time-resolved, the majority percentage of the emission amplitude (*A*_p_) is prompt, while around 1–2% amplitude describes the delayed component (*A*_TADF_ = 1 – *A*_p_); this relative fraction can be captured accurately because of the high dynamic range when photon counting (the delayed fraction has the higher overall integrated area). Previous work simply assumed that the ISC rate could be set exactly to τ_p_^1^. This approach is oversimplified when we consider the thorough TADF kinetics when the singlet–triplet gap, Δ*E*_ST_, is only a few *k*_B_*T*.^17^ To more accurately determine *k*_ISC_^exe^ and *k*_ISC_^end^ in the presence of a fast singlet–triplet equilibrium, we employed the kinetic solution reported by Tsuchiya et al.^33^ The exact equation for the emission decay proposed by Tsuchiya et al. is based on a three-state model, comprised of *S*_1_ and *T*_1_ as excited states and *S*_0_ as ground state (Figure 7). The rate equations for the *S*_1_ and *T*_1_ population can be written as eq 1
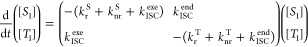
1where *k*_r_^S^ (*k*_r_^T^) and *k*_nr_^S^ (*k*_nr_^T^) are the rates of radiative and nonradiative decays out of the *S*_1_ (*T*_1_). The emission decay directly mirrors the time-dependent population of the *S*_1_ state.^34^ The solution to the above rate equation with the assumption of *k*_r_^T^ = 0 and *k*_nr_^T^ = 0, reasonable based on low-temperature phosphorescence lifetimes determined previously, can be simplified even further.

In most cases considered here, *k*_r_^S^ and *k*_nr_^S^ are small compared to both *k*_ISC_^exe^ and *k*_ISC_^end^, so a simplifying relationship exists to extract both intersystem crossing rates
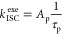
2
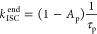
3By taking the ratio of eqs 2 and 3, we can obtain eq 4
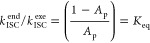
4If we explicitly consider the spectroscopic state degeneracies and assume that the volume and remaining entropy change on a spin flip is negligible, then statistical mechanics tells us that the *T*_1_ ⇌ *S*_1_ equilibrium constant is related to Δ*E*_ST_ via eq 5(35)
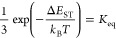
5

If the relative fluorescence component amplitudes can be captured accurately, then Δ*E*_ST_ is accessible to us without temperature-dependent measurements. The emission decay curve was fit to a biexponential decay to obtain the amplitudes and lifetimes of the prompt and delayed fluorescence. The prompt and delayed fluorescence decay constants and amplitudes fitted to TCSPC for Cu_Cz_^MAC^, Cu_BCz_^MAC^, Cu_PhCz_^MAC^, Cu_Cz_^CAAC^, and Cu_CNCz_^DAC^ in both toluene and THF are tabulated in Table 1. For three of the compounds, the approximation employed in eqs 2 and 3 no longer holds; these require the full solution in ref (33) to determine the derived fundamental parameters. Error bars on all quantities are shown with subscripts; all data and fits are shown in Section S2.

**Table 1:** Summary of Measured TCSPC Fluorescence Decays with Derived ISC Rates and Singlet–Triplet Energy Gaps^g^,^h^

*M*_amide_^carbene^	*A*_p_ (%)	*A*_TADF_ (%)	τ_p_ (ps)	τ_TADF_ (μs)	*k*_rel_ (10^12^ s^–1^)	*k*_ISC_^exe^ (10^9^ s^–1^)	*k*_ISC_^end^ (10^9^ s^–1^)	Δ*E*_ST_^a^ (meV)
Cu_Cz_^MAC^	98.69_0.07_	1.31_0.07_	220_8_	1.32_0.02_	0.20_0.2_	4.5_0.2_	0.060_0.005_	83_2_
Cu_BCz_^MAC^	98.56_0.07_	1.44_0.07_	300_10_	0.73_0.02_	0.20_0.04_	3.3_0.1_	0.050_0.004_^b^	81_2_
	98.70_0.05_	1.30_0.05_	350_10_	0.345_0.008_	f	2.8_0.1_	0.040_0.003_^b^	81_2_
Cu_PhCz_^MAC^	98.1_0.1_	1.9_0.1_	280_10_	0.727_0.003_	0.20_0.0_	3.5_0.1_	0.069_0.007_	73_2_
	98.1_0.1_	1.9_0.1_	340_10_	0.45_0.02_	f	2.8_0.3_	0.058_0.005_^b^	73_2_
Cu_Cz_^CAAC^	99.19_0.04_	0.81_0.04_	49_2_	2.0_0.2_	0.2_0.1_	20.2_0.8_	0.162_0.006_	96_2_
Cu_CNCz_^DAC^	98.28_0.09_	1.72_0.09_	127_5_	0.68_0.01_	0.20_0.04_	7.7_0.3_	0.140_0.005_	76_2_
	99.03_0.03_	0.97_0.03_	141_3_	0.048_0.002_	f	7.0_0.2_	0.090_0.04_^b^	84_1_
Au_Cz_^MAC^^c^	99.62_0.03_	0.38_0.03_	8_4_	0.935_0.005_	0.3_0.1_	120_40_	0.5_0.2_	115_18_
Au_BCz_^MAC^^c^	99.52_0.05_	0.48_0.05_	12_1_	0.462_0.004_	0.3_0.1_	83_6_	0.40_0.07_	109_4_
	99.59_0.05_	0.41_0.05_	12_1_	0.248_0.003_	0.8_0.1_	77_6_	0.35_0.06_	113_4_
Au_Cz_^CAAC^^c^	99.45_0.03_	0.55_0.03_	9_3_	1.15_0.05_	0.5_0.1_	110_30_	0.6_0.2_	105_12_
	e	e	11_2_	0.83_0.08_^d^	0.8_0.3_	90_20_	e	e

a Calculated from  (see the text).

b For these compounds, the criterion *A*_p_/τ_TADF_ ≪ (1 – *A*_p_)/τ_p_ no longer holds. We employ the full solution in ref (33) be used to determine *k*_ISC_^exe^ or *k*_ISC_^end^.

c TCSPC instrument-limited τ_p_; ISC rate values and recovered Δ*E*_ST_ from psTA.

d Data in 2-MeTHF are presented in ref (17).

e A TCSPC experiment in THF is unavailable; therefore, these values are undetermined.

f A psTA experiment in THF is unavailable; therefore, these values are undetermined.

g Uncertainty values are displayed in subscript as ±Δ*x*. For example, 220_8_ ps = 220 ± 8 ps. Errors are propagated from confidence intervals on fitted quantities, *A*_p_, τ_p_.

h In single-entry cells, the solvent is toluene. In cells with two values, the top entry is toluene and the bottom entry is THF.

Unlike in our earlier work,^33^ where we estimated the reverse intersystem crossing rate, here we determine it directly from . We now determine *k*_ISC_^end^ ∼ 10^7^–10^8^ s^–1^. Comparable τ_p_ and *A*_p_, at or close to the error limits, in both THF and toluene suggest both *k*_ISC_^exe^ and *k*_ISC_^end^ are to first order independent of the nature of the solvent. The fact that , reflected by the relative amplitude of the long-time fluorescence component, is approximately constant, even though *k*_ISC_^exe^ varies by over an order of magnitude in the compounds studied, reflects that there is a relatively small variation in Δ*E*_ST_, all about 80–90 meV for Cu compounds, a value consistent with temperature-dependent studies reported earlier.^17,24^ Of course, for a given compound, the same spin–orbit coupling constant mediates both forward and reverse processes. On the other hand, we see smaller but now solvent-dependent variations in the τ_TADF_, all close to 1 μs (except Ag cMa compounds),^17^ suggesting that radiative and nonradiative relaxations of the singlet are sensitive, as expected, to different factors than the ISC processes. Looking now at the specific effects of ligand identity, for the series of MAC compounds, the choice of carbazole has the smallest effect on the ISC rates compared to the metal or carbene. Increasing the electron richness of the carbazole by installing peripheral units at the 3- and 6- positions only decreases *k*_ISC_^exe^ by 30% moving from 4.5 × 10^9^ s^–1^ in Cu_Cz_^MAC^ to 3.3 × 10^9^ s^–1^ in Cu_BCz_^MAC^. Changing the carbene has a more substantial effect on ISC. Changing from MAC to the more electron-poor CAAC in the Cu_Cz_ systems, *k*_ISC_^exe^ increases 4-fold from 4.5 × 10^9^ to 20.2 × 10^9^ s^–1^. Cu_CNCz_^DAC^ displays an intermediate value of *k*_ISC_^exe^ of 7.7 × 10^9^ s^–1^, benefiting from an electron-poor carbene, while suffering from an electron-rich carbazole. These results are consistent with our previously reported work, which demonstrated that increasing the amount of metal character, by decreasing the availability of the ligand to contribute to the formed molecular orbitals, results in higher rates of ISC.^17^

While the choice of ligand has an effect on the rates of ISC, the largest contributor to rates of ISC should stem from spin–orbit coupling (SOC), which will change with the prominence of the empty p-orbital on the carbene as well as a function of metal identity.^17^ We can see in the data (Figures S3–S8) the dramatic effect of substituting Au for Cu with the same ligands; all three Au compounds show instrument-limited τ_p_, meaning that the ISC rate is accelerated by at least a factor of 10 with MAC and 2.5 with CAAC. With the instrument limit on τ_p_ of 22 ps, we cannot determine either ISC rate constant directly. To determine the Au cMa ISC rates, we therefore implemented a higher time resolution technique, psTA with an ∼350 fs time resolution applying it to all of the Au and Cu compounds for a consistent treatment. The psTA and TCSPC experiments are fit with the same fast equilibrium kinetic model, with the addition of a solvent relaxation step captured by the psTA experiment but too fast for the TCSPC as will be discussed below. So that they can be combined with the amplitudes in the TCSPC to complete the analysis for Δ*E*_ST_, the psTA-derived *k*_ISC_^exe^ for the gold compounds are included in Table 1.

### Spectrally Resolving ISC with psTA

**Figure 8:**
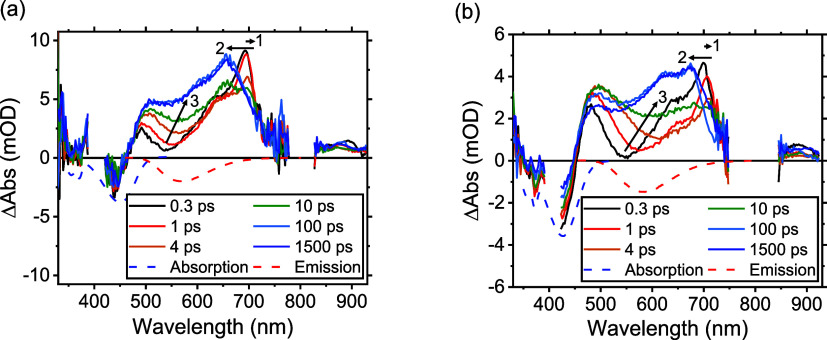
psTA spectra of Au_BCz_^MAC^ in toluene (a) and THF (b) with a 405 nm pump. The temporal evolution is indicated by rainbow color-coded spectral traces. The inverted steady-state absorption (blue dash) and emission spectra (red dash) are displayed. The regions of 400 and 800 nm are removed due to a large pump scatter. The major spectral evolutions are depicted with arrows: initial ultrafast ESA red shift (1), subsequent ESA blue shift (2), and simultaneous SE recovery (3).

With the basis spectral signatures for cMa anion and cation obtained from SEC, we now look more carefully at the spectroscopy and early time dynamics for the cMa compounds using psTA. We present the psTA data for cMa compounds beginning with the example of Au_BCz_^MAC^ in Figure 8. The psTA spectra of Au_BCz_^MAC^ were collected in both toluene and THF with excitation at 405 nm, corresponding to the ICT absorption band of the cMa complexes. The sensitivity in the NIR is discussed in the Methods section, and the full data set (rendered as a contour plot) can be found in the SI (Figures S14–S24). The psTA spectrum has three prominent features. A GSB is assigned based on the good match with the ground-state absorption spectrum from 400 to 450 nm for Au_BCz_^MAC^ (dashed blue). Second, a dip in the ESA spanning ∼500–620 nm is assigned to SE because of a good match in shape with the emission spectrum (dashed red). The final assignment is the overlying ESA spectrum, spanning the entire probe window. The largest and sharpest feature, at earliest delay times (black trace), in the ESA is at 690 nm with a shoulder at 620 nm with a 60% intensity, which bears a strong resemblance to the cation feature in the bulk electrolysis spectrum (Figure 5) but is blue-shifted ∼30 nm. The absorption intensity of the ESA outcompetes the absorption intensity of the SE at all probe wavelengths and delays for Au_BCz_^MAC^ in both solvents.

Having assigned the psTA features, we can analyze the kinetics. Upon light absorption, the photoexcited state formed is a singlet. In toluene, the GSB of Au_BCz_^MAC^ does not shift or lose intensity, indicating that the ground-state population does not recover much over the full 1.5 ns time scale of the measurement. This is expected as this compound has an overall excited-state lifetime of 720 ns in toluene. Over the course of the first 4 ps (black to orange traces), the structured ESA of the singlet state at 690 nm red-shifts by 10 nm (Figure 8, arrow 1). Then, from 5 to 100 ps (orange to blue traces), the initial structured ESA is replaced by a new structured ESA band with ∼2× broader features and with a new center at 650 nm (arrow 2) to the blue of the initial peak. These changes happen on a time scale comparable to the evolution of the SE feature: this is first characterized by the rapid loss of prominence along with a 20–25 nm red shift on the order of 4 ps. After this, the SE band vanishes, almost completely absent at 10 ps, while the ESA reaches its maximum overall intensity at 100 ps (blue trace; arrow 3); subsequent to this, the ESA does not evolve any further. We can now understand that the ESA broadening seen at 100 ps on the bluer side can in part be attributed to the loss of the SE. Recall that the time-resolved fluorescence, reporting just on the singlet population, decays on the same time scale as the loss of the SE and the loss of the sharper ESA and the simultaneous changeover to the blue ESA band (Figure S17). The state formed after SE loss with its major peak at 650 nm is therefore assigned to the triplet and the increase of this band is identified with *S*_1_ → *T*_1_ conversion. Interestingly, aside from the SE and a modest blue shift, the singlet and triplet bands bear a qualitative resemblance to one another.

The psTA experiment in THF qualitatively resembles the situation in toluene. However, the red shift in SE is faster and more extensive over the first 1–4 ps (see also Figure S18); the larger dynamic Stokes shift mirrors the change in steady-state emission spectra (dashed red line) when changing from toluene to polar THF. This earliest phase corresponds to solvent rearrangement around a very different charge distribution, dependent on the solvent polarity. On the other hand, we find by target analysis fitting (see SI Section S6) that the rate of conversion of *S*_1_ to *T*_1_ is the same in both toluene and THF, consistent with the weak dependence on the solvent seen for the copper cMa revealed directly by fluorescence lifetime. We can readily determine the exergonic ISC rate for Au_BCz_^MAC^ in both solvents as *k*_ISC_^exe^ = 80 × 10^9^ s^–1^ (Table 1), a result consistent with hitting the 22 ps instrument limit for the TCSPC measurement.

Previous temperature-dependent TCSPC studies indicate that *k*_ISC_^exe^ ≫ *k*_r_^T^ and *k*_nr_^T^. Depopulation of the triplet is assumed to be solely from *k*_ISC_^end^, and therefore, the emission is approximated to only occur from *S*_1_. This special case is possible due to the small Δ*E*_ST_ where thermal energy allows for intersystem crossing from the longer-lived triplet population pool, giving rise to further delayed emission from the singlet. Due to the small energy gap between the singlet and triplet states, depletion of the singlet occurs but is not complete. While psTA is effective at determining *k*_ISC_^exe^ due to the large contribution of *T*_1_ to the excited-state spectrum, *k*_ISC_^end^ is indeterminate in psTA due this technique’s more limited dynamic range and the only ∼1–2% equilibrium *S*_1_ population. Complementarily, TCSPC only reports on the singlet population, but with a high dynamic range, but is blind to the triplet population, which is fully revealed in the psTA.

Therefore, if possible, values of *k*_ISC_^exe^ and *k*_ISC_^end^ were taken from the TCSPC measurements and treated as fixed. Because the fastest decay for the gold complexes is below the time response of the TCSPC apparatus, the values for *k*_ISC_^exe^ for these complexes were fit from psTA data such that τ_p_ = (*k*_ISC_^exe^)^−1^ and this parameter was fixed. Then, from a fit to the TCSPC data of the gold compounds that includes convolution with the measured IRF, the *A*_p_ and (1 – *A*_p_) were extracted, allowing *k*_ISC_^end^ and Δ*E*_ST_ to be subsequently obtained. For all of the Au-containing cMa compounds, this approach is used to determine *k*_ISC_^exe^ and estimate *k*_ISC_^end^ and thus Δ*E*_ST_ to complete Table 1. Through this analysis, we find that Au_BCz_^MAC^ has Δ*E*_ST_ ∼ 30–40% higher than the analogous copper complex, Cu_BCz_^MAC^.

**Figure 9:**
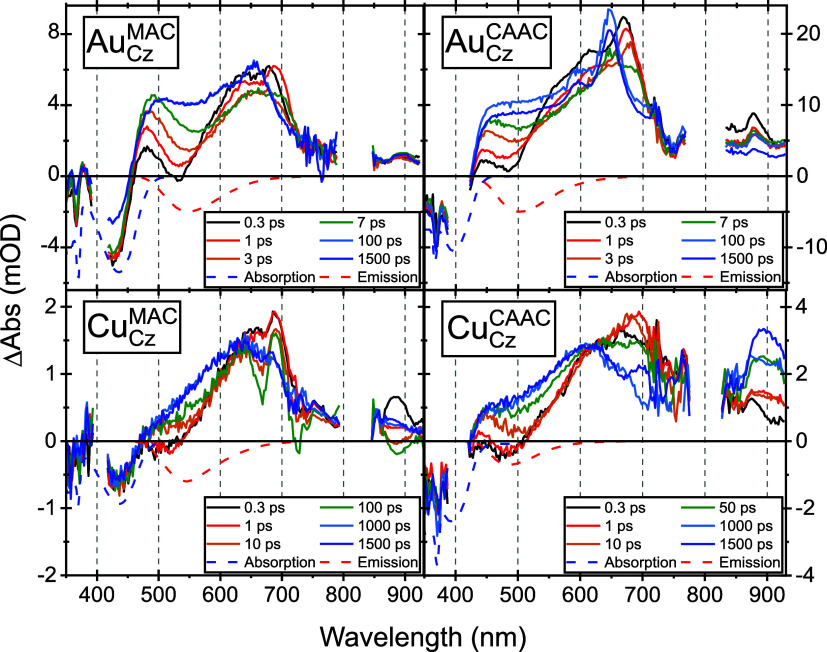
psTA spectra of *M*_Cz_ class of cMa compounds with excitation at 405 nm in toluene with Cz kept constant as the amide ligand. The gold and copper complexes are the top and bottom rows, respectively. *M*^MAC^ and *M*^CAAC^ compounds are the left and right columns, respectively.

We are now ready to move on to the psTA spectroscopy when substituting Cu for Au and explore the CAAC carbene ligand for both metals (Figure 9) and cMa compounds where the carbazole ligand is substituted (Section S3). For all four M_Cz_^MAC^ and M_Cz_^CAAC^ data sets in toluene (Figure 9), there are strong qualitative similarities to the discussion above for Au_BCz_^MAC^ and all data sets can be fit with the same model. Namely, for each data set, there is a distinct negative GSB at the blue end, with the remaining spectrum dominated by a sharper absorption in the region of 600–725 nm for the ESA overlapped by a time-evolving SE band to its blue side, which captures first the ultrafast solvation of the singlet and then its demise with a parallel time evolution for the red end of the ESA. Additional analysis of the spectral changes follows in the next section. However, because of the broad similarities in kinetics and spectral detail, the spectral assignments for Au_BCz_^MAC^ are applied to the remaining cMa compounds. A quantitative analysis is applied to the full two-dimensional (2D) spectral–temporal data set of each compound using a target analysis with the kinetic model described in the previous TCSPC section and Figure 7. Fitting details are provided in Section S6. The same set of parameters is used simultaneously to fit psTA data and TCSPC for all compounds, supporting the assignment of the ISC rate constants. A full summary of kinetic parameters resulting from this target analysis fitting for all cMa compounds measured is organized in Table 1.

To briefly summarize an extensive data set, the initial singlet population experiences solvent configurational relaxation labeled as *k*_rel_, where *k*_rel_ ∼ (0.2–0.8) × 10^12^ s^–1^ for all cMa compounds. Meech et al.^36^ have published a recent study of Au_Cz_^CAAC^ in a more polar solvent, chlorobenzene. They assign the early time to solvation; however, there are also contributions from vibrational relaxation determined from femtosecond stimulated Raman spectroscopy. Fitting reveals that *k*_rel_ in THF is a factor of 2 faster than in toluene but independent of carbene and amide identity. After initial stabilization, the cMa exists in an *S*_1_ ⇄ *T*_1_ dynamic equilibrium for the lifetime of the excited state. We confirm the large impact on the spin–orbit coupling (SOC) effect with a change in metal; psTA studies reveal that a gold atom leads to an ∼25× fold increase in *k*_ISC_^exe^ due to its higher SOC strength compared to copper. The psTA experiments allow us to reveal for gold the same behavior already noted for copper. Namely, for Au_BCz_^MAC^ and Au_Cz_^CAAC^, k_ISC_^exe^ is moderately independent of solvent or the identity of the amide, with Au_Cz_^MAC^ experiencing an ∼1.2 increase compared to the other gold compounds. And our analysis suggests that all of the gold-containing complexes have somewhat higher Δ*E*_ST_ than the copper complexes, consistent with theoretical calculations for the gap by Muniz et al.^24^

Considering the spectral variation exhibited in Figure 9, the largest peaks at 700 nm in the singlet and 650 nm in the triplet for Au_Cz_^CAAC^ are sharper than for Cu_Cz_^CAAC^. The valley at early times carved out by the stimulated emission in the gold cMa complexes is also further enhanced, evidenced by the shoulder at 470 nm in Au_Cz_^MAC^ and Au_Cz_^CAAC^ increasing sharper than Cu_Cz_^MAC^ and Cu_Cz_^CAAC^.

### Excited-State Spectral Recreation

**Figure 10:**
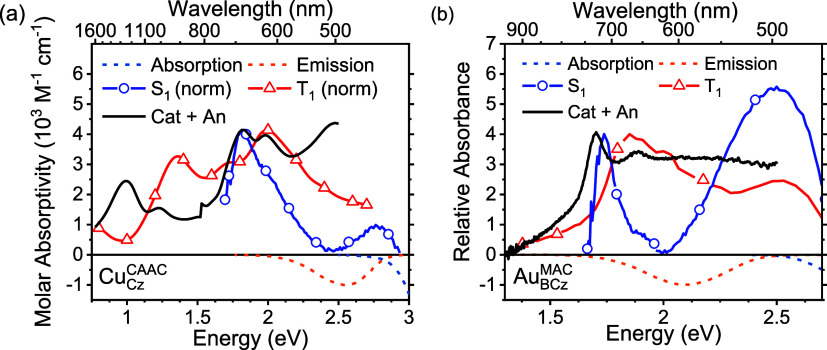
Simulation of the ESA by the summation of cation and anion basis spectra (black) with comparisons made to spectral representative of the singlet (blue) and triplet (red). (a) Cu_Cz_^CAAC^ using PR data for cation plus anion from Figure 6 and triplet from Figure 4b. Singlet comes from psTA species-associated decay spectra (SADS) recorded in toluene. (b) Au_BCz_^MAC^ using BE data for cation plus anion, singlet ESA recovered from psTA SADS, and triplet ESA from nsTA SADS. The black, blue, and red curves have been scaled to match in the region between 1.6 and 2 eV. All data for Au_BCz_^MAC^ were recorded in THF. The absorption (dashed teal) and emission spectra (dashed orange) are normalized and displayed as inverted spectra.

Recently, McCusker et al. published a report wherein the ESA of group 8 octahedral metal tris-bipyridyl complexes when excited into MLCT states were shown to be well approximated as a sum of the cation and anion spectra for the complex.^29^ It is useful to analyze the character of the ligand-to-ligand ICT excited state formed here and, in particular, compare the situation when the promoted electron is localized on a single unit, as it is the cMa ICT state, rather than being delocalized over the entire octahedral ligand sphere as in the tris-bipyridyl complexes. We can also make this comparison for the spectral signatures of both triplet and singlet ICT states. In Figure 10, we compare the excited-state spectra (originating from both singlet and triplet *M*_(amide)^+^_^(carbene)^−^^) for several of the complexes to the sums of *M*_(amide)^+^_^carbene^ and *M*_amide_^(carbene)^−^^ spectra measured from SEC (see also Section S13).^29^ The two features present in the simulated Cu_Cz_^CAAC^ (Figure 10a) spectrum at ∼1 and ∼1.8 eV, arising from *M*_amide_^(carbene)^−^^ and *M*_(amide)^+^_^carbene^, respectively (from PR and BE SEC), also appear in the measured *T*_1_ spectra for this compound, but a constant 0.3 eV blue shift needs to be applied to align the peak positions. The higher-energy (cation) feature of the simulated spectra is also present in the recovered *S*_1_ ESA (lower energies are not recorded in psTA); however, it is better energy-aligned without the need for a blue shift. Similarly, the simulated spectra for the rest of the copper cMa complexes (Figures S55 and S56) are largely comprised of the carbazolium feature between 1.5 and 1.7 eV and match well to the respective *S*_1_ and *T*_1_ ESA, which are dominated by a feature between 1.6 and 1.9 eV. Despite the choice of carbazole varying the oxidation potential of the cMa widely, its appearance in the ESA is qualitatively similar. In general, the width of this feature for the singlet and the cation/anion composite is narrower and the triplet somewhat broader. In the case of Cu_Cz_^CAAC^ and Cu_Cz_^MAC^, the relative intensity of the absorption peaks and the peak vibronic structure seem well matched between the simulated and real spectra, while this is not the case for the other compounds.

The ESA spectra of Au_BCz_^MAC^ can also be reconstructed by summing the cation and anion but this time from BE spectra (Figure 10b). The singlet and triplet are represented by the species-associated decay spectra (SADS) obtained from fitting the psTA and nsTA data, respectively. Because BE data are used, spectra on an absolute molar absorptivity scale are not available; the spectral sum is terminated >2.5 eV due to spectral uncertainty from saturation for unoxidized (unreduced) Au_BCz_^MAC^ in the cation (anion) spectra. The comparison is similar to the copper complexes, with most features in the two ESA spectra matching the anion/cation sum and with a larger shift required for the triplet. The (inverted) steady-state emission and absorption spectra are included to rationalize the deviations at higher transition energy; these arise from ground-state bleaching (seen in both T_1_ and S_1_ TA spectra) and stimulated emission (for the S_1_).

The most obvious feature in common from these two different analyses is the transition energy mismatch when comparing the reconstructed anion/cation-summed spectra and the triplet. With the exception of Cu_Cz_^MAC^ (Figure S55, left) where there seems to be good alignment, the peaks originating from both the anion and cation components of the Cu_Cz_^CAAC^ simulated spectra are ∼300 meV lower in energy than their *T*_1_ ESA counterparts, while the carbazolium features in the ESA of the MAC family are somewhere between 0 and 200 meV. The need for an energy shift is not observed in the treatment reported by McCusker et al. for octahedral, tris-bisimine metal complexes.^29^ This energy mismatch could potentially be due to a larger Coulombic interaction between localized charges in the linear cMa ICT state or different interactions in the higher-lying *S*_*n*_ and *T*_*n*_ states to which the ESA transitions originating in the *S*_1_ and *T*_1_ states connect.

Here, we have shown that both SEC techniques provide the ability to deconstruct and analyze the ICT excited-state spectra. PR is particularly useful as it allows the determination of absolute molar absorptivities of radical cations and anions. With a full understanding of the intramolecular spectra and dynamics, we shift focus to intermolecular charge-transfer dynamics of the cMa with an electron acceptor or donor.

### Observing Intermolecular CT via nsTA

The psTA experiments provide a detailed picture of the intramolecular charge-transfer dynamics, but the 1.5 ns time limitation of the psTA limits our ability to monitor diffusion-controlled intermolecular charge-transfer reactions. Diffusion of the photosensitizer and electron acceptor or donor close enough for charge transfer to take place is on the order of nanoseconds for moderate concentrations (1–50 mM). To demonstrate this, psTA was used to follow the reaction of Cu_BCz_^MAC^ with the oxidizing agent MePI to form Cu_(BCz)^+^_^MAC^ in toluene. However, the psTA instrumental time range limitation of ∼1 ns captures only the onset of the intermolecular electron transfer in this case, complicated by concomitant ISC (Figure S12). Therefore, we have used nsTA, pumped in the majority of cases by a 355 nm nanosecond laser at USC, to capture long-time diffusive charge transfer from cMas to both an electron and hole acceptor.

**Figure 11:**
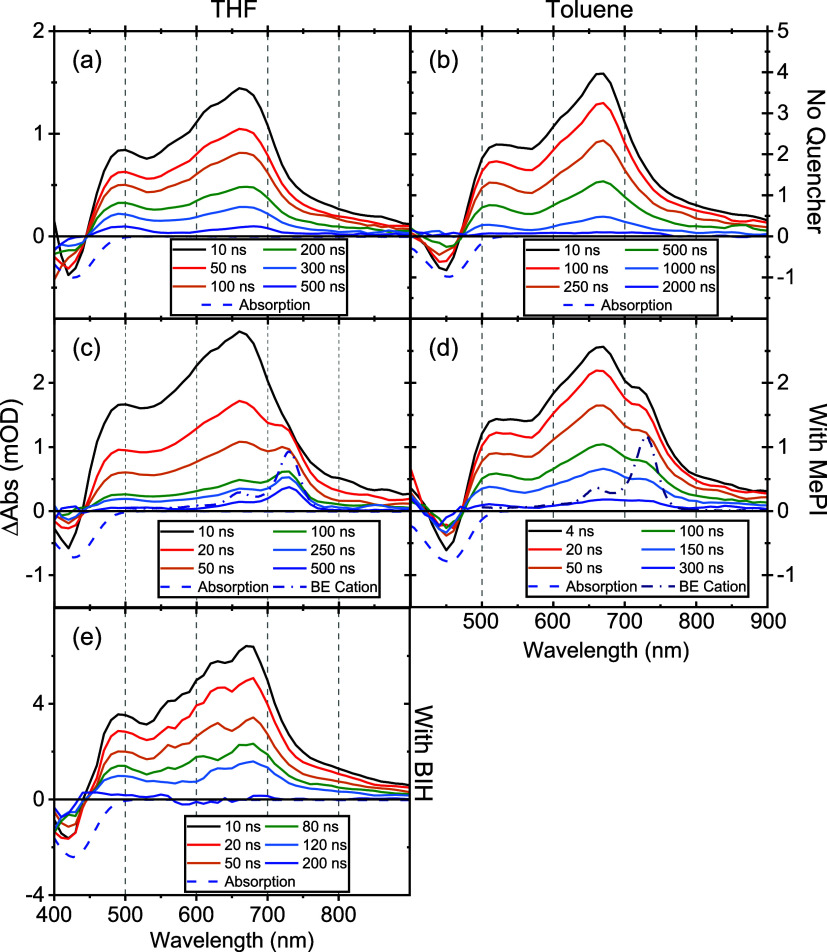
Au_BCz_^MAC^ nsTA spectra as neat solutions (top row), with MePI as an electron acceptor (middle row), and BIH as a hole acceptor (bottom row) in THF (left column) and toluene (right column). All data were pumped with 355 nm except for panel (e), which was pumped with 450 nm. The concentrations of the electron or hole acceptors are as follows: in panel (c), [MePI] = 6 mM; in panel (d), [MePI] = 280 mM, in panel (e), [BIH] = 100 mM. Data in panels (c) and (e) were collected in 100 mL flow cells.

nsTA data sets for all cMa complexes, both in THF and toluene, are shown in Figures 11a,b and S32–S39. For the copper complexes, the nsTA data display no noticeable spectral changes from 10 ns to 2 μs when the traces return to baseline. However, during the experimental run time, we find that in the THF solution, both Cu_Cz_^CAAC^ and Cu_CNCz_^DAC^ decompose (this can be seen most clearly for Cu_CNCz_^DAC^ in Figure S37, left), while Cu_PhCz_^MAC^ decomposes on irradiation in both solvents. Cu_Cz_^MAC^ and Cu_BCz_^MAC^ performed relatively well. Importantly, degradation is not observed when any of these Cu complexes are excited with either the 400 or 405 nm sources described earlier, nor during the collection of photophysical data reported previously,^16−18^ nor with photoreactor studies of Cu_PhCz_^MAC^ where the complex is irradiated with 460 nm LEDs for 90 min (Figure S59b). We therefore do not suspect any inherent instability in the *S*_1_ and *T*_1_ ICT states, but rather it is the use of 355 nm that is causing the problem. This excitation wavelength leads to excitation to an MLCT, which will lead to the Renner–Teller distortion,^37^ followed by solvation and then ligand loss before possible relaxation to the lower-lying ICT state. While we are currently limited to 355 nm laser as our excitation source at USC, future work will substitute direct excitation into the ICT state for these nsTA studies. We stress again that this limitation does not preclude these cMa complexes used as visible light photosensitizers.

In order to study charge-transfer dynamics from *M*_(amide)^+^_^(carbene)^−^^ to an electron acceptor or donor, the excited states of various cMa complexes were used to reduce *N*-methylphthalimide, MePI, or oxidize N-dimethyl-dihydrophenylbenzimidazole, BIH. To avoid the UV photodegradation just described for copper complexes, we chose to test gold-based cMa compounds, first Au_Cz_^MAC^ and then Au_BCz_^MAC^; in the absence of a quencher we find both to be photostable to 355 nm excitation. The addition of 7 mM MePI to 46 μM Au_Cz_^MAC^ in THF results in a decreased lifetime of 240 ns, which could be attributed to the transfer of either charge or energy to MePI. However, despite the decreased lifetime, no new features are observed in the spectra (Figure S40). Recording the ground-state absorption of the sample cuvette pre- and postexperiment reveals a large amount of sample degradation after quenching with MePI. This degradation is unsurprising as the cyclic voltammogram (CV) trace reveals a nonreversible oxidation wave for the compound,^18^ leading to a lifetime too short for the cation to recombine with MePI^–^ to form the stable neutral state. As the oxidative nonreversibility in Au_Cz_^MAC^ is attributed to the polymerization of the unsubstituted 3- and 6- positions in the carbazole ligand, consistent with irreversible oxidation of bare Cz,^18^ our approach has been to make substitutions at these locations with *tert*-butyl groups in Au_BCz_^MAC^ to impart reversibility, which is observed in its CV.^24,38^

This modification is clearly observed to rectify the problem for the photoinduced charge-transfer dynamics. Upon the addition of 30 mM MePI to 75 μM Au_BCz_^MAC^ in THF, a new feature matching the cation peak is clearly seen with an increase of ∼50 ns at ∼720 nm (Figure S41), which persists for ∼110 μs (Figure S42). As the PL is completely extinguished with a similar 50 ns lifetime, this indicates charge transfer with no recombination to the excited state. The 110 μs lifetime is more than sufficient for a sacrificial reductant to regenerate the neutral Au_BCz_^MAC^. Reducing the concentration of MePI to 6 mM in THF allows us to clearly observe the signal from the triplet alone at early time slices, which slowly evolves into the cation spectra over ∼250 ns. This more illustrative case is shown in Figure 11c; the spectra recovered are invariant to excitation wavelength. The quenching rate constant, *k*_q_, of Au_BCz_^MAC^ with MePI in THF is (1.0 ± 0.1) × 10^9^ M^–1^ s^–1^, which is in reasonably close agreement with that obtained from the previous Stern–Volmer quenching analysis.^24^ By checking for degradation comparing UV–vis spectra recorded before and after the experiment, we find that the use of a flow cell eliminates the degree of photodegradation when excited at 355 nm.

Hole transfer from excited Au_BCz_^MAC^ to a sacrificial donor (BIH) in THF was then investigated. A quenching rate constant, *k*_q_, of (2.6 ± 0.4) × 10^8^ M^–1^ s^–1^ is first established from a new Stern–Volmer experiment using TCSPC and excitation at 405 nm (Figure S60). Because BIH has its own absorption shorter than 400 nm (Figure S62), it was necessary to carry out nsTA experiments on BIH quenching of Au_BCz_^MAC^ on a similar instrument at UC Riverside capable of exciting at 450 nm where the BIH absorbance becomes negligible at the [BIH] used. A nsTA experiment of Au_BCz_^MAC^ with 100 mM BIH in THF pumped results in excited-state quenching and reduced lifetime (Figures 11e and S61, 240–28 ns; see also Figure S35 for neat Au_BCz_^MAC^ pumped at a longer wavelength). Indeed, the quenching of the Au_(BCz)^+^_^(MAC)^−^^ triplet is consistent with the ∼30 ns quenching time constant predicted using the SV quenching rate constant at a BIH concentration of 100 mM. However, the expected product(s) of the quenching reaction (Au_BCz_^(MAC)^−^^, BIH^+^, or ^3^BIH) are not observed at even the longest time delay nsTA slices, despite the reduction in lifetime. We note that regardless of excitation wavelength, nsTA experiments with ∼1 mM Au_BCz_^MAC^ and 100 mM BIH in THF show no degradation of the cMa over the course of the nsTA experiment, indicating that the product of Au_(BCz)^+^_^(MAC)^−^^ quenching by BIH to be stable, consistent with the reversibility in cyclic voltammetry experiments.^24^

This leaves an open question about the role of BIH in the photoelectrochemical studies reported recently, where Au_BCz_^MAC^, BIH, a cobalt hydrogen evolving reaction (HER) catalyst, and water were irradiated at 470 nm and produced substantial amounts of hydrogen.^24^ If BIH is left out of the system, no hydrogen is produced, so clearly the BIH is acting as a reducing agent in some capacity. It is important to note that the HER experiments were done with high-intensity irradiation over many hours. The turnover numbers were good, up to 35 equiv of hydrogen per cMa sensitizer, but the quantum yield of hydrogen based on photons absorbed by the sensitizer is quite low. The nsTA experiments show the cMa triplet to be efficiently quenched, but as we do not see evidence for appreciable concentrations of the reduced cMa from hole transfer to BIH, however, the charge-transfer pathway may be a minor one relative to triplet energy transfer to BIH. If the electron transfer pathway were a few percent or less, it would not be detectable in the nsTA experiments but certainly could have given the level of hydrogen. Perhaps, the signal arising from ^3^BIH is masked by the Au_(BCz)^+^_^(MAC)^−^^ triplet, but this would require the triplet–triplet absorption spectrum of ^3^BIH to be very weak—and below the sensitivity limit of the instrument at the longest delay times when the triplet has totally disappeared. The hydrogen production seen in the HER experiments suggests that a sacrificial reductant with a triplet energy higher than that of the sensitizer could give substantially higher hydrogen yields in this system.

Electron transfer from Au_BCz_^MAC^ to MePI was reexamined in toluene (Figure 11d). Using 280 mM MePI, results in the formation of the cation at the earliest experimental time delays resolvable. Throughout the course of the experiment, the ratio between the triplet and cation peaks remains unchanged and all kinetic traces have a lifetime of 120 ns, less than the lifetime of the complex alone. An equilibrium constant of *K* ∼ 0.4 was discovered, favoring the triplet population over cation formation (Section S9). Photoluminescence is also detected until the TA spectra return to baseline. This result suggests that while an electron is transferred from Au_BCz_^MAC^ to MePI in toluene, as expected, the ion pair of Au_(BCz)^+^_^MAC^ and MePI^–^ is not able to escape from the nonpolar solvent cage. Inside the cage, the charges then collide many times, recombining to either the ground or excited state of Au_BCz_^MAC^. Attempts to repeat this experiment at a lower concentration of 6 mM MePI resulted in no cation peaks being observed, mostly likely attributed to the increased driving force required to transfer charge in nonpolar solvents.^5^

## Conclusions

The cMa class of complexes presented here are strong candidates as photosensitizers in the production of energy-dense solar fuels from a common feedstock such as hydrogen from water due to their strong, tunable absorptions, long-excited-state lifetimes, large excited-state redox potentials, and good stability in their reduced and oxidized forms (with appropriate ligand substitution). The long lifetimes have been achieved via a small Δ*E*_ST_, allowing for rapid ISC into the *T*_1_ state even without the use of a heavy atom, by invoking a TADF regime. By applying a model laid out by Tsuchiya et al.,^33^ values of *k*_ISC_^exe^, *k*_ISC_^end^, and Δ*E*_ST_ have been determined through fluorescence lifetimes measured by TCSPC, without the need to rely on a thermal fitting procedure as done in our prior report.^17^

Fluorescence lifetime measurements indicated that the strongest change in the ISC rates occurs when changing the metal from Cu to Au, consistent with higher SOC and leading to an increase in ISC by ∼25 times. The identity of the carbene and amide ligands also plays a role in moving between the two states, affecting the *k*_ISC_^exe^ by a factor ∼4.5 and ∼1.3, respectively, for the choices of carbene and amide ligands explored here. We attribute the larger effect of carbene changes on the ISC rate to the carbene ligand having a greater effect on the SOC from the metal ion than the carbazole. The copper-based cMa complexes are able to achieve fast rates of ISC and long lifetimes while utilizing an abundant first-row transition metal, making them promising for low-cost sensitizers for photoelectrocatalytic processes, such as the production of solar fuels.

The excited-state dynamics of the complexes were further probed by psTA, which revealed a further time regime: a fast component (∼1.5–5 ps) contributed to solvent relaxation and vibrational relaxation of the excited state. The spectral evolution of *S*_1_ to *T*_1_ ISC was revealed with dynamics mirroring fluorescence lifetime as well as elucidating ISC rates for the gold atom complexes otherwise beyond the instrument limit of TCSPC. The psTA triplet spectra match well to the long-time spectra obtained by PR. By summing together the molar absorption spectra of the cation and anion from PR, the ESA spectrum of the *S*_1_ and *T*_1_ states of Cu_Cz_^CAAC^ can be approximated with good matching of the relative intensities of the transition but with an energy discrepancy between the simulated and real ESA of 0–400 meV. We recognize that access to PR facilities is limited; however, BE exists as an equivalently viable technique. As such, the ESA of Au_BCz_^MAC^ was also simulated by summing the spectra for the cationic and anionic absorptivity spectra collected via BE, which again was in good agreement with the real ESA.

In the ns regime, TA studies of Au_BCz_^MAC^ were used to spectroscopically capture the transfer of either a hole to BIH or an electron to MePI in THF. In the case of electron transfer to an acceptor, quenching produces the cation of the Au_BCz_^MAC^ with a diffusion-limited charge-transfer rate, with a spectral signature in good agreement with the ionic spectra collected via SEC. The cation of Au_BCz_^MAC^ possesses a 110 μs lifetime before either charge recombination or degradation. This lifetime is more than sufficient for reduction by a separate source to recover the ground state for the photocatalytic cycle to continue. Attempts to observe hole transfer to BIH from Au_BCz_^MAC^, however, were dominated by a signal arising to a much more efficient energy-transfer process, suggesting that while electron transfer occurs, it is a minor event.

Moving from THF to toluene results in an equilibrium seen in the earliest time slices, between Au_(BCz)^+^_^(MAC)^−^^ and the solvent-caged Au_(BCz)^+^_^MAC^ and MePI^–^. The same equilibrium behavior is seen for Cu_BCz_^MAC^. Such a cage mimics covalently linking the PS to an EC, removing the kinetic rate limit stemming from the two diffusing together during the production of solar fuels.^39^ By using an inert covalent linker, this intermolecular charge separate ion pair can be rapidly realized, increasing catalytic rates. While degradation was observed in nsTA experiments for some of the other copper cMa complexes using a high-energy excitation source (355 nm), no such degradation was observed when the same molecules were irradiated by lower-energy sources at 460 nm LEDs, indicating that the PS family presented here will be stable to illumination by sunlight. Recent work from our group has shown that both gold- and copper-based cMa complexes can act as efficient sensitizers in the photoelectrocatalytic water reduction, i.e., the hydrogen evolving reaction (HER).^24^ Finally, work is underway to explore covalently linking a cMa to a desired electrocatalyst to remove the need for long-time diffusion, thereby facilitating the photocatalytic cycle.

## Data Availability

The data underlying this study are openly available in the Zenodo repository at doi:10.5281/zenodo.10901021.

## Supporting Information Available

Molar absorptivity spectra of cMa ions; time-correlated photon counting decay curves; transient absorption data (psTA heat maps and spectra, nsTA spectra), target analysis TA fitting, and species-associated decay spectra from TA; simulated excited-state absorption spectra from ionic spectra; PL correction information for nsTA; and photostability measurements of photosensitizers (PDF)
