# Correction to “Intra- and Inter-Molecular Charge Transfer Dynamics of Carbene–Metal–Amide Photosensitizers”

**DOI:** 10.1021/acs.jpcc.4c03368

**Published:** 2024-06-10

**Authors:** Michael S. Kellogg, Austin R. Mencke, Collin N. Muniz, Thabassum A. Nattikallungal, Fabiola Cardoso-Delgado, Nina Baluyot-Reyes, Marielle Sewell, Matthew J. Bird, Simran S. Saund, Nathan R. Neale, Stephen E. Bradforth, Mark E. Thompson

**Affiliations:** † Department of Chemistry, University of Southern California, Los Angeles, California 90089, United States; ‡ Chemistry Division, Brookhaven National Laboratory, Upton, New York 11973, United States; § Department of Chemistry, University of California, Riverside, California 92521, United States; ∥ Chemistry & Nanoscience Center, National Renewable Energy Laboratory, Golden, Colorado 80401, United States

We would like to correct our paper by adding two authors (S. S. Saund and N. R. Neale) and by expanding the acknowledgment section of the paper. Drs. Saund and Neale were instrumental in the collection of the spectroelectrochemical data based on bulk electrolysis, illustrated in Figure 5 of the manuscript. This data was vital in assigning signatures of oxidized and reduced cMa complexes in the transient absorbance spectra. The changes are reflected in the authorship of this Correction.

