# Supplementary material for: Intra- and Intermolecular Charge-Transfer Dynamics of Carbene–Metal–Amide Photosensitizers
Source: J Phys Chem C Nanomater Interfaces. 2024 Apr 12;128(16):6621–35. doi: 10.1021/acs.jpcc.4c01994 (PMC11056983; doi:10.1021/acs.jpcc.4c01994)
Supplement: Supplementary file 1 — jp4c01994_si_001.pdf [file jp4c01994_si_001.pdf]

Supporting information

Intra- and Inter-Molecular Charge Transfer Dynamics of Carbene-Metal-Amide Photosensitizers

Michael S. Kellogg[1‡], Austin R. Mencke[1‡], Collin N. Muniz[1], Thabassum A. Nattikallungal[1], Fabiola Cardoso-Delgado[1], Nina Baluyot-Reyes[1], Marielle Sewell[3], Matthew J. Bird,[2] Stephen E. Bradforth,[1,*] Mark E. Thompson[1,*]

1. Department of Chemistry, University of Southern California, Los Angeles, CA 90089
2. Chemistry Division, Brookhaven National Laboratory, Upton, NY 11973
3. Department of Chemistry, University of California, Riverside, CA 92521

[‡] These authors contributed equally

Corresponding authors: stephen.bradforth@usc.edu, met@usc.edu

Table of Contents

Section S1. Absorptivity Spectra of cMa ions from Pulse Radiolysis 5

Figure S1 – Cation molar absorptivity spectra of 10 mM CuCzCAAC in 20 mM solution of triphenylamine in o-xylene. The circles are data points and the line is the interpolation between the symbols. 5

Figure S2 – Cation absorptivity of 10 mM CuCNCzDAC in benzonitirile. The circles are data points and the line is the interpolation between the symbols. 5

Section S2. Time Correlated Single Photon Counting Decay Curves 6

Figure S3 – Emission decay trace for CuCzMAC (left) and AuCzMAC (right) in toluene.......................................6

Figure S4 – Emission decay traces for CuBCzMAC in toluene (left) and in THF (right). 6

Figure S5 – Emission decay traces for AuBCzMAC in toluene (left) and in THF (right). 7

Figure S6 – Emission decay traces for CuPhCzMAC in toluene (left) and in THF (right). 7

Figure S7 – Emission decay traces for CuCNCzDAC in toluene (left) and in THF (right).......................................7

Figure S8 - Emission decay trace for CuCzCAAC (left) and AuCzCAAC (right) in toluene. 8

Section S3. Picosecond Transient Absorption Data on changing the carbazole unit and solvent.. 9

Figure S9 – psTA spectra of CuBCzMAC (left) and CuPhCzMAC (right) in toluene...10

Figure S10 – psTA spectra of CuCNCzDAC in toluene. 10

Figure S11 – The psTA spectrum of AuCzCAAC in THF...11

Section S4. Charge transfer within a 1 ns window with concentrated quencher................. 12

Figure S12 – The psTA spectrum of CuBCzMAC in toluene with 280 mM MePI. 12

Figure S13 – Kinetic model used for fitting the quenching of CuBCzMAC with MePI. The left-hand side is the same model as the psTA fitting of the neat compounds. The grey arrows are fixed parameters from the neat experiment and the black arrows are allowed to vary but are equal. 13

Section S5. Contour Plots of psTA spectra 14

Figure S14 – Contour plot of psTA spectra of CuCzMAC in toluene 16
Figure S15 – Contour plot of psTA spectra of AuCzMAC in toluene 16
Figure S16 – Contour plot of psTA spectra of CuBCzMAC in toluene 16
Figure S17 – Contour plot of psTA spectra of AuBCzMAC in toluene..17
Figure S18 – Contour plot of psTA spectra of AuBCzMAC in THF 17
Figure S19 – Contour plot of psTA spectra of CuPhCzMAC in toluene 17
Figure S20 – Contour plot of psTA spectra of CuCNCzDAC in toluene 18
Figure S21 – Contour plot of psTA spectra of CuCzCAAC in toluene 18
Figure S22 – Contour plot of psTA spectra of AuCzCAAC in toluene...18
Figure S23 – Contour plot of psTA spectra of AuCzCAAC in THF 19
Figure S24 – Contour plot of psTA spectra of CuBCzMAC in toluene with 280 mM MePI..................................19

Section S6. Operational Details for psTA and Target Analysis TA Fitting 20

Figure S25 – psTA spectra of AuCzCAAC in toluene. (a) AuCzCAAC in toluene along with the corresponding toluene only signal at the same time slices. (b) Spectrum recreated after subtraction of toluene only signal from panel (a). 22

Section S7. Species Associated Decay Spectra from psTA datasets 23

Figure S26 – SADS of CuCzCAAC (left) and CuCzMAC (right) in toluene 23
Figure S27 – SADS of AuCzCAAC (left) and AuCzMAC (right) in toluene...23
Figure S28 – SADS of CuBCzMAC (left) and AuBCzMAC (right) in toluene 24
Figure S29 – SADS of CuPhCzMAC (left) and CuCNCzDAC (right) in toluene..24
Figure S30 – SADS of AuBCzMAC (left) and AuCzCAAC (right) in THF. 24
Figure S31 – SADS of CuBCzMAC with 280 mM MePI in toluene 25

Section S8. Nanosecond Transient Absorption Spectra 26

Figure S32 – 355 nm pumped nsTA spectra of CuCzMAC in THF (left) and toluene (right)................................26
Figure S33 – 355 nm pumped nsTA spectra of AuCzMAC in THF (left) and toluene (right)................................26
Figure S34 – 355 nm pumped nsTA spectra of CuBCzMAC in THF (left) and toluene (right). 26
Figure S35 – nsTA spectra of AuBCzMAC with 420 nm pumped in THF (left) and 355 nm pumped in toluene (right). The psTA traces at 1.5 ns are displayed to demonstrate the effectiveness of the PL subtraction method presented here (see below). 27
Figure S36 – 355 nm pumped nsTA spectra of CuPhCzMAC in THF (left) and toluene (right). 27
Figure S37 – nsTA spectra of CuCNCzDAC in THF (left) and toluene (right). Degradation of CuCNCzDAC occurs with 355 nm illumination in THF...27
Figure S38 – 410 nm pumped nsTA spectra of CuCzCAAC in THF (UC Riverside). 28
Figure S39 – 355 nm pumped nsTA spectra of AuCzCAAC in THF (left) and toluene (right). 28
Figure S40 – 355 nm pumped nsTA spectra of 46 μM AuCzMAC and 7 mM MePI in THF (4 mL static cell).....28
Figure S41 – 420 nm pumped nsTA spectra of 75 μM AuBCzMAC and 30 mM MePI in THF (4 mL static cell). A peak attributed to the AuBCzMAC cation is observed in the earliest time traces. 29
Figure S42 – Normalized nsTA decay traces of AuBCzMAC and 30 mM MePI in THF, 420 nm excitation, 4 mL static cell. The TA trace of 750 nm– black and PL trace of 650 nm – red. The time axis is lin-log with the break at 0.1 μs. The inset is the same traces displayed on a log-log plot, demonstrating the PL intensity decays far below the TA. 29

Figure S43 – Normalized nsTA decay traces of AuBCzMAC and 100 mM BIH in THF, 450 nm excitation (100 mL circulating flow cell). Two probe wavelength (500 nm – black, 700 nm – blue) with identical kinetics before 10 μs are shown. The time axis is lin-log with the break at 0.1 μs. The inset is the same traces displayed on a log-log plot demonstrating the PL intensity decays identically to the TA traces. 30

Section S9. Fitting Schemes for Quenching nsTA........ 31

Figure S44 – Simplified kinetic model used for fitting the nsTA data, adapted from Figure S13, where states and processes are ignored to which the nsTA is insensitive. There are only two active compartments: T1 and cation or anion. For the unquenched experiments, only the T1 compartment (red), is considered, while the cation or anion compartment (green) is added for the quenching studies. (a) In quenching studies in THF, conversion of T1 to quenched ion was complete (b) whereas in toluene an excited state equilibrium was formed between cMa and quencher. 32

Section S10. Species Associated Decay Spectra (SADS) of nsTA spectra........ 33

Figure S45 – SADS of CuCzMAC (left) and CuBCzMAC (right) in toluene 33

Figure S46 – SADS of CuPhCzMAC (left) and CuCNCzDAC (right) in toluene 33

Figure S47 – SADS of AuCzMAC (left) and AuCzCAAC (right) in toluene 33

Figure S48 – SADS of AuBCzMAC in toluene (left) and in THF (right) 34

Section S11. SADS of nsTA Quenching Experiments 35

Figure S49 – nsTA SADS of AuBCzMAC in THF with MePI concentrations of (a) 6 mM and (b) 30 mM 35

Figure S50 – nsTA SADS of AuBCzMAC with 280 mM MePI in toluene (a); the fit was unable to separate the two states, but we simulated the resulting spectra using the basis spectra of both compartments. 35

Section S12. Comparison of Triplet SADS from psTA and nsTA........ 36

Figure S51 – SADS from psTA and nsTA of CuCzMAC (left) and CuBCzMAC (right) in toluene 36

Figure S52 – SADS from psTA and nsTA of CuPhCzMAC (left) and CuCNCzDAC (right) in toluene 36

Figure S53 – SADS from psTA and nsTA of AuCzMAC (left) and AuCzCAAC (right) in toluene 37

Figure S54 – SADS from psTA and nsTA of AuBCzMAC in toluene (left) and in THF (right). The right hand figure displays the SADS for the nsTA data with 405 nm pump (black), 355 nm pump (red) and 420 nm pump (blue). 37

Section S13. Simulation of the S1 and T1 ESA utilizing Pulse Radiolysis Spectra. 38

Figure S55 – The sum of the PR molar absorptivity plots (black) of CuCzMAC (left) and CuBCzMAC (right) compared to the S1 state (blue) and the T1 state (red) from SADS analysis 38

Figure S56 – The sum of the PR molar absorptivity plots (black) of CuPhCzMAC (left) and CuCNCzDAC (right) compared to the S1 state (blue) and the T1 state (red) from SADS analysis 39

Section S14. Simulation of the S1 and T1 ESA utilizing Bulk Electrolysis Spectra 40

Figure S57 – The sum of the BE spectra (black) of CuBCzMAC compared to the S1 state (blue) and the T1 state (red) from SADS analysis. The S1 SADS is from psTA and the T1 SADS is from nsTA. 41

Section S15. PL Correction in Magnitude Instruments 42

Figure S58 – nsTA of AuBCzMAC in toluene under varying values of PL subtraction parameter. (a) $s = 1$ (auto subtraction), (b) $s = 1.02$ (corrected), (c) $s = 0$ (no PL subtraction). (d) Normalized nsTA time traces at 550 nm under varying ***s*** values. The trace at 440 nm for the GSB has been inverted for overlap with the other traces. The PL at 570 nm is plotted as the blue-circle line. (e) Time traces at 570 nm under PL correction with different s values. 44

Section S16. Photostability Measurements 45

Figure S59 – Absorption spectra of cMa complexes under 460nm irradiation for varying amounts of time (a) CuBCzMAC in THF, (b) CuPhCzMAC in THF, (c) AuBCzMAC in THF, (d) AuBCzMAC in 1:3 Water to THF (by vol), (e) AuBCzMAC 5:1 MeCN to water (by vol), (f) AuBCzMAC 1:1 MeCN to water (by vol). 46

Figure S60 – Left) ns-TCSPC time traces with excitation at 405 nm for AuBCzMAC with various concentration of BIH in THF. Right) Stern-Volmer analysis of AuBCzMAC with various concentrations of BIH in THF. See Muniz *et al* reference for experimental procedures and analysis adopted here. 47

Figure S61 – Left) ns-TCSPC time traces with excitation at 450 nm for AuBCzMAC with various concentration of BIH in THF. Right) Stern-Volmer analysis of AuBCzMAC with various concentrations of BIH in THF. See Muniz *et al* reference for experimental procedures and analysis adopted here. 47

Section S17. Molar Absorption Spectrum of BIH.. 48

Figure S62 – Molar absorptivity spectrum of BIH in THF with absorptivities at the pump wavelengths indicated. 48

References.. 49

Section S1. Absorptivity Spectra of cMa ions from Pulse Radiolysis

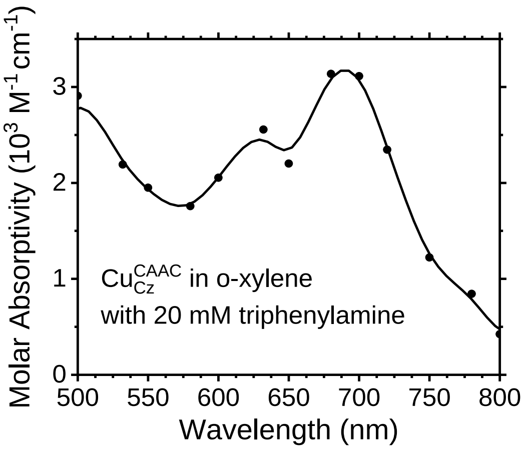

Figure S1 – Cation molar absorptivity spectra of 10 mM Cu_{Cz}^{CAAC} in 20 mM solution of triphenylamine in o-xylene. The circles are data points and the line is the interpolation between the symbols.

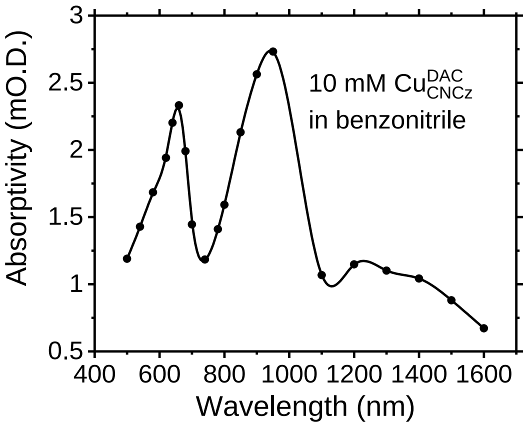

Figure S2 – Cation absorptivity of 10 mM Cu_{CNCz}^{DAC} in benzonitirile. The circles are data points and the line is the interpolation between the symbols.

Section S2. Time Correlated Single Photon Counting Decay Curves

Depicted below are the TCSPC curves for the cMa compounds in both toluene and THF pumped at 400 nm. The values for τ_P, A_P, and $(1 - A_p)$, are depicted as well. The longer time range data is displayed as the inset. For most compounds, TCSPC was collected with an instrument described in the main text with an IRF ~ 22ps. However, for M_{Cz}^{MAC} and M_{Cz}^{CAAC}, lifetimes were measured with a Horiba Fluorohub A+ equipped with a 405 nm laser source (< 300 ps IRF). Lifetime measurements were acquired from solutions at maximum optical densities between 0.1 and 0.2 to minimize effects of solute-solute interactions.

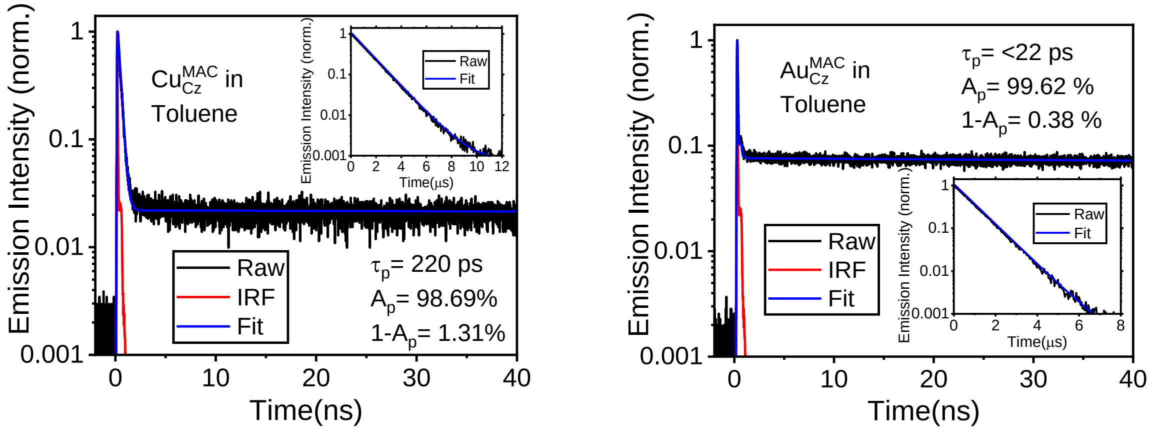

Figure S3 – Emission decay trace for Cu$_{Cz}^{MAC}$ (left) and Au$_{Cz}^{MAC}$ (right) in toluene.

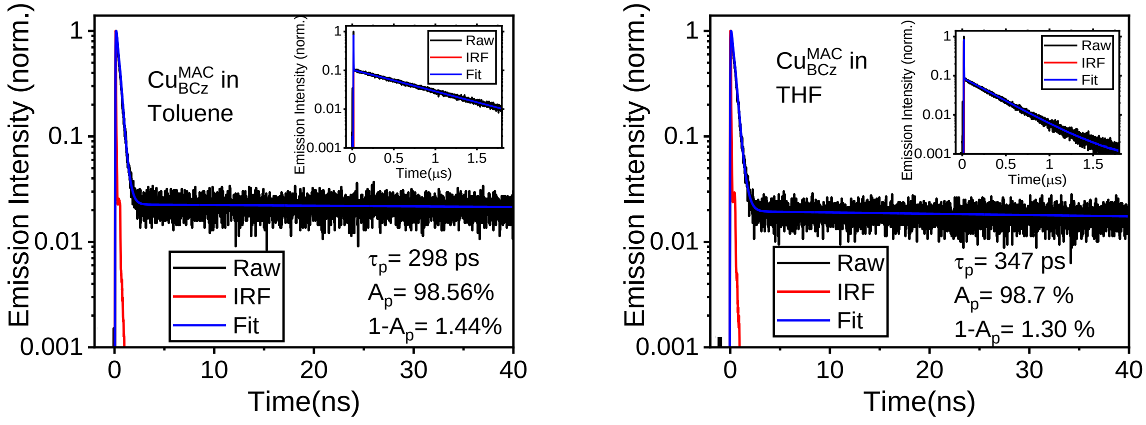

Figure S4 – Emission decay traces for Cu_{BCz}^{MAC} in toluene (left) and in THF (right).

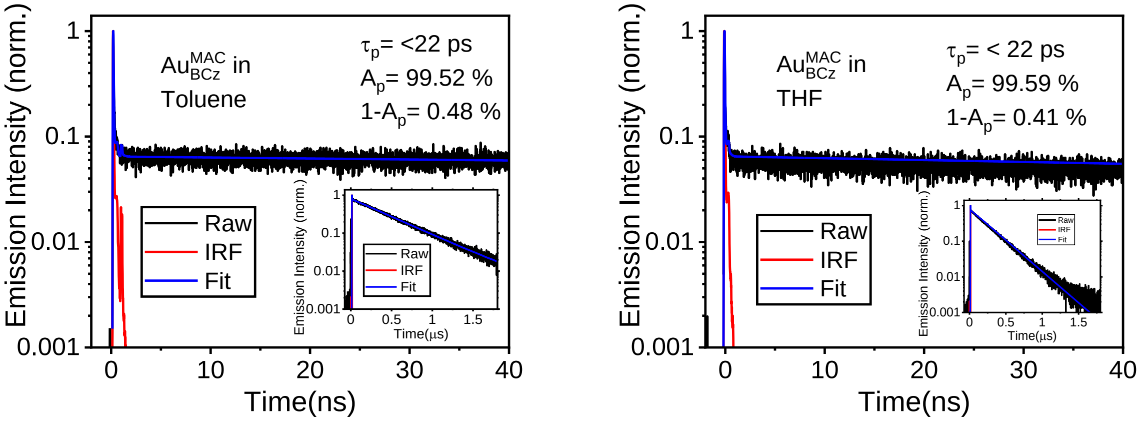

Figure S5 – Emission decay traces for Au_{BCz}^{MAC} in toluene (left) and in THF (right).

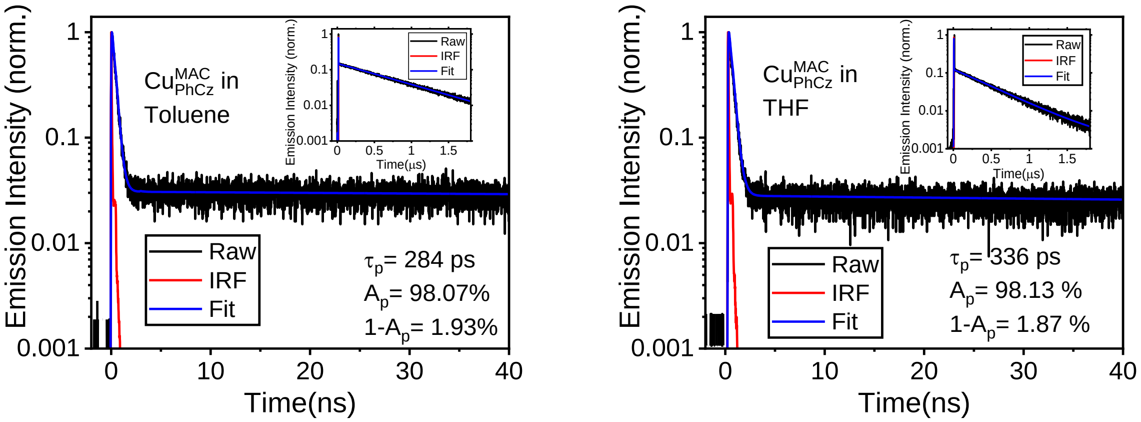

Figure S6 – Emission decay traces for Cu_{PhCz}^{MAC} in toluene (left) and in THF (right).

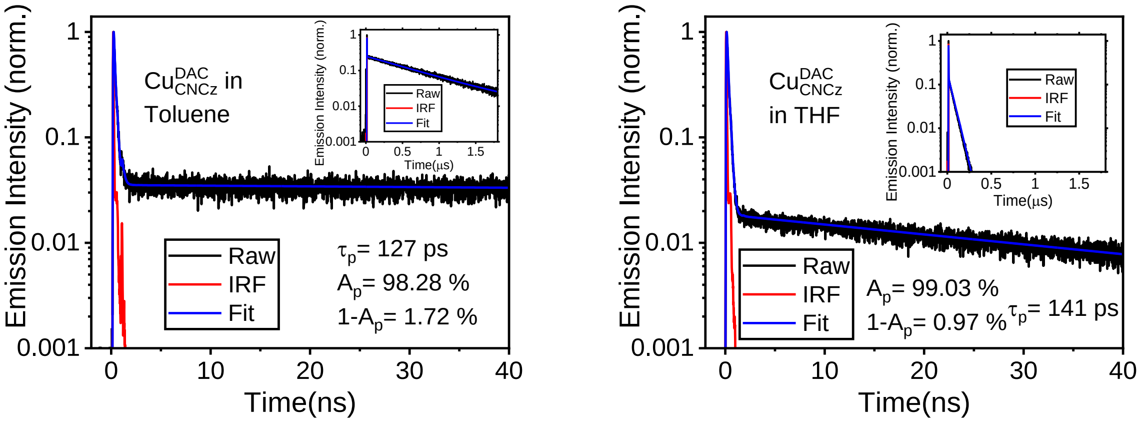

Figure S7 – Emission decay traces for Cu_{CNCz}^{DAC} in toluene (left) and in THF (right).

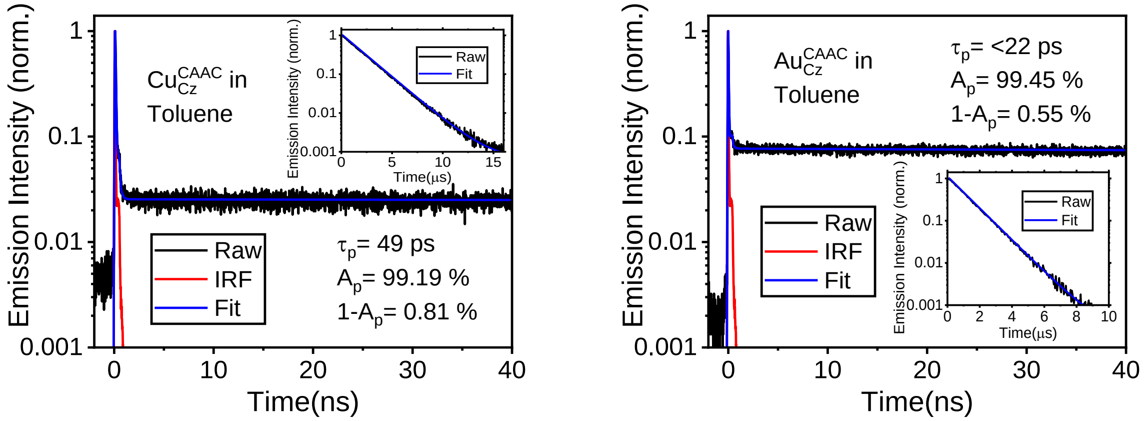

Figure S8 - Emission decay trace for Cu_{Cz}^{CAAC} (left) and Au_{Cz}^{CAAC} (right) in toluene.

Section S3. Picosecond Transient Absorption Data on changing the carbazole unit and solvent

In this section, we provide psTA datasets for the full set of compounds studied. The first datasets are the remaining cMa compounds in toluene: Cu_{BCz}^{MAC}, Cu_{PhCz}^{MAC}, and Cu_{CNCz}^{DAC}. Here, we discuss the effect the carbazole has on the entire ESA feature of three cMa compounds in the Cu^{MAC} class with reference to Cu_{Cz}^{MAC} from the main article. The carbazole is the largest contributor to the ESA line shape but substitutions to the carbazole unit leads to the smallest spectral changes. This can be seen by comparing the relative peak heights of the ESA of the Cu_{Cz}^{MAC}, Cu_{BCz}^{MAC}, and Cu_{PhCz}^{MAC} at 1 ns for the triplet which have a relative ratio of ~1.2 (Figure S9**, Figure 9**). The most significant change is the positioning of the S_1 690 nm peak in Cu_{Cz}^{MAC} is now redshifted to 730 nm in Cu_{PhCz}^{MAC}. Cu_{BCz}^{MAC} remains unshifted compared to Cu_{Cz}^{MAC}. Due to the redshift of the ESA of the PhCz ligand, the SE carves out more of the ESA in Cu_{PhCz}^{MAC}. The Cu_{PhCz}^{MAC} peak is obscured due to the weak probe intensity in the region near 800 nm. Cu_{CNCz}^{DAC} is also displayed in Figure S10. We note that two ligand substitutions have been made going from $Cu_{BCz/PhCz}^{MAC}$ to Cu_{CNCz}^{DAC}. We can see clear evidence of the evolution of the SE "dip" toward the steady state fluorescence spectra and the ESA. The strongest ESA signature for Cu_{CNCz}^{DAC}, as is for all compounds presented, is the triplet which occurs between 500 – 700 nm (**Figure 4(b)**). Comparing the PR spectra of the cation from Figure S2, we see a similar structureless feature, albeit shifted a similar amount as presented for the other cMa compounds. The reduced spectral range is due to the use of a less dispersive grating for this particular experiment.

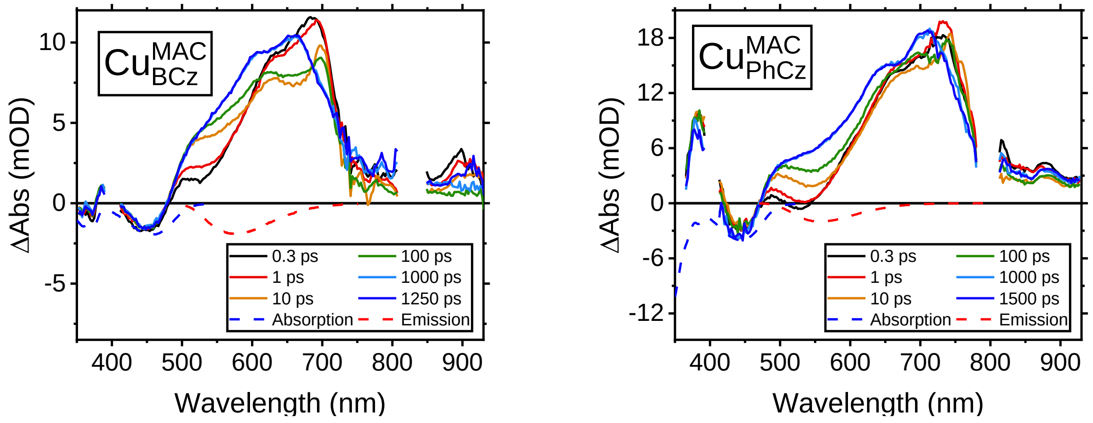

Figure S9 – psTA spectra of Cu_{BCz}^{MAC} (left) and Cu_{PhCz}^{MAC} (right) in toluene.

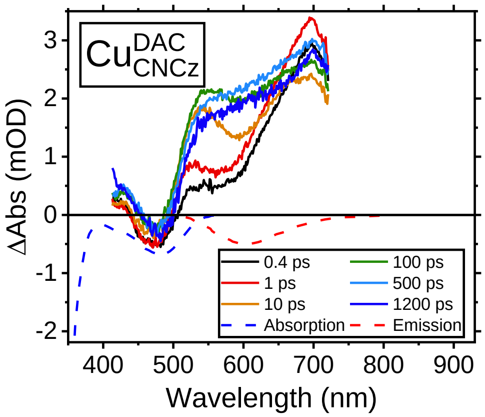

Figure S10 – psTA spectra of Cu_{CNCz}^{DAC} in toluene.

In the main text, we showed that a change of solvent around Au_{BCz}^{MAC} led to more dramatic spectral evolution in the more polar THF, but little change in the important ISC rate constants. Here we show another example, the psTA spectra for Au_{Cz}^{CAAC} in THF. Again, a significant red shifting of the SE is seen. Once again, if we compare to Au_{Cz}^{CAAC} in toluene, target analysis fitting reveals once again little change in k_{ISC}^{exe} (within error) on change of solvent polarity. We have used the 1.5 ns trace recorded in this experiment to properly correct the PL subtraction of the corresponding nsTA experiment.

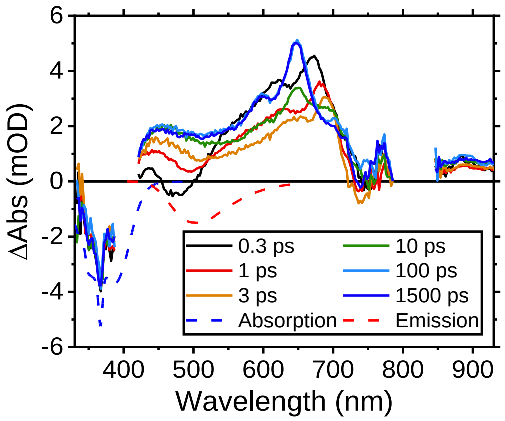

Figure S11 – The psTA spectrum of Au_{Cz}^{CAAC} in THF.

Section S4. Charge transfer within a 1 ns window with concentrated quencher

We end this section by showing a dataset which shows effective and rapid quenching of Cu_{BCz}^{MAC} with 280 mM MePI in toluene to obtain $Cu_{(BCz)^+}^{MAC}$ with the spectrum displayed in Figure S12. Following the analysis performed in the main article on the compounds without quencher, we assign similar features to Cu_{BCz}^{MAC} with 280 mM MePI as we did with Au_{BCz}^{MAC} (**Figure 8**). The early time feature at 300 fs is assigned to S_1^*. The relaxation of S_1^* to S_1 occurs on the order of 5 ps, consistent with the neat experiment. The spectrum at 10 ps corresponds to the S_1 state. The apparent full SE loss from S_1^* to S_1 is likely because some quenching has begun to occur to bring the entire spectrum closer to baseline producing an apparent SE loss. The region of SE has flattened by ~2x as the delay advances from 10 to 100 ps. We assign the new positive feature at λ > 700 nm to $Cu_{(BCz)^+}^{MAC}$ based on good agreement with SEC. Importantly, this indicates that quenching of Cu_{BCz}^{MAC} in toluene with MePI occurs on a 100s ps timescale and provides a useful contrast to the slower quenching in the nsTA experiment of Au_{BCz}^{MAC} in toluene with a lower concentration of MePI (**Figure 11d**).

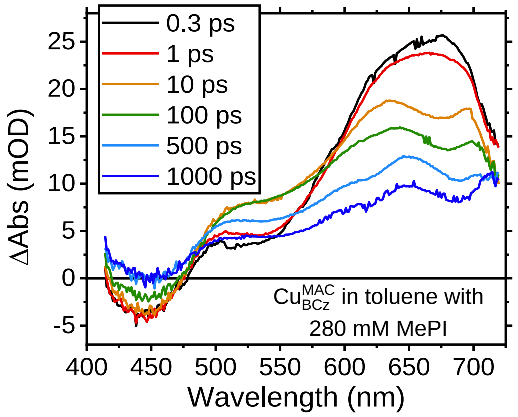

Figure S12 – The psTA spectrum of Cu_{BCz}^{MAC} in toluene with 280 mM MePI.

Determination of k_q was obtained using a fitting model that was an extension of **Figure 7**, displayed here as Figure S13. A fourth state (green) is added as the trapped pair, $Cu_{(BCz)^+}^{MAC}$/MePI$^-$. Here, we have to assume quenching from both the singlet and the triplet but we assign the same rate constant (k_q) as

the excited state reduction potential only varies by a small ΔE_{ST}.[1] If we do not include singlet transfer, we find physically unreasonable SADS as well as contamination in the T_1 SADS from the cation absorption. The other kinetic parameters (grey arrows) are fixed from the neat Cu_{BCz}^{MAC} experiment. No decay pathway from the trapped pair was considered as the trapped pair for a similar system, Au_{BCz}^{MAC} with MePI in toluene, has a lifetime 110 ns and so the psTA will not have decayed appreciably by 1 ns, the total length of this psTA experiment.

A good fit was found for k_q·[MePI] = 2.9·10^9 s^{-1} and by dividing by concentration, 280 mM, k_q = (10 ± 8) ·10^9 M^{-1} s^{-1}. Error bar values were obtained by iterating k_q with goodness of fit based on a physically meaningful triplet spectrum from the SADS (this is displayed in the SADS of psTA section). Deviations of the fitted triplet spectrum from the neat experiment were rejected. This value varies from the values reported by Muniz *et al.*, which has a k_q ~4x smaller than the value reported here.[1] However, we are using two orders of magnitude higher concentration of quencher as well as a different solvent. Whether linearity over this wide quencher concentration regime is reasonable is yet to be established.

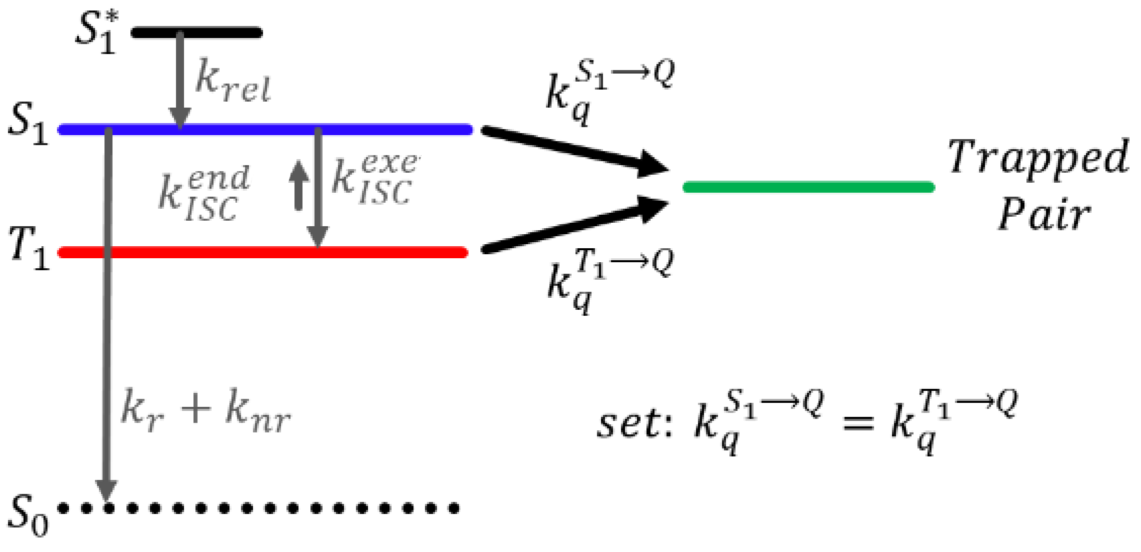

Figure S13 – Kinetic model used for fitting the quenching of Cu_{BCz}^{MAC} with MePI. The left-hand side is the same model as the psTA fitting of the neat compounds. The grey arrows are fixed parameters from the neat experiment and the black arrows are allowed to vary but are equal.

Section S5. Contour Plots of psTA spectra

Here we display the contour plots of the psTA spectra of the cMa compounds pumped at 405 nm. Similar conditions were run for each sample. The x-axis is time in ps, plotted as linear-logarithmic axis with a break at 5 ps. The delay times before 5 ps are plotted linearly and times after 5 ps are plotted logarithmically. The y-axis is wavelength in nm, and the z-axis is ΔAbs values in mOD. Positive ΔAbs values tend red, negative values tend blue and values near zero are white. Please note the range on the colorbar changes with each dataset. The z-axis values were truncated so the max value on the colorbar is taken from t > 340 fs to avoid emphasizing the coherent response of the solvent. A coherent artifact is generated at time zero due to two-photon absorption (2PA) from toluene and cross-phase modulation (XPM) from the cuvette walls.[2, 3] The coherent response appears as a red feature in the region of t = ± 300 fs and λ < 450 nm.

The psTA data were corrected for white light chirp both before displaying the present contour plots and fitting the datasets. In addition to the IRF signal at λ < 420 nm and time zero, another similar feature appears from -1 to -0.4 ps and 725 to 775 nm. This second feature reappears at twice the wavelength due to the 2nd order diffraction in the spectrometer. Before white light chirp correction, this feature appeared at the same time as the 2PA in the 400 to 450 nm region and after correction, this feature appears at negative time. The pump-probe signal in the λ > 700 nm range is also contaminated. By taking the ratio of the peaks of the contamination at 750 nm to the inherent coherent artifact at 370 nm, we find the contamination is 5-20%, varying between compounds. The GSB region occurs λ^1 = 350 – 450 nm, and the 2nd order diffraction would then be λ^2 = 700 – 900 nm. Therefore, the ESA in the 700-900 nm region is dampened by 5-20%. This effect will be revisited in the T_1 SADS comparison between psTA and nsTA.

The white streaks along λ = 400 and 800 nm are due to pump scatter removal. These regions differ in exclusion size due to pump scatter intensity varying between each experiment. The region of 725 to 850 nm (specific wavelengths varying for each experiment) was averaged with a 5 pt smoothing. An exception is made with Cu_{Cz}^{MAC} in toluene where the 800 nm region is smoothed by a 50 pt smoothing mandated by a

weaker signal to noise ratio compared to the other spectra. The smoothed data was used for fitting the data to obtain k_{ISC}^{exe} for the gold complexes and to obtain the species associated decay spectra (SADS) for every compound.

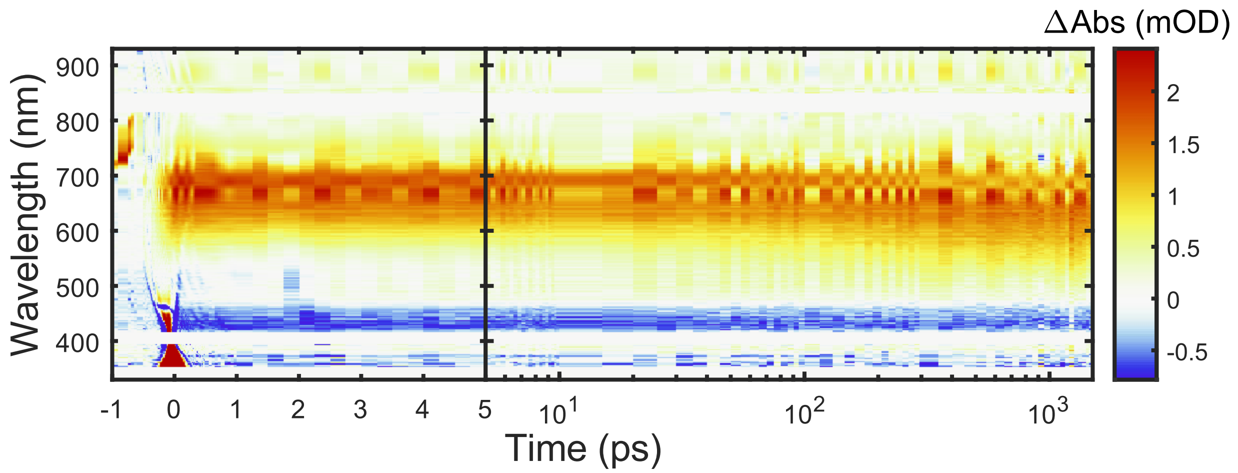

Figure S14 – Contour plot of psTA spectra of Cu_{Cz}^{MAC} in toluene

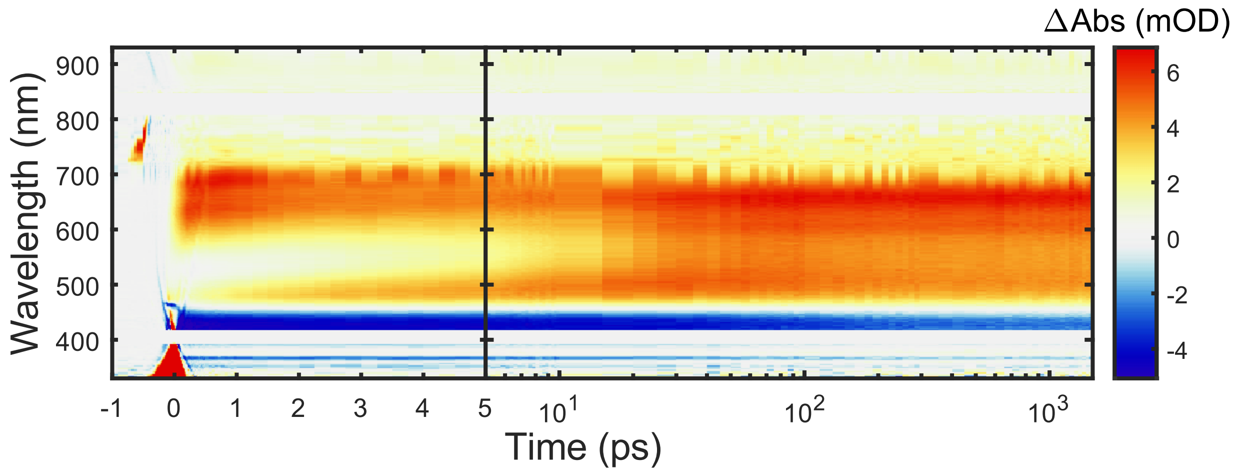

Figure S15 – Contour plot of psTA spectra of Au_{Cz}^{MAC} in toluene

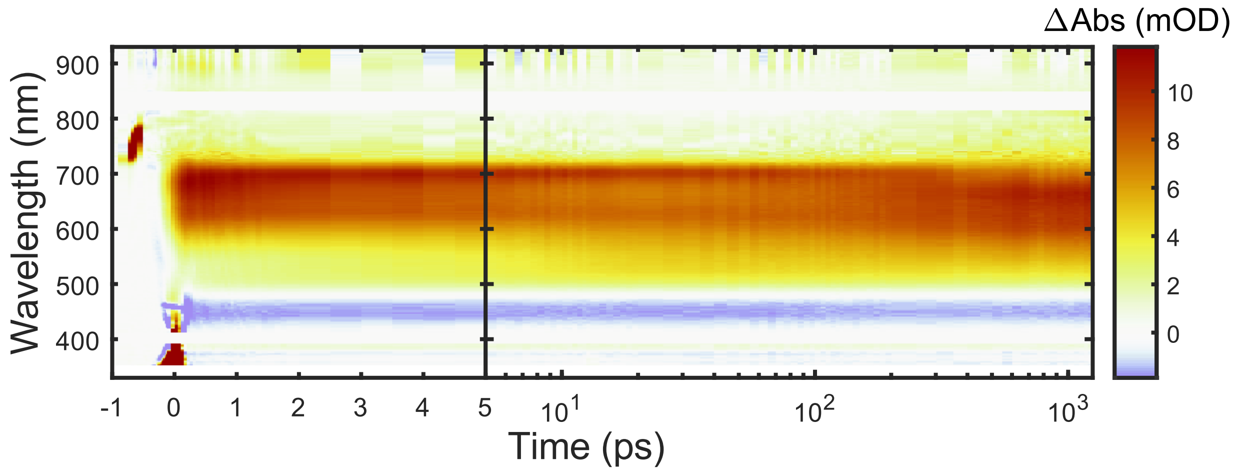

Figure S16 – Contour plot of psTA spectra of Cu_{BCz}^{MAC} in toluene

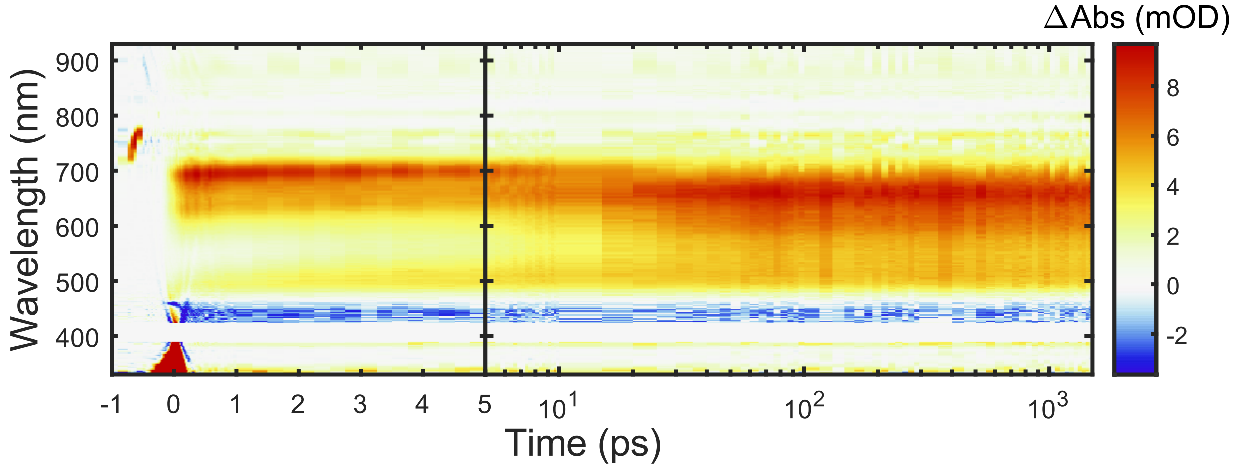

Figure S17 – Contour plot of psTA spectra of Au_{BCz}^{MAC} in toluene

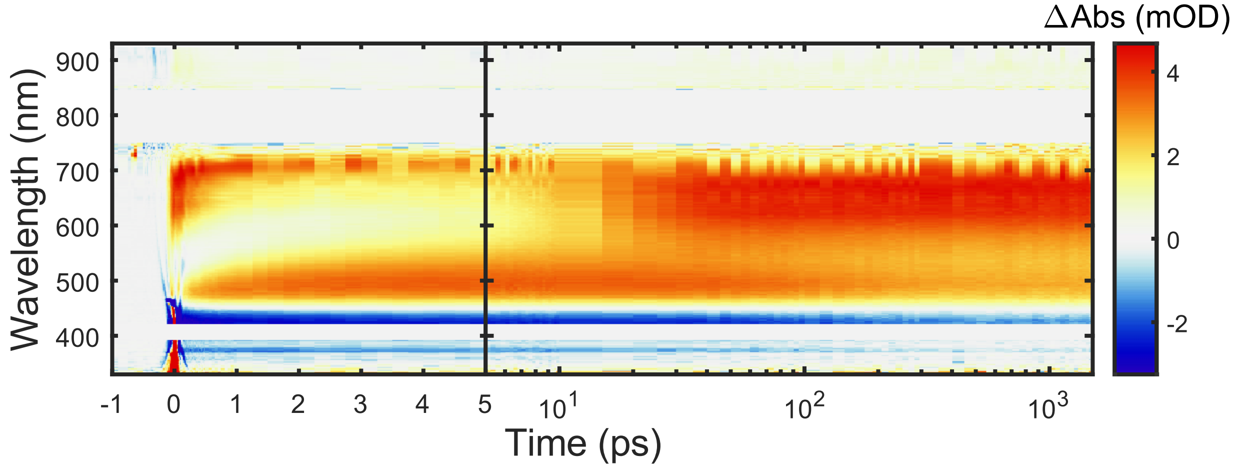

Figure S18 – Contour plot of psTA spectra of Au_{BCz}^{MAC} in THF

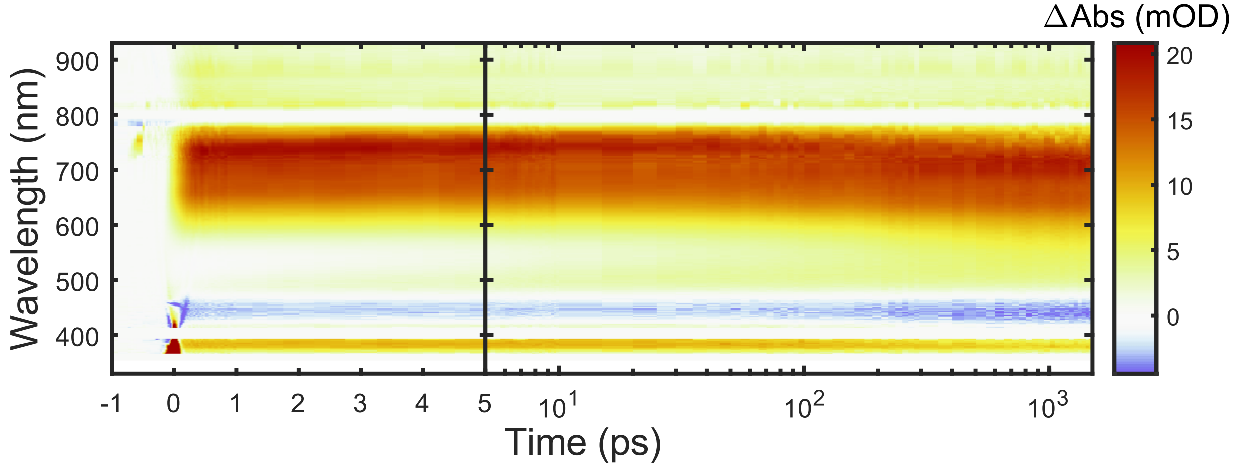

Figure S19 – Contour plot of psTA spectra of Cu_{PhCz}^{MAC} in toluene

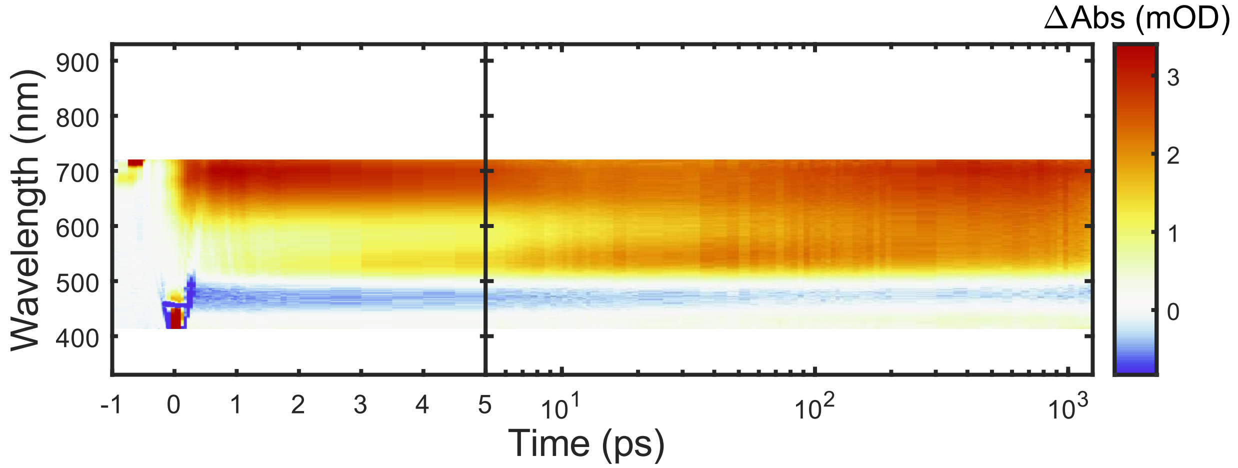

Figure S20 – Contour plot of psTA spectra of Cu_{CNCz}^{DAC} in toluene

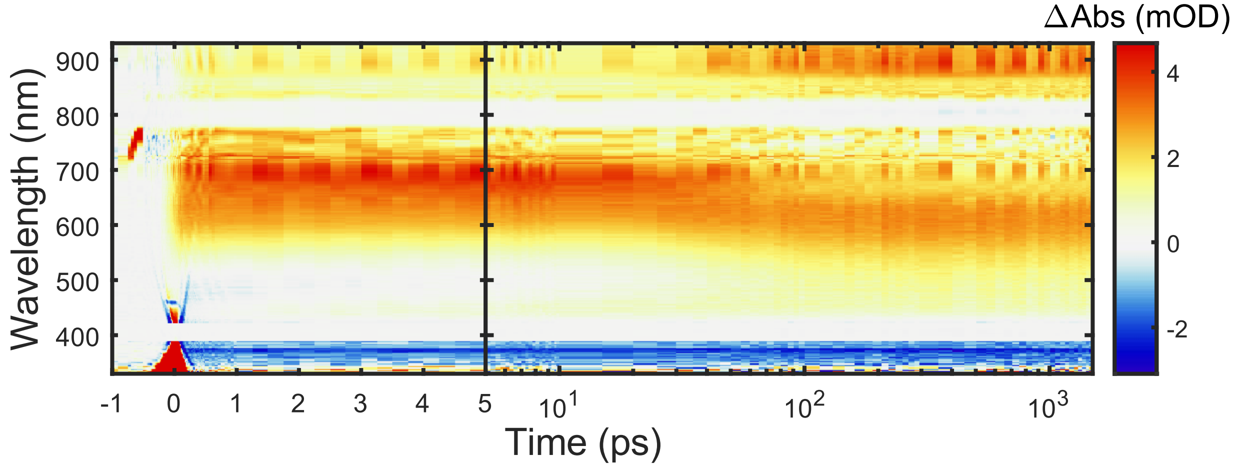

Figure S21 – Contour plot of psTA spectra of Cu_{Cz}^{CAAC} in toluene

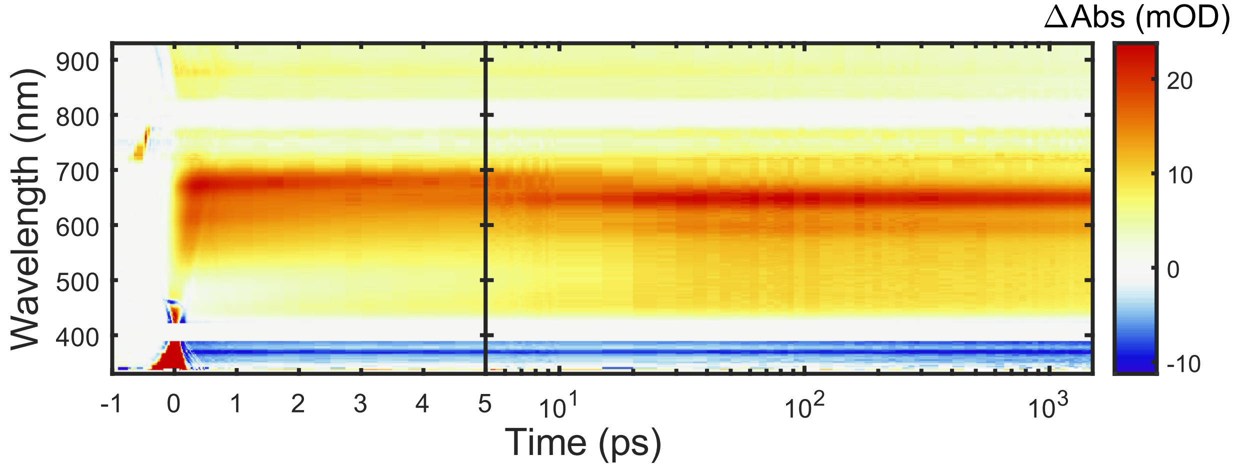

Figure S22 – Contour plot of psTA spectra of Au_{Cz}^{CAAC} in toluene

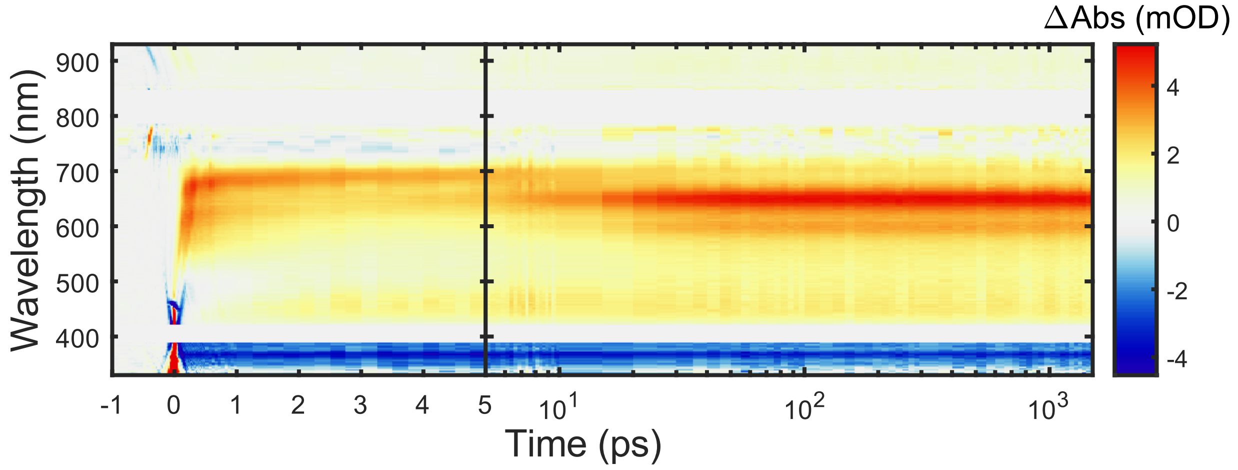

Figure S23 – Contour plot of psTA spectra of Au_{Cz}^{CAAC} in THF

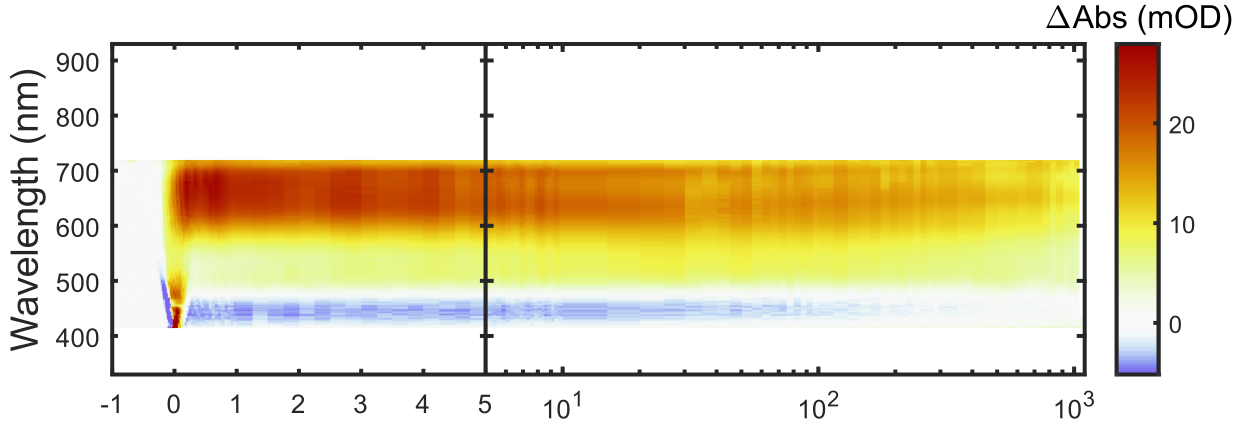

Figure S24 – Contour plot of psTA spectra of Cu_{BCz}^{MAC} in toluene with 280 mM MePI

Section S6. Operational Details for psTA and Target Analysis TA Fitting

In this section, we describe the use of target analysis to provide a full spectral and kinetic fit of each TA dataset.[4] Target analysis is defined by photophysical and photochemical parameters which extract meaningful information and provide a useful description of the transient 2D data. The fitted dataset is composed of a user-given number of compartments each further composed of a time-independent spectrum and time-resolved population. To obtain the ultrafast solvent and vibrational relaxation, the rate constants for the establishment of the ISC equilibrium (psTA), lifetimes, quenching rates (nsTA), and the species associated decay spectra (SADS), a target analysis and fitting program was used, referred here as PDP (developed by Mikas Vengris, Laser Research Facility, Physics Dept, Vilnius University). The PDP code uses a master solution to the kinetic equations based on a target model provided by the user. The scheme in **Figure 7** was used as the target model for each cMa psTA experiment. First, we will discuss the fitting procedure of the psTA, then the nsTA.

For each experimental run of a day, one or several cMa compounds would be run along with a matching solvent only spectrum. The solvent-only spectrum was used to determine several key items. First, the IRF width of the experiment can be determined by plotting the wavelength of the strongest signal of the coherent artifact as a function of time, focusing on time zero region. The IRF containing only positive 2PA signal is at 350 nm and was fit to a single gaussian. The full width half maximum (FWHM) of the gaussian was taken as the IRF width. The IRF width varied from 300 to 340 fs from experiment to experiment. Second, proper excitation conditions are determined by performing a solvent scan. With high pump fluence, the solvent can undergo two-photon absorption (2PA) and generate a solvent excited state. This can remove excitation energy from the cMa, producing undesired artifacts. An example of this is displayed in Figure S25, where

the pump fluence was too high and a subtraction of the toluene only signal from the Au_{Cz}^{CAAC} spectrum was required. This was the only spectra this subtraction was needed for and applied to.

The time zero artifacts of cross-phase modulation and 2PA are significant and can skew fitting to bias these features. To avoid biasing these features, the fitting around time zero was ignored by adding a weighting function with the center at time zero, a width of ~300 fs, with zero weight in this region. Time zero was allowed to float but fixed within ± 50 fs due to prior chirp correction.

A good fit was judged with the following procedure in mind. Above all, a fit for a given cMa compound must be physically and chemically sensible. As an example, if an excited state absorption spectrum is known, from other measurements like SEC, for which an ESA is always positive, a negative component in the SADS is discarded. The PDP fitting program was tailored and appropriately tuned so that artifacts like these are rejected. With meaningful SADS, the time trace at every probe wavelength, and the spectrum at every time delay was reviewed, and the goodness of fit was judged by eye. A plot of fit residual is also used to determine systematic divergence between model and data.

To determine k_{rel}, the time traces of several wavelengths were monitored for the goodness of fit at early times (< 10 ps) and iterated by 1 ps till the fit was best. For copper cMa complexes, the values of k_{ISC}^{exe} and k_{ISC}^{end} were obtained and fixed from TCSPC due to higher sensitivity to these parameters in TCSPC. The values of k_{ISC}^{exe} for the gold cMa complexes were obtained in a similar manner as k_{rel}. The sensitive range for k_{ISC}^{exe} was 10 to 100 ps. The values for k_{ISC}^{exe} were input and fixed in fluorescence fitting, which includes convolution with the measured IRF, to more accurately determine A_p and thus provide values for k_{ISC}^{end}. The errors in the k_{rel} and k_{ISC}^{exe} values

for the gold complexes were obtained via iteration and determination of quality of fit by hand therefore establishing an estimate for confidence limits.

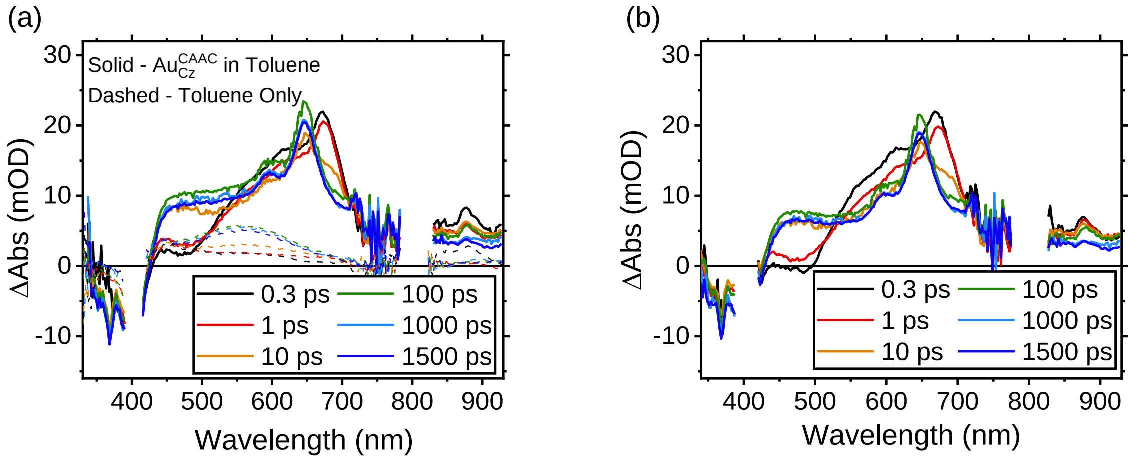

Figure S25 – psTA spectra of Au_{Cz}^{CAAC} in toluene. (a) Au_{Cz}^{CAAC} in toluene along with the corresponding toluene only signal at the same time slices. (b) Spectrum recreated after subtraction of toluene only signal from panel (a).

Section S7. Species Associated Decay Spectra from psTA datasets

In this section, we present the species associated decay spectra (SADS) obtained from fitting the psTA data. When these basis spectra are multiplied by the time dependent concentrations of each state, we recover the complete fitted psTA data. The SADS are the transient signatures associated with each distinct kinetic state displayed in the model of **Figure 7**. The fitting procedure is discussed in the previous section, Section S6.

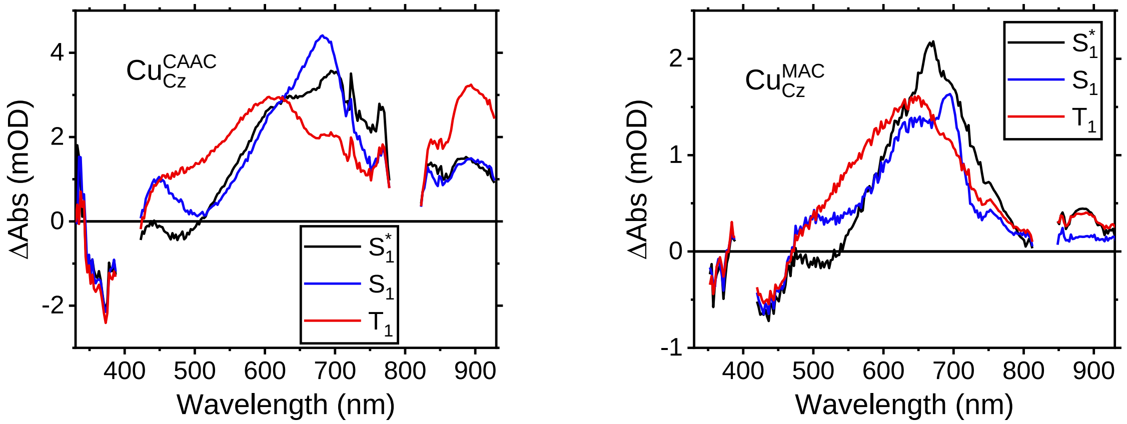

Figure S26 – SADS of Cu_{Cz}^{CAAC} (left) and Cu_{Cz}^{MAC} (right) in toluene

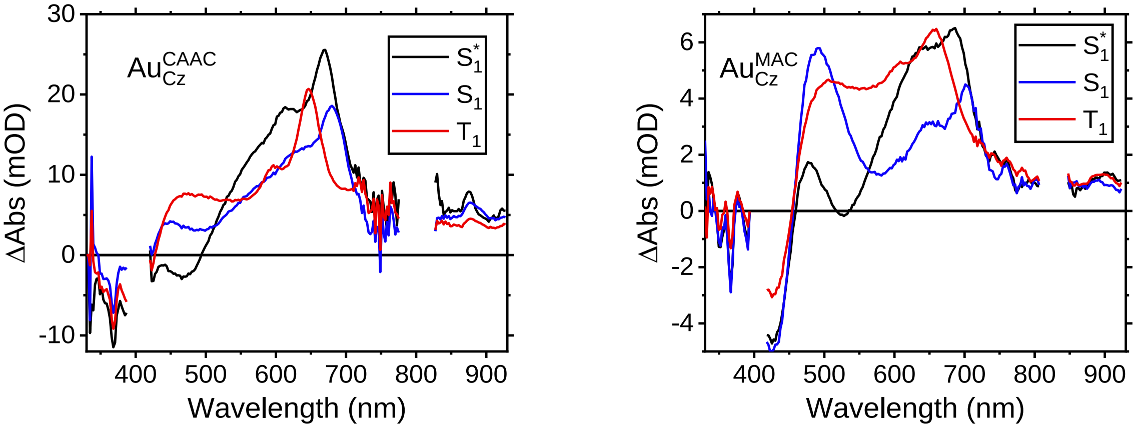

Figure S27 – SADS of Au_{Cz}^{CAAC} (left) and Au_{Cz}^{MAC} (right) in toluene

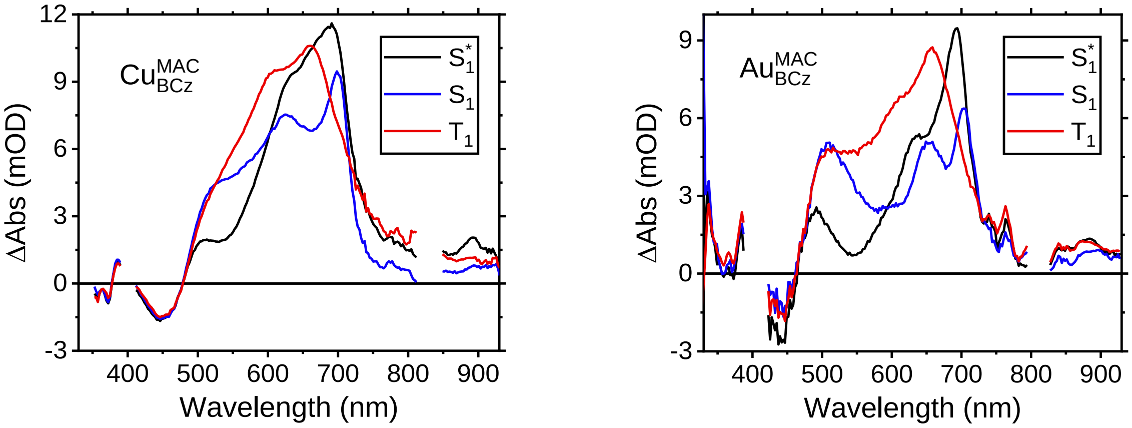

Figure S28 – SADS of Cu^{MAC}_{BCz} (left) and Au^{MAC}_{BCz} (right) in toluene

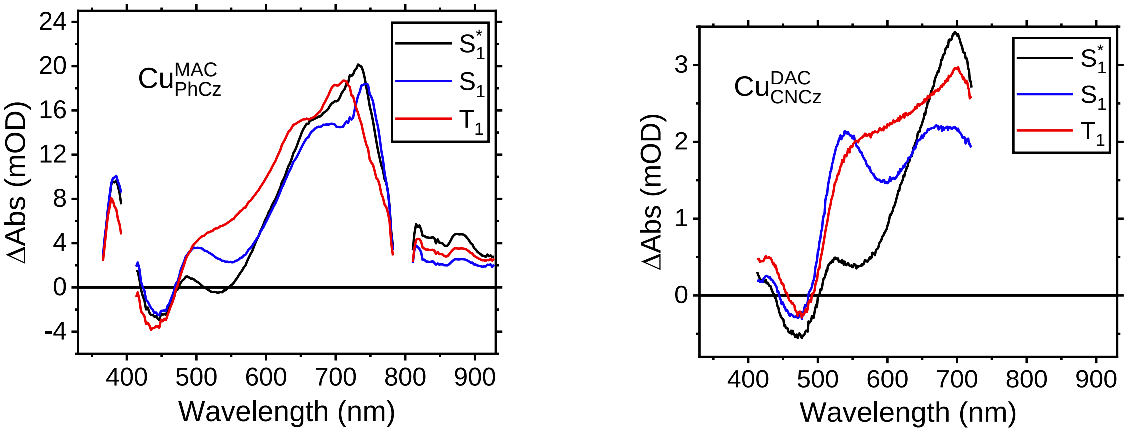

Figure S29 – SADS of Cu^{MAC}_{PhCz} (left) and Cu^{DAC}_{CNCz} (right) in toluene

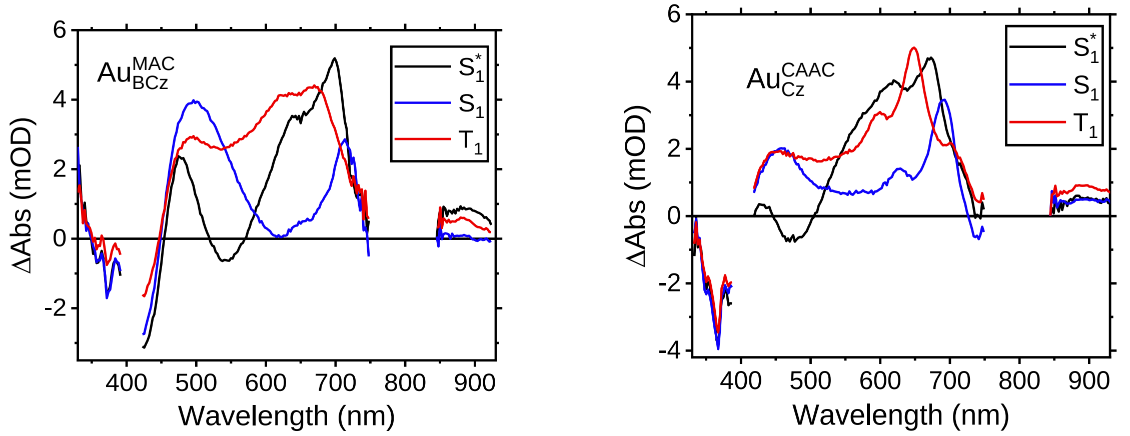

Figure S30 – SADS of Au^{MAC}_{BCz} (left) and Au^{CAAC}_{Cz} (right) in THF.

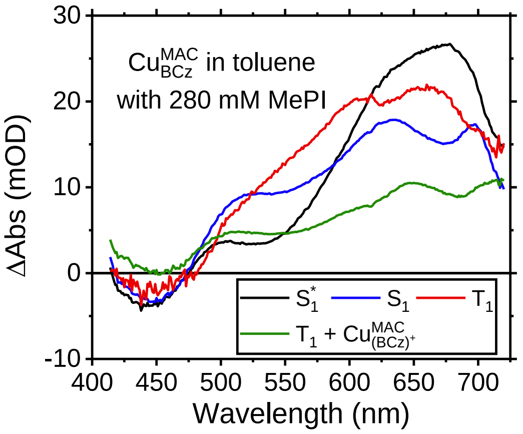

Figure S31 – SADS of Cu_{BCz}^{MAC} with 280 mM MePI in toluene

Section S8. Nanosecond Transient Absorption Spectra

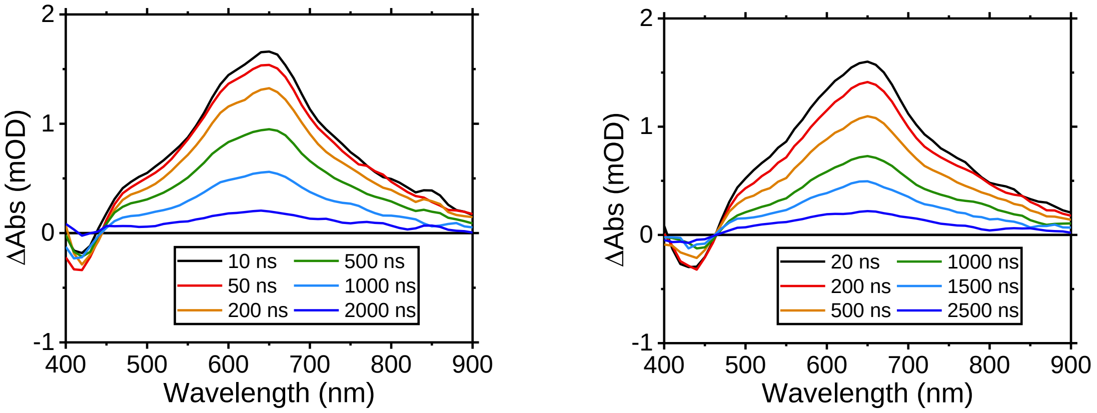

Figure S32 – 355 nm pumped nsTA spectra of Cu_{Cz}^{MAC} in THF (left) and toluene (right).

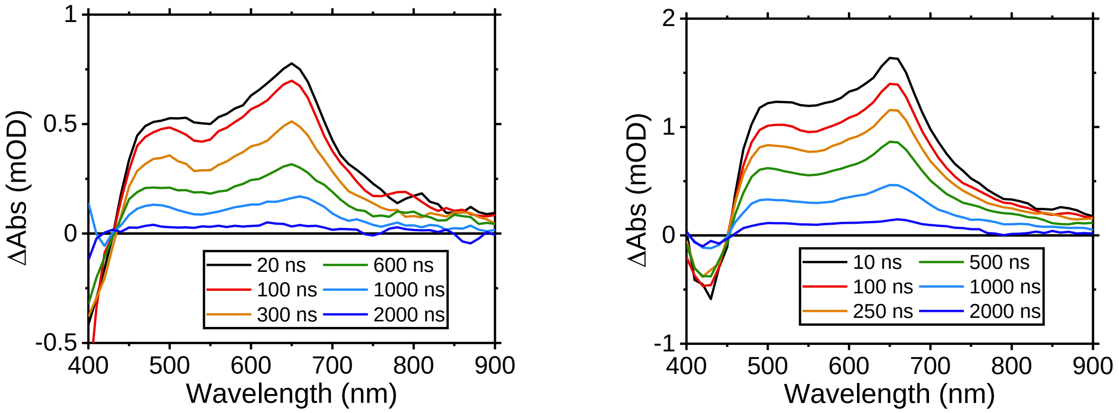

Figure S33 – 355 nm pumped nsTA spectra of Au_{Cz}^{MAC} in THF (left) and toluene (right).

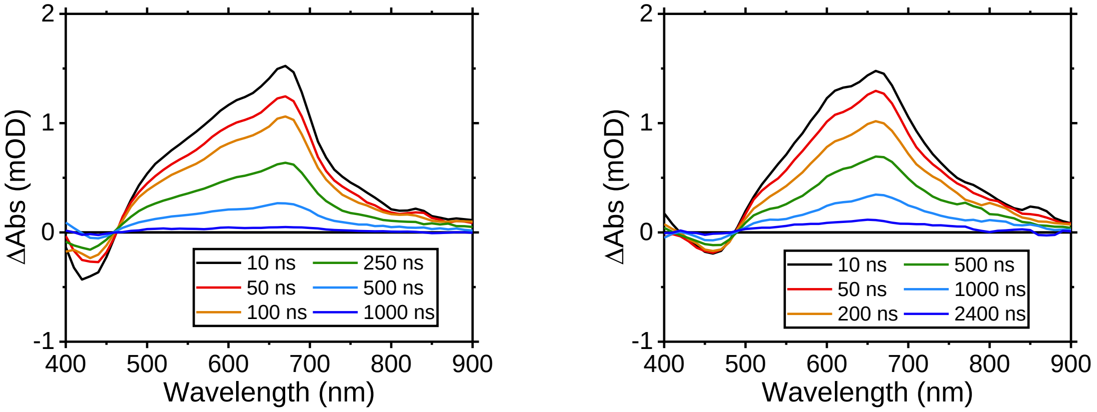

Figure S34 – 355 nm pumped nsTA spectra of Cu_{BCz}^{MAC} in THF (left) and toluene (right).

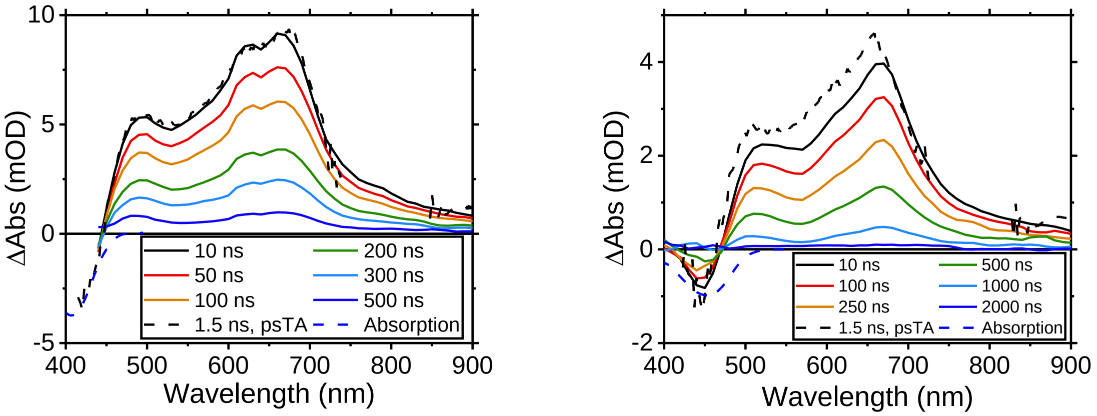

Figure S35 – nsTA spectra of Au_{BCz}^{MAC} with 420 nm pumped in THF (left) and 355 nm pumped in toluene (right). The psTA traces at 1.5 ns are displayed to demonstrate the effectiveness of the PL subtraction method presented here (see below).

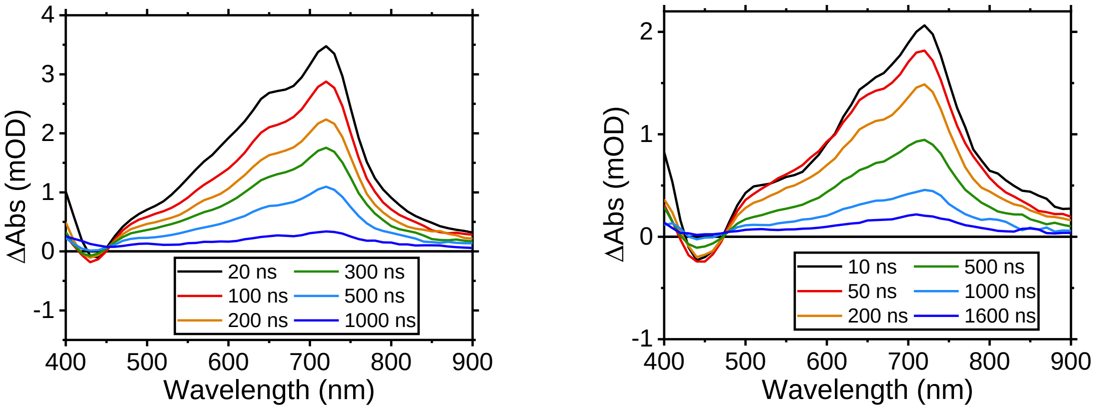

Figure S36 – 355 nm pumped nsTA spectra of Cu_{PhCz}^{MAC} in THF (left) and toluene (right).

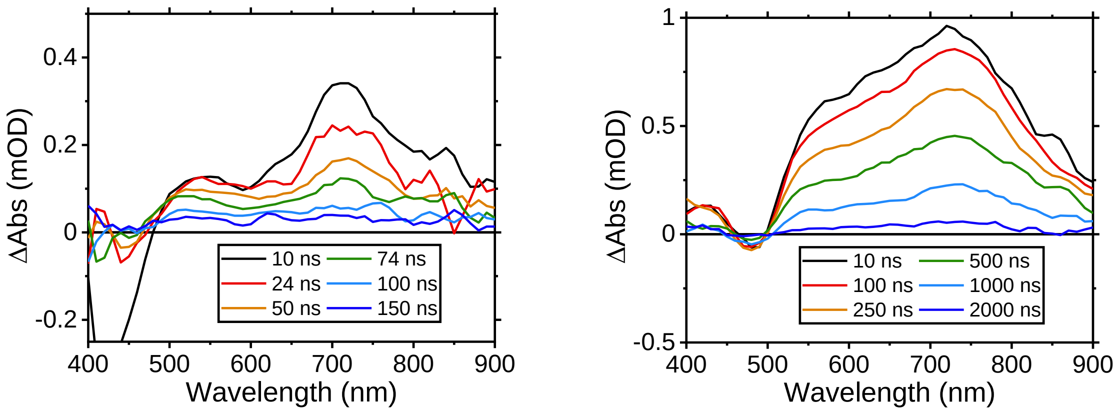

Figure S37 – nsTA spectra of Cu_{CNCz}^{DAC} in THF (left) and toluene (right). Degradation of Cu_{CNCz}^{DAC} occurs with 355 nm illumination in THF.

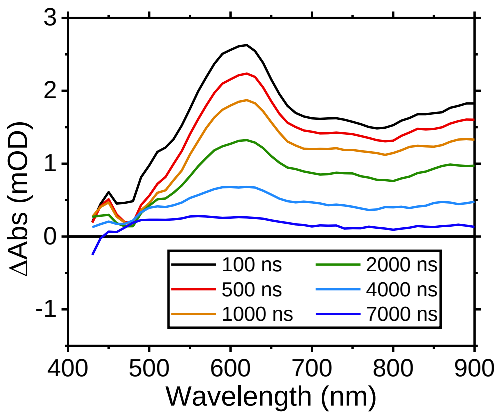

Figure S38 – 410 nm pumped nsTA spectra of Cu_{Cz}^{CAAC} in THF (UC Riverside).

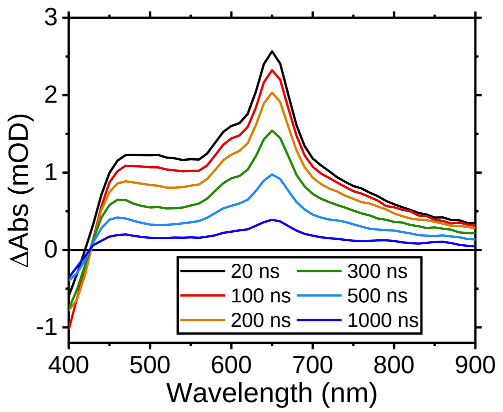

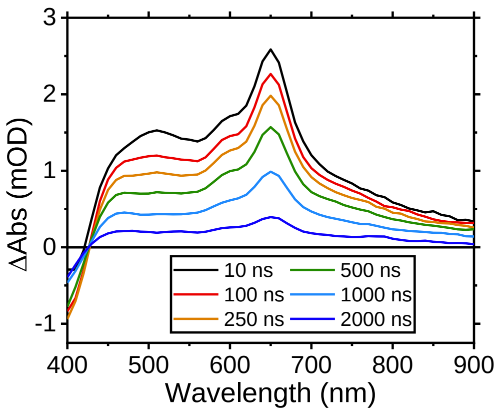

Figure S39 – 355 nm pumped nsTA spectra of Au_{Cz}^{CAAC} in THF (left) and toluene (right). We note sample decomposition followed the completion of taking these data.

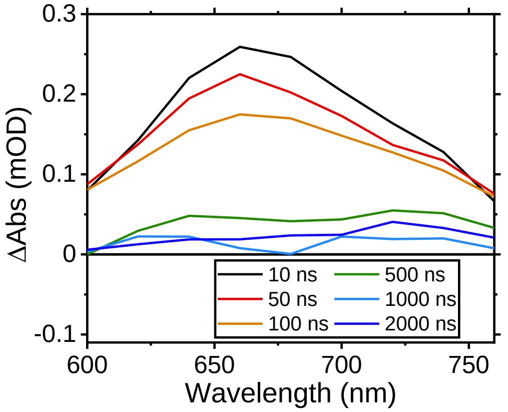

Figure S40 – 355 nm pumped nsTA spectra of 46 μM Au_{Cz}^{MAC} and 7 mM MePI in THF (4 mL static cell).

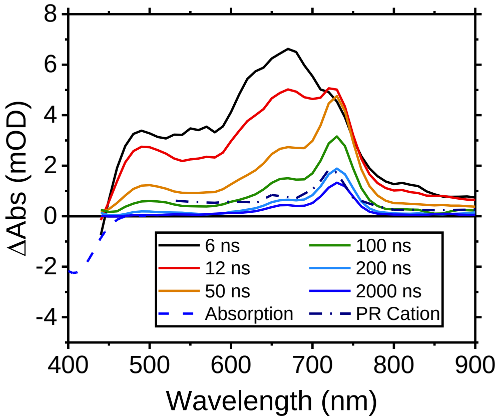

Figure S41 – 420 nm pumped nsTA spectra of 75 μM Au_{BCz}^{MAC} and 30 mM MePI in THF (4 mL static cell). A peak attributed to the Au_{BCz}^{MAC} cation is observed in the earliest time traces.

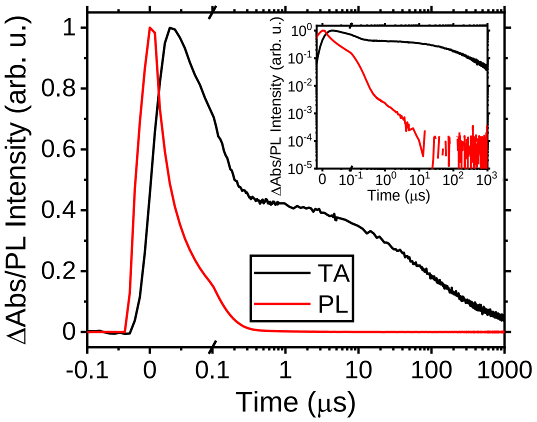

Figure S42 – Normalized nsTA decay traces of Au_{BCz}^{MAC} and 30 mM MePI in THF, 420 nm excitation, 4 mL static cell. The TA trace of 750 nm– black and PL trace of 650 nm – red. The time axis is lin-log with the break at 0.1 μs. The inset is the same traces displayed on a log-log plot, demonstrating the PL intensity decays far below the TA.

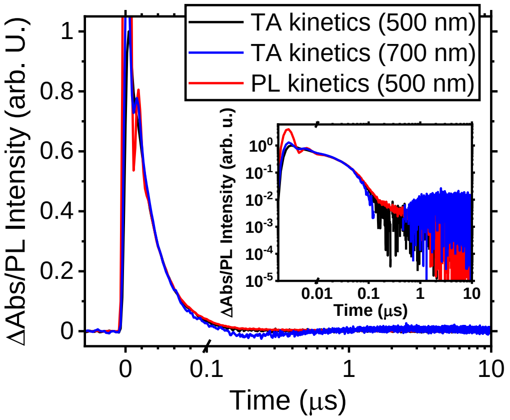

Figure S43 – Normalized nsTA decay traces of Au_{BCz}^{MAC} and 100 mM BIH in THF, 450 nm excitation (100 mL circulating flow cell). Two probe wavelength (500 nm – black, 700 nm – blue) with identical kinetics before 10 μs are shown. The time axis is lin-log with the break at 0.1 μs. The inset is the same traces displayed on a log-log plot demonstrating the PL intensity decays identically to the TA traces.

Section S9. Fitting Schemes for Quenching nsTA

For the nsTA data, the PDP fitting program was used again and where the fitting process was simpler as there is less overall spectral evolution. The IRF was determined by collecting pump light off a scattering solution and then fitting the resultant decay trace at the pump wavelength with a gaussian. The IRF, 4 ns is consistent with manufacturer specifications. The nsTA is therefore not sensitive to k_{rel} and simply reflects the combined S_1/T_1 decay, where the equilibrium between the S_1 and T_1 has been established well before 4 ns. A single compartment decay provides a strong constraint for the nsTA lifetime. The nsTA lifetimes obtained from fitting the unquenched data has good agreement with TCSPC measurements.

For the quenched studies, a second compartment (see Figure S44) was added to represent the quenched species, $M_{(amide)^+}^{carbene}$, with 2 decay pathways for the ${}^{S_1}/_{T_1}$ compartments: $1/\tau_{TADF}$ and $k_q[Q]$. The value for $1/\tau_{TADF}$ was obtained from and fixed to the unquenched nsTA data. As stated above, the $S_1 \rightarrow T_1$ ISC occurs within the instrument response for the nsTA with nearly complete extinguishing of the S_1. We can simplify Figure S13 to generate Figure S44a.

For the MePI data in toluene, we are assuming that the nonpolar solvent cage prevents the ions to diffuse away from each other, so we used a modified kinetic model to fit these data (Figure S44b). Since the fitting program could not separate these two species, we used the basis spectra of T_1 of $Au_{(BCz)^+}^{(MAC)^-}$ and $Au_{(BCz)^+}^{MAC}$, and by changing the ratios of the contribution of each species, the resulting summed spectra generated in Figure S50b was compared to that of Figure S50a. After finding the appropriate ratios, the k_2 and k_{-2} rates were back calculated, by assuming that k_2 is equal to $k_q[Q]$, using k_q from the THF data. The calculated rates are $k_2 = 2.8 \cdot 10^8$ s^{-1}, and $k_{-2} = 6.5 \cdot 10^8 s^{-1}$. A K of ~0.4 was discovered, indicating slight favor toward triplet reformation.

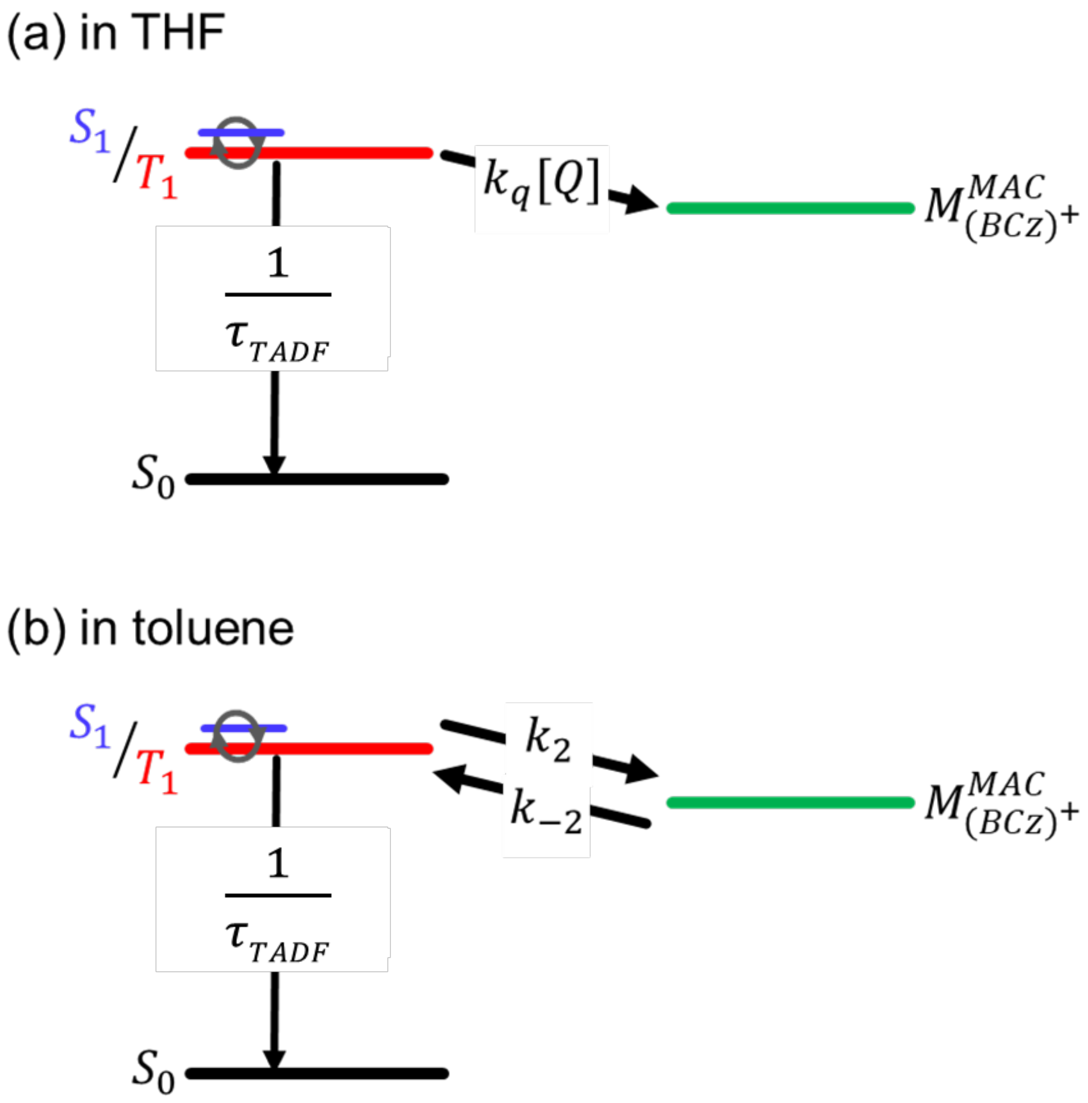

Figure S44 – Simplified kinetic model used for fitting the nsTA data, adapted from Figure S13, where states and processes are ignored to which the nsTA is insensitive. There are only two active compartments: $^{S_1}/_{T_1}$ and cation (or anion). For the unquenched experiments, only the $^{S_1}/_{T_1}$ compartment (red), is considered, while the cation (or anion) compartment (green) is added for the quenching studies. (a) In quenching studies in THF, conversion of $^{S_1}/_{T_1}$ to quenched ion was complete (b) whereas in toluene an excited state equilibrium was formed between cMa and quencher.

Section S10. Species Associated Decay Spectra (SADS) of nsTA spectra

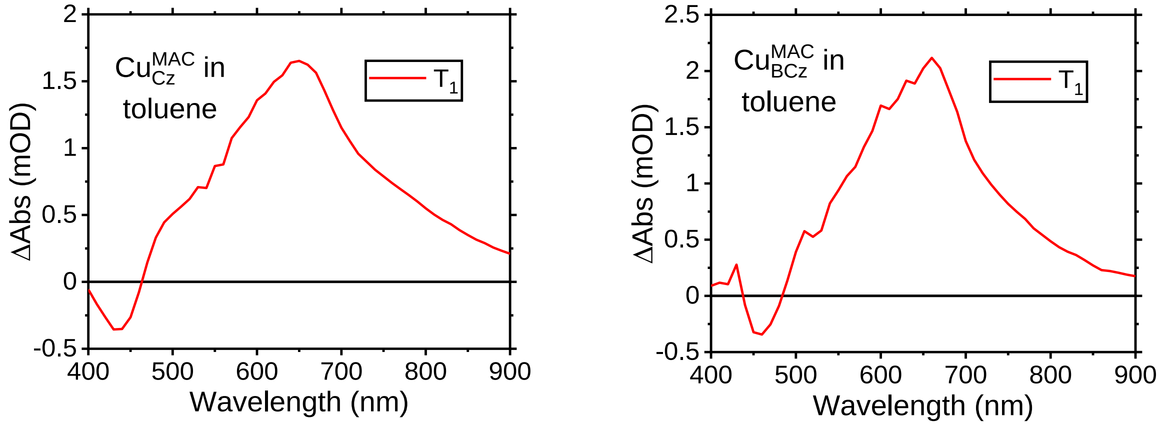

Figure S45 – SADS of Cu^{MAC}_{Cz} (left) and Cu^{MAC}_{BCz} (right) in toluene

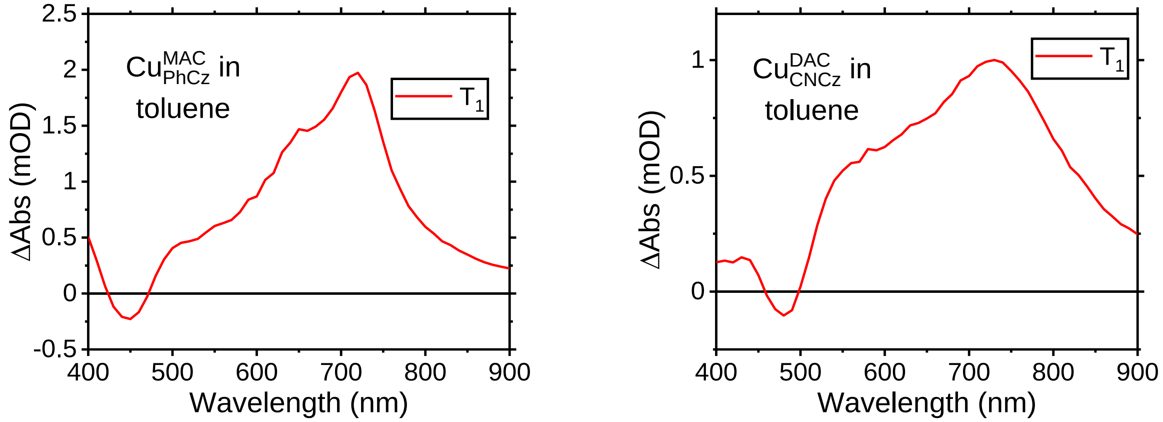

Figure S46 – SADS of Cu^{MAC}_{PhCz} (left) and Cu^{DAC}_{CNCz} (right) in toluene

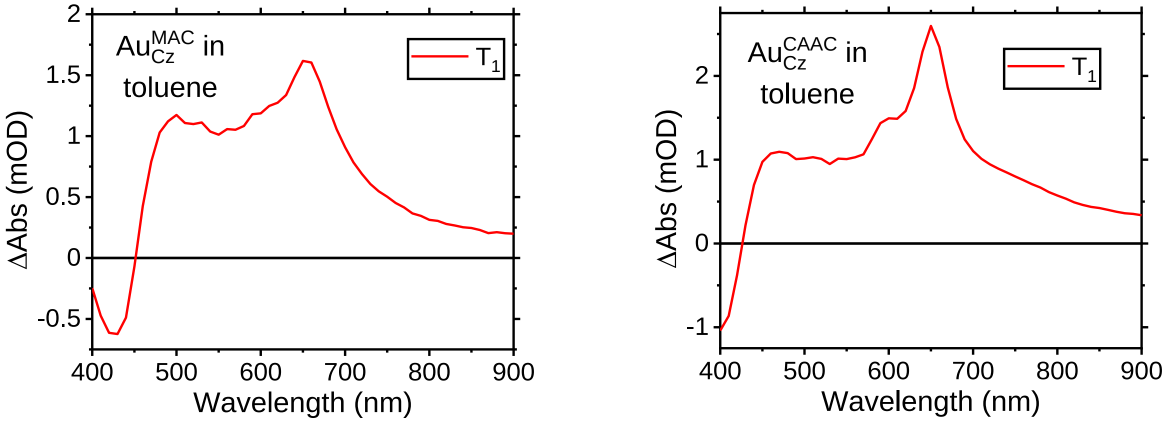

Figure S47 – SADS of Au^{MAC}_{Cz} (left) and Au^{CAAC}_{Cz} (right) in toluene

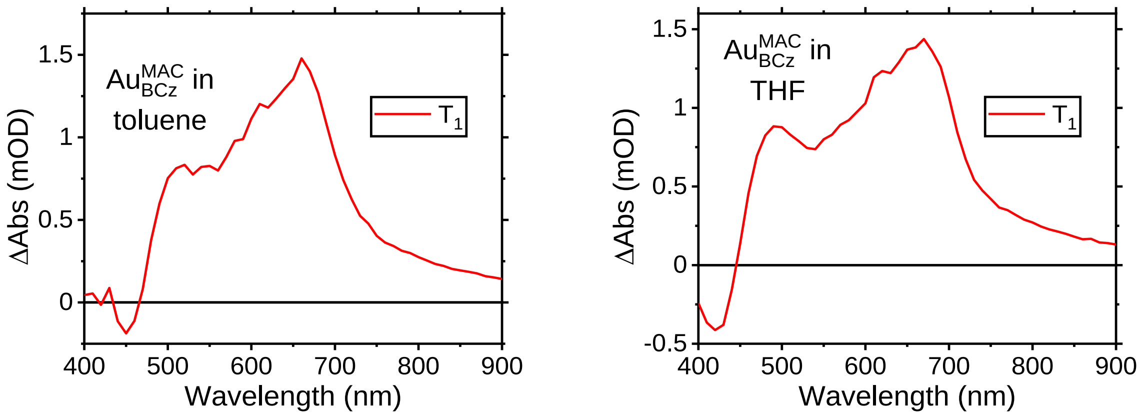

Figure S48 – SADS of Au^{MAC}_{BCz} in toluene (left) and in THF (right)

Section S11. SADS of nsTA Quenching Experiments

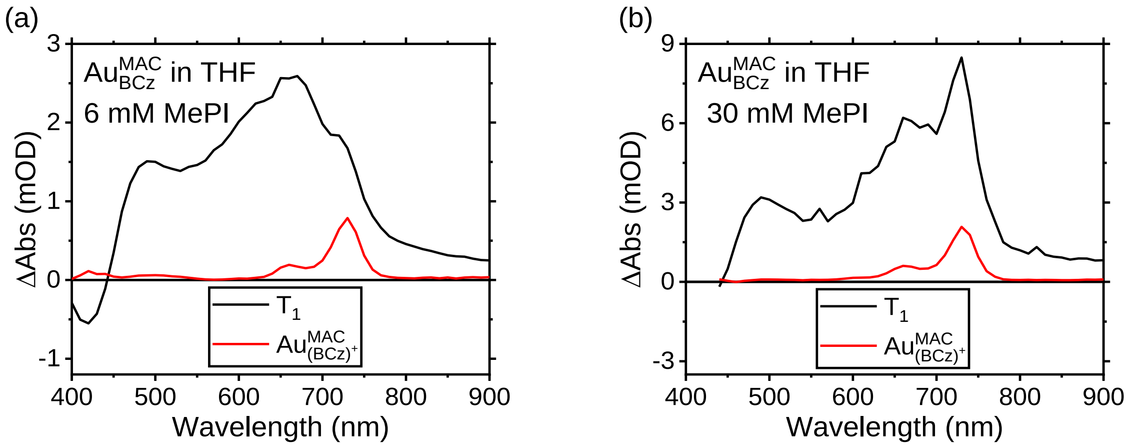

Figure S49 – nsTA SADS of Au_{BCz}^{MAC} in THF with MePI concentrations of (a) 6 mM and (b) 30 mM

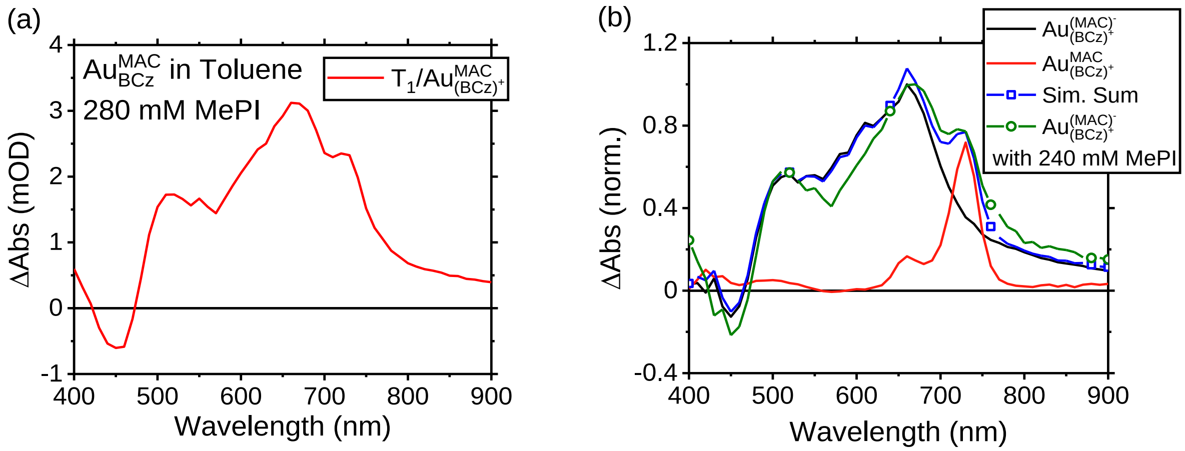

Figure S50 – nsTA SADS of Au_{BCz}^{MAC} with 280 mM MePI in toluene (a); the fit was unable to separate the two states, but we simulated the resulting spectra using the basis spectra of both compartments.

Section S12. Comparison of Triplet SADS from psTA and nsTA

In this section, the normalized SADS from the triplet spectrum for psTA (black) and nsTA (red) are depicted. The triplet spectra show good agreement. Deviations occur most strongly in the region of strongest PL and the 800 nm region. Deviations the PL region in the nsTA demonstrate imperfect PL subtraction. The region around 800 nm is more poorly determined in the psTA due to the overlap with the white light continuum driving wavelength and imperfect filtering in this spectral region.

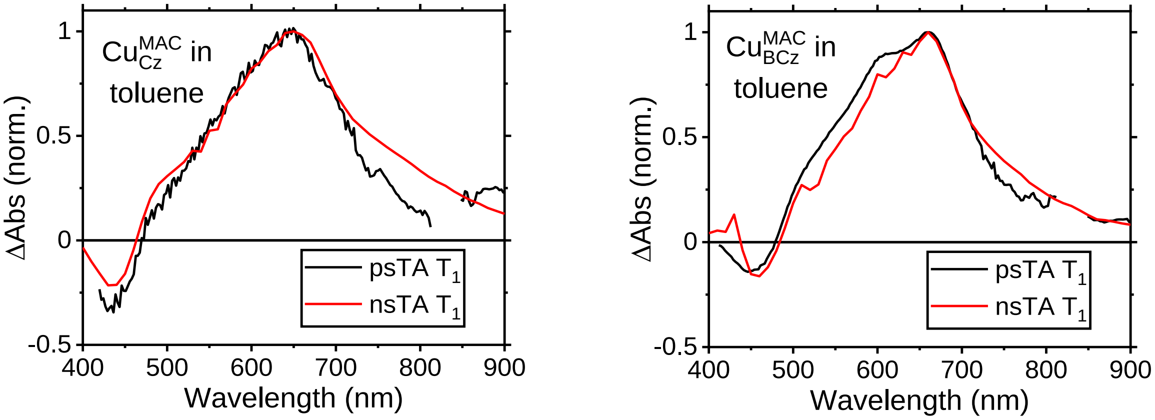

Figure S51 – SADS from psTA and nsTA of Cu_{Cz}^{MAC} (left) and Cu_{BCz}^{MAC} (right) in toluene

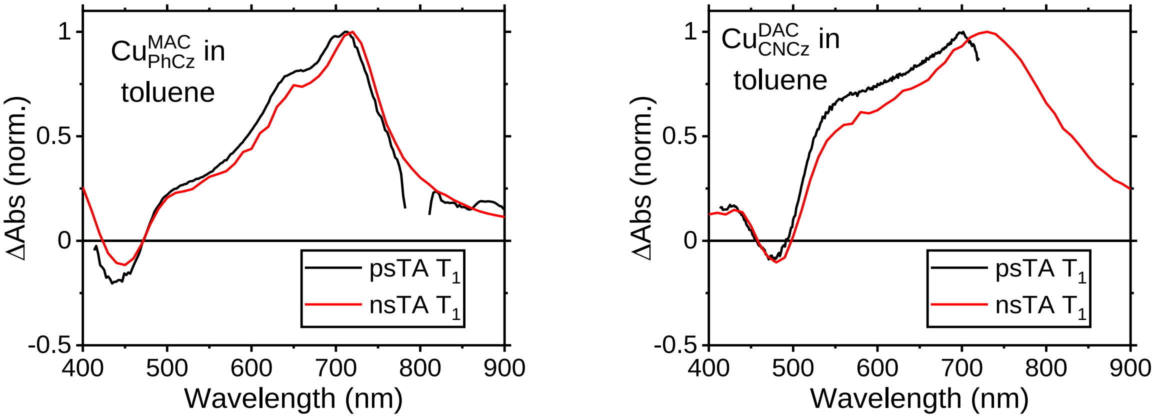

Figure S52 – SADS from psTA and nsTA of Cu_{PhCz}^{MAC} (left) and Cu_{CNCz}^{DAC} (right) in toluene

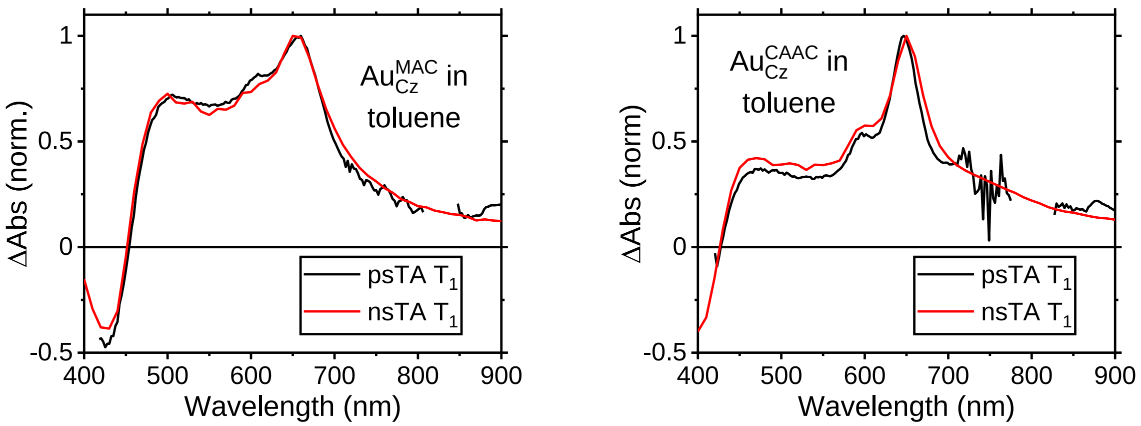

Figure S53 – SADS from psTA and nsTA of Au_{Cz}^{MAC} (left) and Au_{Cz}^{CAAC} (right) in toluene

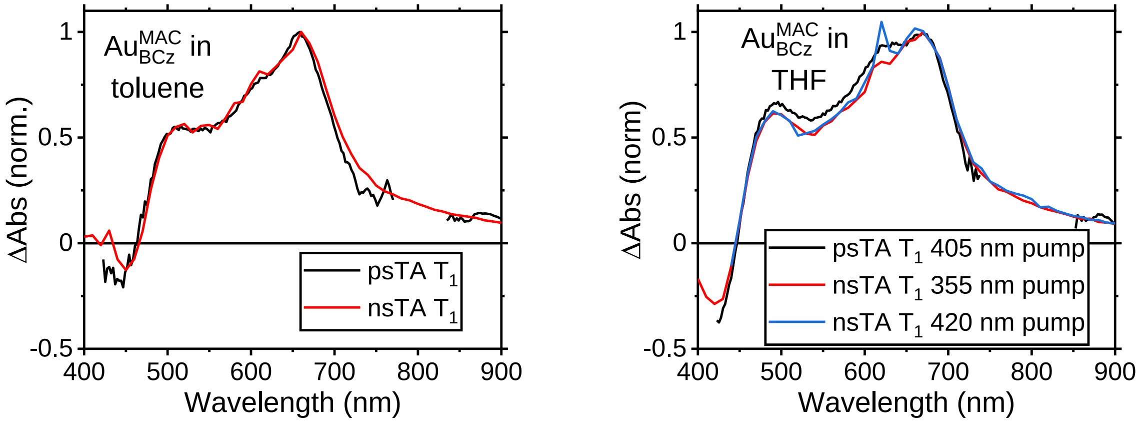

Figure S54 – SADS from psTA and nsTA of Au_{BCz}^{MAC} in toluene (left) and in THF (right). The right hand figure displays the SADS for the nsTA data with 405 nm pump (black), 355 nm pump (red) and 420 nm pump (blue).

Section S13. Simulation of the S_1 and T_1 ESA utilizing Pulse Radiolysis Spectra.

The spectra measured via PR can be utilized to simulate and positively assign the ESA portion of both of the S_1 and T_1 SADS extracted from the TA datasets. We extend this procedure for each of the copper complexes below examined by PR below. The two ionic molar absorptivity spectra are added together in a 1:1 ratio, with the cation spectra collected in o-xylene and the anion spectra collected in THF. The S_1 spectra are taken from the psTA SADS, while the T_1 spectra are taken from the SADS from the nsTA, with the exception of Cu_{Cz}^{CAAC} (**Figure 10a**) which is taken from the PR data, due to sample instabilities when excited with the 355 nm excitation source in our nsTA setup. Both SADS spectra have been normalized to the highest molar absorptivity value of the black trace. The absorption and emission spectra of each compound in toluene is reflected below the zero-line, normalized to an arbitrary 1000 M^{-1} cm^{-1}, in order to highlight the regions where GSB and SE (relevant for just the S_1) dominate the SADS extracted from the TA.

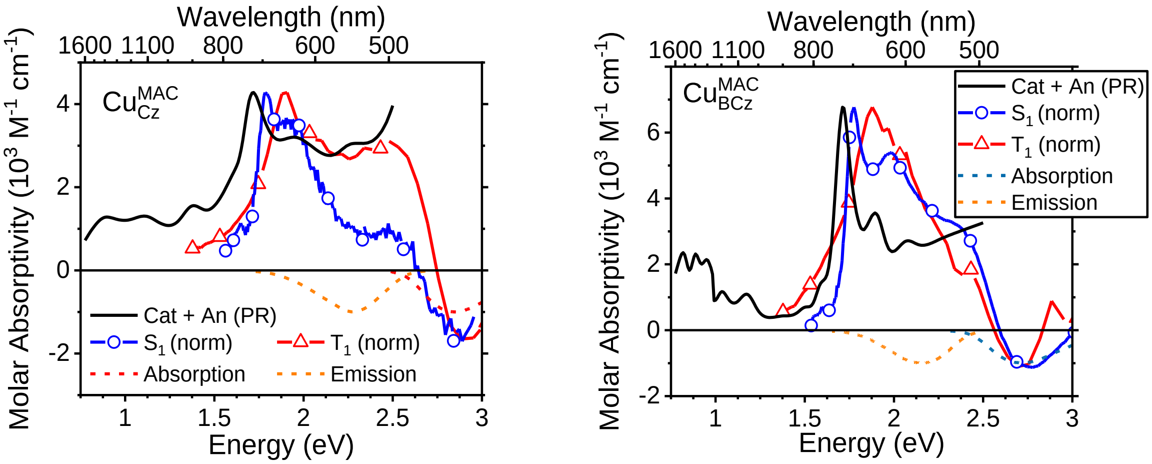

Figure S55 – The sum of the PR molar absorptivity plots (black) of Cu_{Cz}^{MAC} (left) and Cu_{BCz}^{MAC} (right) compared to the S_1 state (blue) and the T_1 state (red) from SADS analysis.

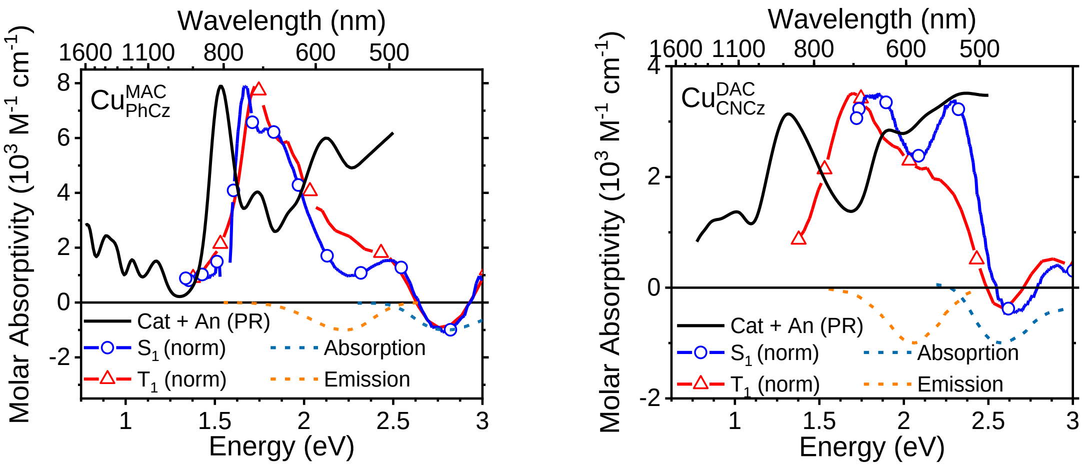

Figure S56 – The sum of the PR molar absorptivity plots (black) of Cu^{MAC}_{PhCz} (left) and Cu^{DAC}_{CNCz} (right) compared to the S_1 state (blue) and the T_1 state (red) from SADS analysis.

Section S14. Simulation of the S_1 and T_1 ESA utilizing Bulk Electrolysis Spectra

In this section, in a similar method to the previous section and the main manuscript, we utilize spectroelectrochemistry data to simulate what we might expect the ESA portion of the TA spectrum of Cu_{BCz}^{MAC} to see if it is also well approximated as a sum of absorption spectra of the oxidized and of the reduced complex. We start with the two BE spectra obtained for Cu_{BCz}^{MAC} in THF (**Figure 5**, **right**) and then compare their sum with the S_1 and T_1 spectra derived from the psTA and nsTA SADS, respectively (Figure S28, left and Figure S34, right). We are forced to use TA data from Cu_{BCz}^{MAC} in toluene as psTA data for Cu_{BCz}^{MAC} in THF was not collected. The emission and absorption spectra shown are also collected in toluene. While the absolute molar absorptivity of the Cu_{BCz}^{MAC} cation and anion is known from PR, the concentrations of the BE cation and anion are not known. Therefore, the anion and cation are scaled independently and then added together. The anion appears scaled by 4x compared to the cation – this best qualitatively matches the 500 to 700 nm region and the region >800 nm.

We can see that the S_1 SADS matches quite closely the sharpest peak, and vibrational sideband, in the ESA simulation based on the individual anion and cation absorption spectra. In the S_1 SADS, these two resolved peaks are shifted by about 30 nm to the blue, presumably by Coulombic interaction with the anion on the carbene not present in the electrochemically oxidized complex. Progressing to higher transition energies, the contribution of the stimulated emission in the S_1 SADS (and not to the T_1 SADS) explains the greater divergence to the simulated ESA, although it is not as dramatic as in Au_{BCz}^{MAC} (**Figure 10**). The T_1 SADS also exhibits peaks that can be associated with the carbazole cation, but they are shifted by ~ 80 nm to the blue and somewhat broadened, as found for Au_{BCz}^{MAC}. Both SADS have contributions from the bleach that make comparisons with the BE spectra harder in the 400 – 500 nm region.

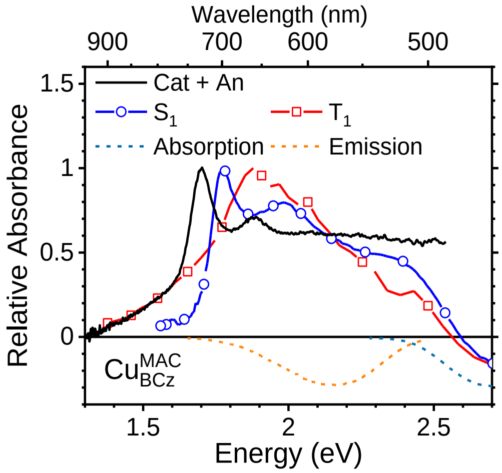

Figure S57 – The sum of the BE spectra (black) of Cu_{BCz}^{MAC} compared to the S_1 state (blue) and the T_1 state (red) from SADS analysis. The S_1 SADS is from psTA and the T_1 SADS is from nsTA.

Section S15. PL Correction in Magnitude Instruments

The Magnitude nsTA uses a different apparatus layout from the psTA experimental setup. Excitation of these cMa compounds leads luminescent emission in all directions including along the probe path; the latter leads to undesired detection of PL along with the probe light. When detected, PL is therefore additional light intensity, with its own time dependence, that therefore is a negative going artifact which can both qualitatively distort the spectrum and quantitatively modify the kinetics. In the most extreme cases, PL can completely overpower the TA spectrum leading to only a large, negative signal. So, to extract meaningful nsTA spectra, especially for optically emissive materials with long lifetimes, the PL must be removed.

Magnitude Instruments has provided a means for auto-PL subtraction by a simple button press. We have found that in some circumstances it under subtracts the amount of PL. We observed a time trace in the region of maximal PL emission that showed a fast decay, a rise then followed by another slower decay (Figure S58d, red trace). We know Au_{BCz}^{MAC} without quencher obeys single state first order decay in the ns-μs regime. It follows that time dynamics should be identical for every probed wavelength in absorption and the PL lifetime and wavelengths moving away from the PL maximum produce single exponential traces. On this basis, we consider, the red curve with its demonstrably different time behavior is considered to be distorted, presumably by imperfect correction for detected PL. Therefore, we have developed procedure to remove independently the PL artifact. This requires comparison to a psTA spectrum recorded at 1.5 ns (the longest time delay measurable in the psTA setup) for the same compound and solvent that is the best representation of the PL-free TA spectrum. This is because of the design of femtosecond pump probe: any (usually very small) pump-only PL signal will appear equally for all optical time delays. Therefore, the magnitude of this signal can be inspected at delays before time zero and then, if present at all, removed in equal measure from the entire TA dataset. For this reason, we consider the 1.5ns delay spectrum free of PL contamination. A linear factor now scales the overall amount of PL recorded in the nsTA so that we best match first (10 ns) transient spectrum for the nsTA to the 1.5 ns cut obtained from psTA. The effect of this

PL subtraction is demonstrated in Figure S58b. Here, most efficient PL removal occurs when $\boldsymbol{s} = 1.02$, different enough from the auto-subtracted feature to change the recovered dynamics. This procedure of scaling the PL subtraction in the nsTA to match the 1.5 ns trace of the psTA was carried through for nsTA spectra for each compound. For better PL subtraction, this value varies from 1.01 to 1.07 for each nsTA spectra. While the subtraction is imperfect as evidenced by temporal deviations of the nsTA traces from the PL at the same wavelength (Figure S58e, comparing blue and dotted black traces), this deviation is minor and the spectral shape is recovered.

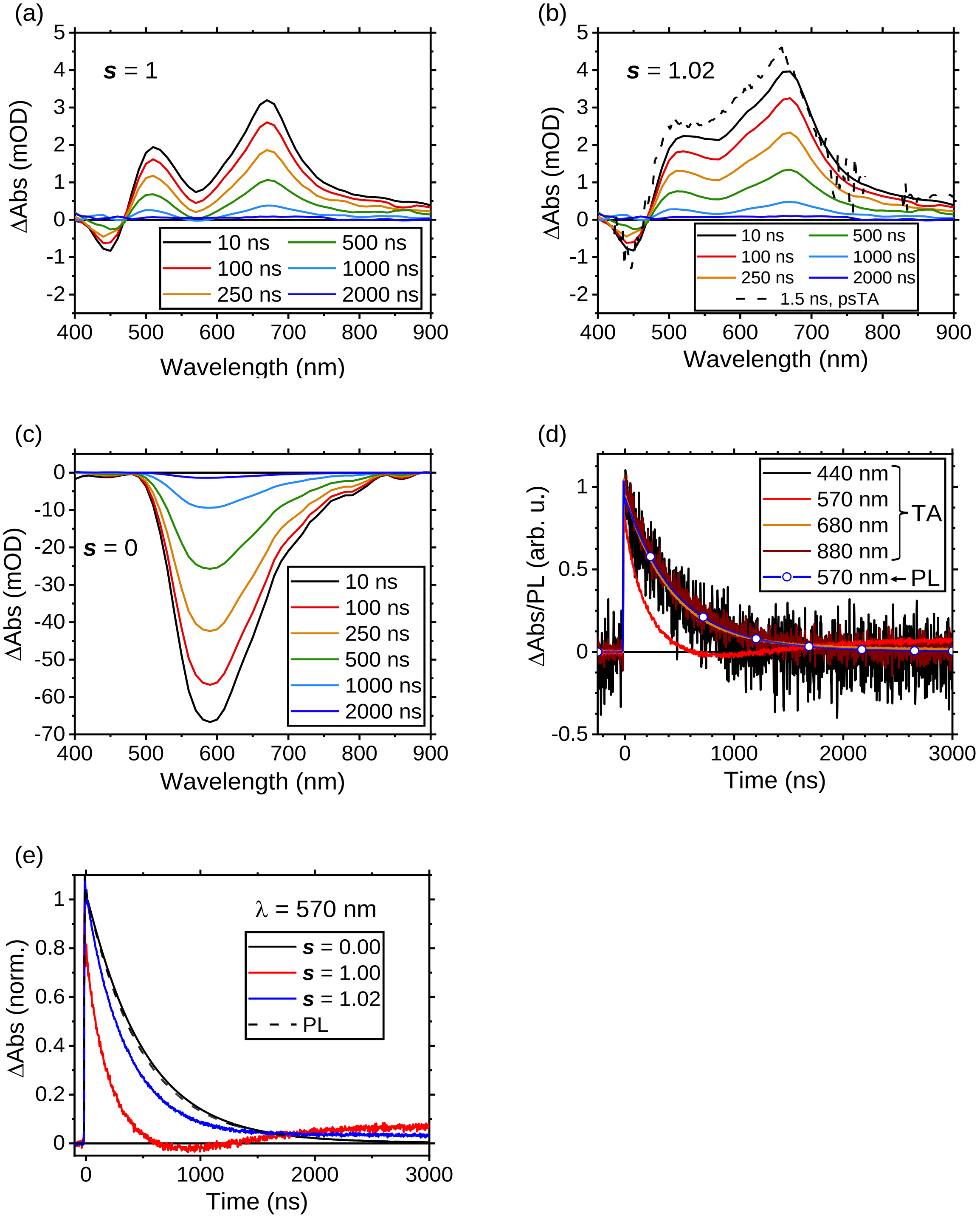

Figure S58 – nsTA of Au_{BCz}^{MAC} in toluene under varying values of PL subtraction parameter. (a) $\boldsymbol{s} = 1$ (auto subtraction), (b) $\boldsymbol{s} = 1.02$ (corrected), (c) $\boldsymbol{s} = 0$ (no PL subtraction). (d) Normalized nsTA time traces at 550 nm under varying $\boldsymbol{s}$ values. The trace at 440 nm for the GSB has been inverted for overlap with the other traces. The PL at 570 nm is plotted as the blue-circle line. (e) Time traces at 570 nm under PL correction with different s values.

Section S16. Photostability Measurements

The photostability of Cu_{BCz}^{MAC}, Cu_{PhCz}^{MAC}, and Au_{BCz}^{MAC} upon excitation with 460nm LEDs was evaluated in multiple solvent systems. Samples were prepared under oxygen free conditions in a sealed cuvette with a 1cm path length. The cuvette was placed in a pipe that was lined with 5mm round top, 460nm LEDs. The sample was exposed to radiation for varying amounts of time and the UV-Vis was subsequently measured shown in Figure S59(a-f). Emission of the sample was clearly visible during sample irradiation which indicated that the sample was being excited by the blue LEDs. The absorption spectra of Cu_{BCz}^{MAC}, Cu_{PhCz}^{MAC}, and Au_{BCz}^{MAC} in THF (Figure S59(a-c)) remain unchanged after 1 hour of irradiation. The persistence of the spectral intensity suggest that these complexes do not decompose under 460 nm irradiation in THF. Furthermore, Au_{BCz}^{MAC} was stable under 460nm irradiation in THF for 24 hours as demonstrated in Figure S59(c). The same analysis of the absorption spectra can be applied in Figure S59(d-f) which demonstrates that Au_{BCz}^{MAC} is stable under 460nm excitation for at least 1 hour in a 1:3 mixture of water to THF (by volume), and 1:1 mixtures of water and MeCN (by vol) for 1 hour of irradiation.

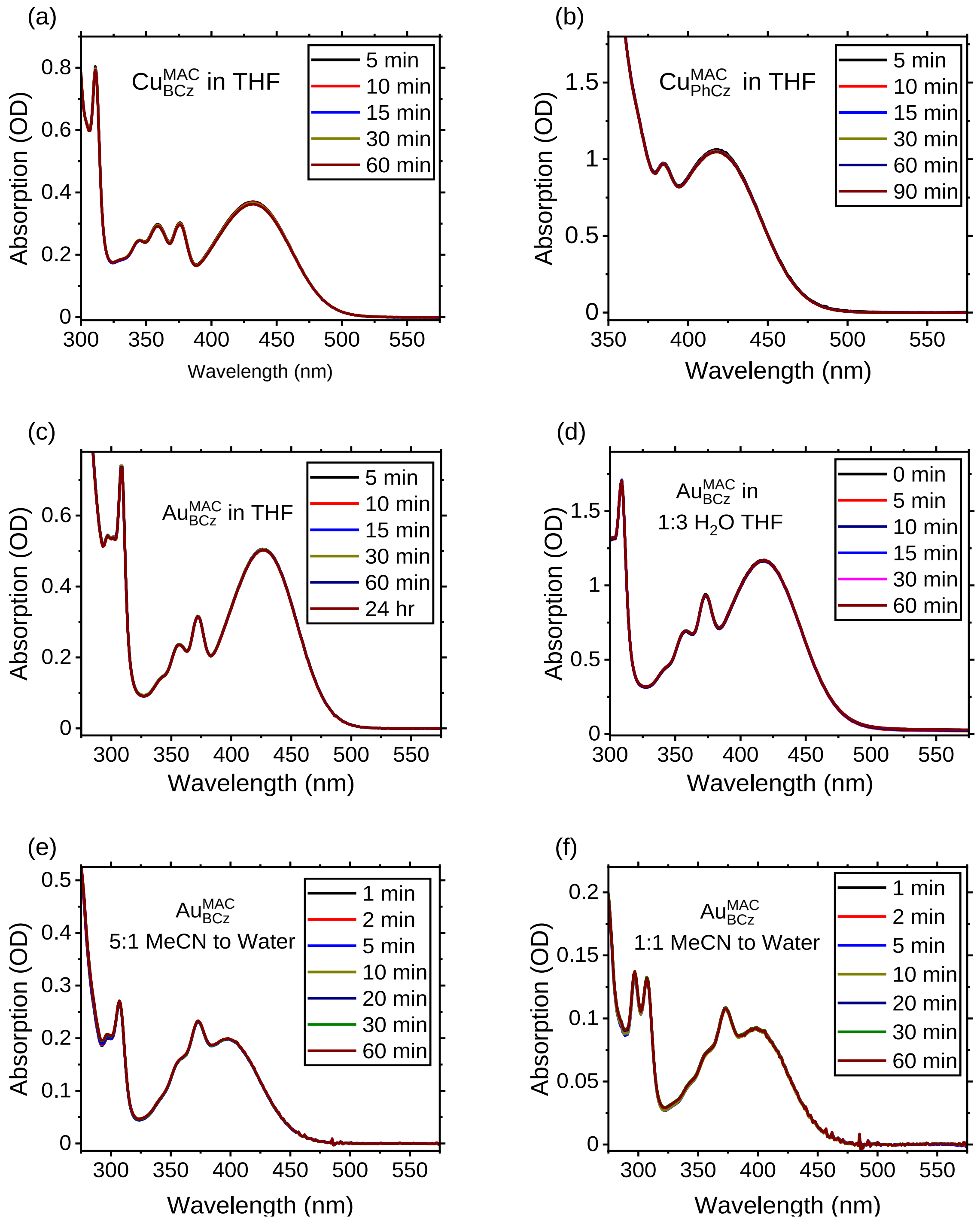

Figure S59 – Absorption spectra of cMa complexes under 460nm irradiation for varying amounts of time (a) Cu^{MAC}_{BCz} in THF, (b) Cu^{MAC}_{PhCz} in THF, (c) Au^{MAC}_{BCz} in THF, (d) Au^{MAC}_{BCz} in 1:3 Water to THF (by vol), (e) Au^{MAC}_{BCz} 5:1 MeCN to water (by vol), (f) Au^{MAC}_{BCz} 1:1 MeCN to water (by vol).

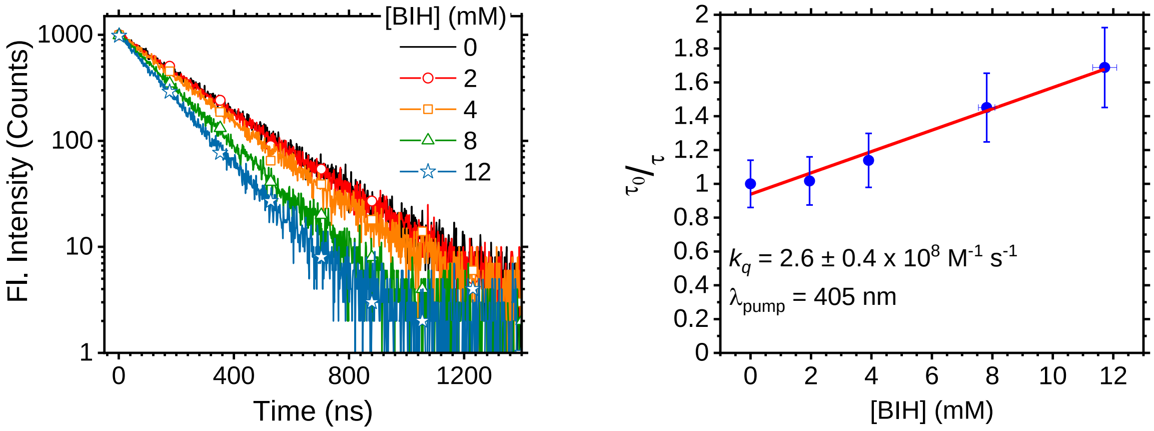

Figure S60 – Left) ns-TCSPC time traces with excitation at 405 nm for Au_{BCz}^{MAC} with various concentration of BIH in THF. Right) Stern-Volmer analysis of Au_{BCz}^{MAC} with various concentrations of BIH in THF. See Muniz *et al* reference for experimental procedures and analysis adopted here.

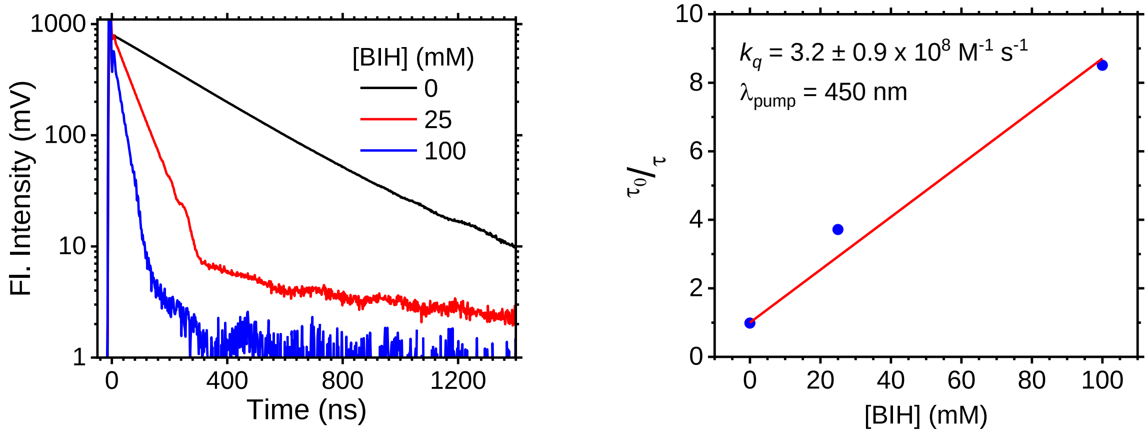

Figure S61 – Left) ns-TCSPC time traces with excitation at 450 nm for Au_{BCz}^{MAC} with various concentration of BIH in THF. Right) Stern-Volmer analysis of Au_{BCz}^{MAC} with various concentrations of BIH in THF. See Muniz *et al* reference for experimental procedures and analysis adopted here. This dataset was acquired with a Magnitude enVISion at UCR with 450 nm excitation.

Section S17. Molar Absorption Spectrum of BIH.

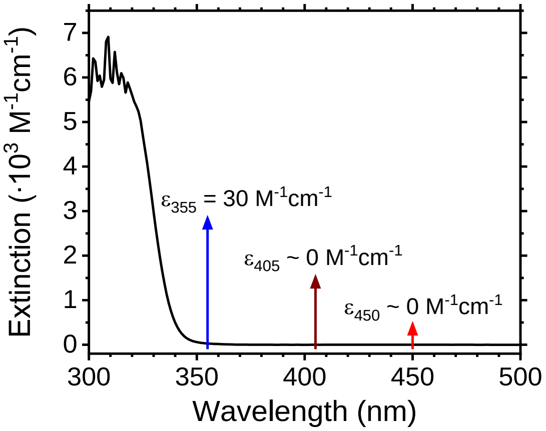

Figure S62 – Molar absorptivity spectrum of BIH in THF with absorptivities at the pump wavelengths indicated.

References

1. Muniz, C. N.; Archer, C. A.; Applebaum, J. S.; Alagaratnam, A.; Schaab, J.; Djurovich, P. I.; Thompson, M. E., Two-Coordinate Coinage Metal Complexes as Solar Photosensitizers. *Journal of the American Chemical Society* **2023,** *145* (25), 13846-13857, DOI: 10.1021/jacs.3c02825

2. Ekvall, K.; van der Meulen, P.; Dhollande, C.; Berg, L. E.; Pommeret, S.; Naskrecki, R.; Mialocq, J. C., Cross Phase Modulation Artifact in Liquid Phase Transient Absorption Spectroscopy. *Journal of Applied Physics* **2000,** *87* (5), 2340-2352, DOI: 10.1063/1.372185

3. Lorenc, M.; Ziolek, M.; Naskrecki, R.; Karolczak, J.; Kubicki, J.; Maciejewski, A., Artifacts in Femtosecond Transient Absorption Spectroscopy. *Applied Physics B: Lasers and Optics* **2002,** *74* (1), 19-27, DOI: 10.1007/s003400100750

4. Chen, X.; Larsen, D. S.; Bradforth, S. E.; van Stokkum, I. H., Broadband Spectral Probing Revealing Ultrafast Photochemical Branching after Ultraviolet Excitation of the Aqueous Phenolate Anion. *J Phys Chem A* **2011,** *115* (16), 3807-19, DOI: 10.1021/jp107935f
